# Inhibiting EGFR/HER-2 ameliorates neuroinflammatory responses and the early stage of tau pathology through DYRK1A

**DOI:** 10.3389/fimmu.2022.903309

**Published:** 2022-10-20

**Authors:** Jieun Kim, Su-Jin Kim, Ha-Ram Jeong, Jin-Hee Park, Minho Moon, Hyang-Sook Hoe

**Affiliations:** ^1^ Department of Neural Development and Disease, Korea Brain Research Institute (KBRI), Daegu, South Korea; ^2^ Department of Biochemistry, College of Medicine, Konyang University, Daejeon, South Korea; ^3^ Department of Brain & Cognitive Sciences, Daegu Gyeongbuk Institute of Science & Technology (DGIST), Daegu, South Korea

**Keywords:** LPS, NLRP3, Akt, microglia, FAK, varlitinib, tau, DYRK1A

## Abstract

The FDA-approved EGFR/HER2 inhibitor varlitinib inhibits tumor growth and is used in cancer treatment. However, the neuroinflammatory response associated with EGFR/HER2 and its underlying mechanism have not been elucidated. This study evaluates the impact of varlitinib on LPS- and tau-mediated neuroinflammatory responses for the first time. In BV2 microglial cells, varlitinib reduced LPS-stimulated *il-1β* and/or *inos* mRNA levels and downstream AKT/FAK/NF-kB signaling. Importantly, varlitinib significantly diminished LPS-mediated microglial *nlrp3* inflammasome activation in BV2 microglial cells. In primary astrocytes, varlitinib downregulated LPS-evoked astroglial *il-1β* mRNA levels, AKT signaling, and *nlrp3* inflammasome activation. In LPS-treated wild-type mice, varlitinib significantly reduced LPS-stimulated glial activation and IL-1β/NLRP3 inflammasome formation. Moreover, varlitinib significantly reduced micro- and astroglial activation and tau hyperphosphorylation in 3-month-old tau-overexpressing PS19 mice by downregulating tau kinase DYRK1A levels. However, in 6-month-old tau-overexpressing PS19 mice, varlitinib only significantly diminished astroglial activation and tau phosphorylation at Thr212/Ser214. Taken together, our findings suggest that varlitinib has therapeutic potential for LPS- and tau-induced neuroinflammatory responses and the early stages of tau pathology.

## Introduction

Neuroinflammation is an inflammatory response within the central nervous system (CNS) and is mediated by the release of cytokines, chemokines, and reactive oxygen species by CNS glia (1). CNS glia act as a shield against pathogens and pathogen-produced molecules like lipopolysaccharide (LPS) as well as neurodegenerative diseases such as Alzheimer’s disease (AD). Glia mainly comprise two types of cells: microglia and astrocytes. Microglia are found throughout the brain and play important roles in tissue maintenance and scanning for pathogens and cellular debris (2). Astrocytes are crucial regulators of the innate immune response, but their role in neuroinflammation in the CNS remains controversial (3). Both types of glial cells are capable of neurotoxic and neuroprotective phenotypes. The neuroprotective phenotype encompasses multiple reactive phenotypes that are related to the stage of the neuroinflammatory response.

LPS evokes neuroinflammation in mammals by stimulating toll-like receptor 4 (TLR4) signal transduction (4). LPS-induced TLR4 stimulation activates the transcription factor nuclear factor kappa B (NF-kB), which increases glial expression of NLR family pyrin domain containing 3 (NLRP3) and pro-interleukin 1β (pro-IL-1β). The upregulation of these two proteins facilitates NLRP3 inflammasome assembly by enhancing auto-oligomerization, leading to severe LPS-induced neuroinflammation (5). Recent studies have reported that the induction of the proinflammatory cytokines IL-1β, IL-6, and COX-2 by LPS promotes the formation of the NLRP3 inflammasome in BV2 microglia and mouse primary astrocytes (6–8). These observations indicate that NLRP3, pro-IL-1β, and IL-1β could be important targets for therapeutic drugs for neuroinflammation.

AD is a neurodegenerative disease that is correlated with neuroinflammation (2). Both amyloid β (Aβ) accumulation and neurofibrillary tangle (NFT) formation are hallmarks of AD. NFTs are the result of abnormal tau protein fiber accumulation due to hyperphosphorylation of tau protein in response to chronic neuroinflammation (9). Hyperphosphorylation of tau protein at specific residues contributes to the initiation of neuroinflammation in the early stage of AD (9, 10). In tau-overexpressing transgenic (Tg) PS19 mice, neural accumulation of phosphorylated tau and glial activation are initiated in the entorhinal cortex and dorsal hippocampus (11). In addition, hyperphosphorylated AT100 (tau phosphorylated at Thr212/Ser214) and AT8 (tau phosphorylated at Ser202/Thr205) are increased in reactive microglia in tau-overexpressing Tg PS19 mice (12). Therefore, therapies that promote the recovery of reactive microglia and astrocytes in AD patients might diminish tauopathy in response to neuroinflammation. However, there are few studies of therapeutic targeting of tau phosphorylation-related neuroinflammation in the early stage of tau pathology.

The small molecule varlitinib is an EGFR/HER2 (epidermal growth factor receptor) inhibitor that is FDA-approved for the treatment of breast cancer and is known to cross the blood–brain barrier (13). The breast cancer efficacy of varlitinib has been confirmed experimentally both *in vitro* and *in vivo* (14, 15). Numerous studies have explored the anticancer effects of varlitinib, but none have examined the impact of varlitinib on the neuroinflammatory response and tauopathy-associated neuroinflammation. Here, we examined the effects of varlitinib on neuroinflammatory responses induced by LPS, which elicits tau hyperphosphorylation and neurodegeneration, *in vitro* and *in vivo*. We found that post-treatment with varlitinib decreased LPS-mediated *il-1β* and *inos* mRNA levels in BV2 microglial cells, while pretreatment with varlitinib only significantly reduced LPS-stimulated *il-1β* mRNA levels. In addition, post-treatment with varlitinib significantly diminished AKT/FAK/NF-kB signaling by reducing activation of the NLRP3 inflammasome in BV2 microglial cells. Moreover, varlitinib suppressed the LPS-induced mRNA expression of the proinflammatory cytokines *il-1β* and *nlrp3* as well as downstream AKT signaling in primary astrocytes. In wild-type mice, varlitinib significantly reduced LPS-stimulated glial activation and downregulated LPS-evoked IL-1β/NLRP3 expression. Importantly, in 3-month-old tau-overexpressing PS19 mice, varlitinib significantly decreased tau-induced gliosis, tau hyperphosphorylation, and tau kinase dual specificity tyrosine phosphorylation regulated kinase 1A (DYRK1A) levels. However, in 6-month-old tau-overexpressing PS19 mice, the effects of varlitinib on tau-mediated gliosis, tau hyperphosphorylation, and DYRK1A levels were smaller than those in 3-month-old tau-overexpressing PS19 mice. Overall, the present study reveals that varlitinib has therapeutic effects on neuroinflammation and tau-related disease by inhibiting neuroglial NLRP3 inflammasome assembly and tau kinase DYRK1A, respectively.

## Materials and methods

### Ethics statement

All experiments were approved by the institutional biosafety committee (IBC) and performed in accordance with approved animal protocols of the Korea Brain Research Institute (KBRI, approval nos. IACUC-19-00042 and IACUC-19-00049).

### Reagents

Varlitinib (Cayman Chemical, Ann Arbor, MI, USA; Cat. No. 10009644) was used at a dose of 5 μM (1% DMSO) *in vitro* or 20 mg/kg (intraperitoneal (i.p.) injection in 5% DMSO, 10% PEG, 20% Tween 80) *in vivo*. The TLR4 inhibitor TAK-242 (Calbiochem, La Jolla, CA, USA) was used at a dose of 500 nM (1% DMSO) *in vitro*. LPS from *Escherichia coli* (Sigma-Aldrich; Cat. No. L2630) was used at a concentration of 200 ng/ml (in PBS) *in vitro* or 250 μg/kg or 10 mg/kg (i.p., in PBS) *in vivo*.

### MTT assay

The MTT assay was conducted to assess the effect of varlitinib on BV2 microglial cell viability. Cells in 96-well plates (4x10^4^ cells/well) were incubated for 1 h in FBS-free medium and then treated for 24 h with varlitinib (0.1, 1, 5, 10, 25 or 50 μM) or vehicle (0.001, 0.01, 0.05, 0.1, 0.25, or 0.5% DMSO). In addition, the effects of longer treatment durations on cell viability were assessed by treating cells with 1% DMSO or 5 μM varlitinib for 24 h, 48 h, or 72 h. After treatment, the cells were incubated with MTT solution for 3 h, and the formazan product was dissolved in DMSO at room temperature for 20 min. Finally, the absorbance of formazan at 570 nm was measured in a microplate reader (SPECTROstar Nano, BMG Labtech, Germany) using a reference wavelength of 660 nm.

### BV2 microglial cell culture

The microglial cell line BV2 was a generous gift from Dr. Kyung-Ho Suk. The culture conditions were as follows: 37°C, 5% CO_2_, high-glucose DMEM (Invitrogen, Carlsbad, CA, USA), and 5% fetal bovine serum (FBS, Invitrogen).

### Mouse primary astrocytes

The effects of varlitinib on LPS-induced proinflammatory responses were assessed in mouse primary astrocytes isolated as previously described (6–8, 16–20). First, whole brains were dissected from C57BL6 mice on postnatal day 1 and filtered through nylon mesh (70 μm). The tissue was then cultured in low-glucose DMEM with 10% FBS, 100 U/mL penicillin, and 100 μg/mL streptomycin for 14 days. Primary microglial cells were detached by agitating the mixed glial cells at 200 rpm and room temperature for 12 h. Finally, the primary astrocytes were detached with trypsin-EDTA (0.25%) and centrifuged at 2000 rpm for 10 min three times before use.

### Reverse transcription PCR

RT-PCR was performed to assess the effects of varlitinib on LPS-evoked inflammatory cytokine levels in BV2 microglial cells and primary astrocytes. Total RNA extracted from the cells using QIAzol Lysis Reagent (Qiagen, Cat No. 79306) was used to synthesize cDNA by reverse transcription. RT-PCR was then performed using Prime Taq Premix (GeNet Bio) with the cDNA template as previously described (7, 8, 16). The products were electrophoretically separated on a 1.5% agarose gel stained with EcoDye (1:5000, Biofact, Daejeon, Korea). Images of the gels were evaluated using Fusion Capt Advance software (Vilber Lourmat, Eberhardzell, Germany).

### Real-time PCR

The mRNA levels of proinflammatory cytokines and *nlrp3* in BV2 microglial cells, primary astrocytes, and LPS-treated wild-type mice were measured by real-time PCR (6–8, 16–20). First, cDNA was synthesized with Superscript cDNA Premix Kit II (GeNet Bio). The cDNA was subsequently used in real-time PCR with Fast SYBR Green Master Mix (Thermo Fisher Scientific, CA, USA) and a QuantStudio™ 5 system (Thermo Fisher Scientific). For normalization, the cycle threshold (Ct) value of GAPDH was used. The fold change in treated cells (LPS+varlitinib or LPS) was calculated relative to the vehicle-treated control. Real-time PCR primers were used as previously described (7).

### *nlrp3* siRNA transfection

To test whether varlitinib alters LPS-stimulated neuroinflammatory responses by modulating NLRP3 inflammasome activation, BV2 microglial cells were transfected with *nlrp3* siRNA or scramble (control) siRNA (Dharmacon, Rafayett, CO, USA). First, siRNA at a final concentration of 30 nM in Opti-MEM (Thermo Scientific, Waltham, MA, USA) was incubated with 1 µL of Lipofectamine^®^ RNAiMAX (Thermo Scientific) for 40 min. Second, BV2 microglial cells in a 24-well cell culture plate (2 x 10^5^ cells/well) were transfected with the siRNA mixture for 24 h. Finally, the transfected cells were treated with 200 nM LPS or PBS for 30 min and 5 μM varlitinib or 1% DMSO for 23.5 h, and real-time PCR was performed using previously described primers (7).

### Immunocytochemistry

The effects of varlitinib on neuroinflammatory responses induced by LPS were assessed in BV2 microglial cells and mouse primary astrocytes fixed in 4% paraformaldehyde (PFA) for 10 min. The fixed cells were washed with 1x PBS three times and incubated overnight at 4°C with anti-p-EGFR, anti-p-AKT, anti-p-FAK, anti-p-NF-kB, or anti-GFAP antibodies in GDB buffer (0.1% gelatin, 0.3% Triton X-100, 16 mM sodium phosphate, pH 7.4, and 450 mM NaCl). The cells were subsequently washed three times with 1x PBS and incubated for 1 h at room temperature with Alexa Fluor 488- or Alexa Fluor 555-conjugated antibodies (1:200, Molecular Probes, USA). Images of cells mounted in DAPI-containing solution (Vector Laboratories, CA, USA) were obtained by a DMi8 fluorescence microscope (Leica Microsystems, Wetzlar, Germany) in a single plane. To measure p-EGFR, p-AKT^s473^, p-FAK, and p-NF-kB fluorescence intensity, we used Image J software. Briefly, the region of interest (ROI) of each cell was automatically measured by CD11b or GFAP fluorescence intensity using the thresholding tools of Image J software. The selected ROIs were overlaid on matching red fluorescence images (p-EGFR, p-AKT^s473^, p-FAK, and p-NF-kB), and the fluorescence intensity inside the overlaid ROIs was measured. The fluorescence intensity of p-EGFR, p-AKT^s473^, p-FAK, and p-NF-kB is presented as the measured red fluorescence intensity divided by the selected ROI area. The analysis of 5–10 individual images of each sample was performed in a blinded manner.

### Nuclear fractionation

Nuclear NF-kB levels were assessed in BV2 microglial cells. First, the cells were treated with 200 ng/ml LPS or PBS for 30 min and 5 μM varlitinib or 1% DMSO (vehicle) for 5.5 h. The treated cells were then lysed for 5 min in cytosol fractionation buffer (10 mM HEPES pH 8.0, 1.5 mM MgCl_2_, 10 mM KCl, 0.5 mM DTT, 300 mM sucrose, 0.5 mM PMSF and 0.1% NP-40). The lysate was centrifuged at 10,000 rpm and 4°C for 1 min, and the supernatant (cytosolic fraction) was removed. Nuclear fractionation buffer (10 mM HEPES pH 8.0, 100 mM KCl, 100 mM NaCl, 0.2 mM EDTA, 0.5 mM DTT, 0.5 mM PMSF and 20% glycerol) was added to the pellet, which was incubated on ice for 15 min and then centrifuged at 10,000 rpm and 4°C for 15 min. The nuclear fraction was used to detect nuclear NF-kB levels by western blot.

### Western blotting

For western blotting, BV2 microglial cells, primary astrocytes, or mouse brain tissues were lysed in ProPrep lysis buffer (iNtRON Biotechnology, Inc., Seongnam, Korea) and centrifuged at 12,000 rpm for 15 min. The protein concentration in the supernatant was quantified by reference to a standard solution of BSA. Next, 10 μg of protein was loaded onto an 8% SDS gel, separated by electrophoresis, and transferred to a polyvinylidene difluoride (PVDF) membrane. The membrane was blocked with 5% skim milk for 1 h at room temperature and then incubated overnight at 4°C with anti-p-AKT^ser473^ (1:1000, Cell Signaling), anti-AKT (1:1000, Cell Signaling), anti-p-FAK (1:1000, Cell Signaling), anti-FAK (1:1000, Cell Signaling), anti-NF-kB (1:1000, Cell Signaling), or anti-PCNA (1:1000, Santa Cruz). Finally, the membrane was incubated for 1 h with HRP-conjugated goat anti-mouse IgG or HRP-conjugated goat anti-rabbit IgG (both 1:1000, Enzo Life Sciences, Farmingdale, NY, USA), and ECL Western Blotting Detection Reagent was added for detection (GE Healthcare, Chicago, IL, USA). Fusion Capt Advance software (Vilber Lourmat) was employed to acquire and analyze images.

### Wild-type mice and tau-overexpressing PS19 mice

Three-month-old wild-type C57/BL6 male mice (25-30 g; Orient-Bio Company, Gyeonggi-do, Korea) were maintained under a 12 h photoperiod in a pathogen-free facility with free access to food and water. To determine the effects of varlitinib on neuroinflammation *in vivo*, wild-type mice exposed to LPS were pre- or post-treated with varlitinib. For varlitinib post-treatment, wild-type mice were i.p. injected with LPS (250 μg/kg) or PBS followed by varlitinib (20 mg/kg, i.p.) or vehicle (5% DMSO, 10% PEG, 20% Tween 80) daily for 7 consecutive days. For varlitinib pretreatment, mice were i.p. injected with varlitinib (20 mg/kg) or vehicle (5% DMSO, 10% PEG, 20% Tween 80) daily for 7 consecutive, and on the 7^th^ day, 10 mg/kg LPS or PBS was injected followed 30 min later by the final administration of varlitinib or vehicle. Eight hours after the final injection on day 7, mouse brains were perfused and fixed for immunofluorescence staining.

The effects of varlitinib on age-dependent tau pathology and tau-mediated neuroinflammatory responses were examined in 3-month-old or 6-month-old tau-overexpressing PS19 mice (B6;C3-Tg (Prnp-MAPT*P301S)PS19Vle/J; Stock No. 008169, Jackson Laboratory, Bar Harbor, ME, USA). Genomic DNA from a tail snip was used for genotyping. To minimize the effects of hormones, only male mice were used. Regardless of age, the mice received the treatment regimen daily for 14 consecutive days, which consisted of i.p. injection of varlitinib (20 mg/kg) or vehicle (5% DMSO, 10% PEG, 20% Tween 80). After the final injection on day 14, mouse brains were perfused and fixed for immunofluorescence staining. All animal experiments were performed in accordance with approved animal protocols and guidelines established by the Korea Brain Research Institute Animal Care and Use Committee (IACUC-19-00042, IACUC-19-00049).

### Immunofluorescence staining

After final injection, wild-type and tau-overexpressing PS19 mice were anesthetized using 2,2,2-tribromoethanol (150 mg/kg) (Sigma-Aldrich, Cat no. T48402) in 2-methyl-2-butanol (Sigma-Aldrich, Cat no. 152463) for 5 min, perfused with PBS for 5 min, and fixed with 4% paraformaldehyde (PFA) for 7 min using a Cole-Parmer PTFE-Tubing pump (CacheBy, Seoul, Korea, Cat no. EW-77912-10). After PFA fixation, brains were immediately post-fixed in 4% PFA for 2 days, which was then replaced with 30% sucrose in PBS solution for 2 days. Finally, the brains were embedded in OCT compound (Tissue-Tek, Sakura Finetek, Torrance, CA, USA) and sliced at a thickness of 30 μm at -23°C using a cryostat (Leica CM1850, Leica Biosystems, Buffalo Grove, IL, USA).

To validate tissue-specific fluorescence intensity, brain sections were washed 3 times with PBST (1% Triton-100 in PBS) for 5 min, blocked with 10% normal goat serum (NGS) in PBST for 2-4 h, and incubated with primary antibodies in PBST at 4°C for 24-48 h (**Table 1**). After incubation with primary antibodies, the brain tissue was washed 3 times with PBST and incubated with secondary antibodies for 2 h (**Table 2**). Finally, the sections were washed 4 times with PBST and mounted in VECTASHIELD^®^ Antifade Mounting Medium with DAPI (Vector Laboratories). To reduce individual variance and batch-to-batch effects, brain sections from each group (i.e., wild-type mice treated with vehicle, LPS, or LPS+varlitinib or tau-overexpressing PS19 mice treated with vehicle or varlitinib) were randomly selected for immunofluorescence staining. For each group, immunofluorescence staining was conducted using equivalent reagents, the same processing and fixation times, the same antibodies, and identical conditions were used to capture images.

**Table 1:** List of primary antibodies used for IF.

Primary antibodies				
Immunogen	Host species	Dilution	Manufacturer	Catalog no.
Iba-1	Rabbit	1:500	Wako	019-19741
GFAP	chicken	1:500	Millipore	AB5541
NLRP3	Goat	1:500	Novus	NBP2-12446
IL-1β	Rabbit	1:100	Abcam	AB9722
AT100	Mouse	1:200	Invitrogen	mn1020
AT180	Mouse	1:200	Invitrogen	mn1040
DYRK1A	Rabbit	1:200	Abcam	ab180910
p-CDK5	Rabbit	1:200	MyBioSource	MBS9601140
p-GSK3β	Rabbit	1:200	Abcam	ab75745

**Table 2:** List of secondary antibodies used for IF.

Secondary antibodies				
Immunogen	Host species	Dilution	Manufacturer	Catalog no.
Rabbit-555	Goat	1:200	Invitrogen	A21428
Rabbit-488	Goat	1:200	Invitrogen	A11008
Mouse-555	Goat	1:200	Invitrogen	A21422
Mouse-488	Goat	1:200	Invitrogen	A11001
Goat-488	Donkey	1:200	Invitrogen	A11055
Chicken-488	Goat	1:200	Abcam	ab150169

To assess the effects of varlitinib on glial activation, we measured Iba-l and GFAP fluorescence intensity. In addition, to investigate the effects of varlitinib on glial migration and morphology, we quantified the number of GFAP-positive cells and the percent GFAP-positive area, respectively (16, 21).

To quantify the levels of Iba-I, GFAP, IL-1β, NLRP3, AT100, AT180, DYRK1A, p-GSK3β, or pCDK5 in the cortex and hippocampus, the area of each region was measured by drawing a region of interest (ROI) in a DAPI fluorescence image using Image J software. The selected ROIs were overlaid on matching red or green fluorescence images, and the fluorescence intensity inside the overlaid ROIs was measured. The levels of these markers are presented as the measured fluorescence intensity divided by the selected ROI area as previously described (20). The parameters were calculated as follows: [Fluorescence intensity (% of control) = fluorescence intensity/ROI/average of control group*100]; [Positive number of cells (% of control) = number of Iba-1- or GFAP-positive cells/ROI/average of control group*100]; [Positive percent of area (% of control) = percent Iba-1- or GFAP-positive area/ROI/average of control group*100].

### Statistical analysis

Comparisons of two groups were performed with unpaired two-tailed t-tests with Welch’s correction. For multiple comparisons, parametric one-way ANOVA or non-parametric one-way ANOVA (Kruskal-Wallis test) (Prism 7, GraphPad Software, USA) followed by Tukey’s test (parametric) or Dunn’s test (non-parametric) was used, with significance set at p < 0.05. Means ± SEMs are presented (*p < 0.05, **p < 0.01, ***p < 0.001). Detailed information is listed in **Supplementary Tables 1** and **2**. All experiments were replicated two or three times, and sums of the data are presented in this manuscript.

## Results

### Post-treatment with varlitinib reduces LPS-induced *il-1β* and *inos* mRNA levels in BV2 microglial cells

**Figure 1:**
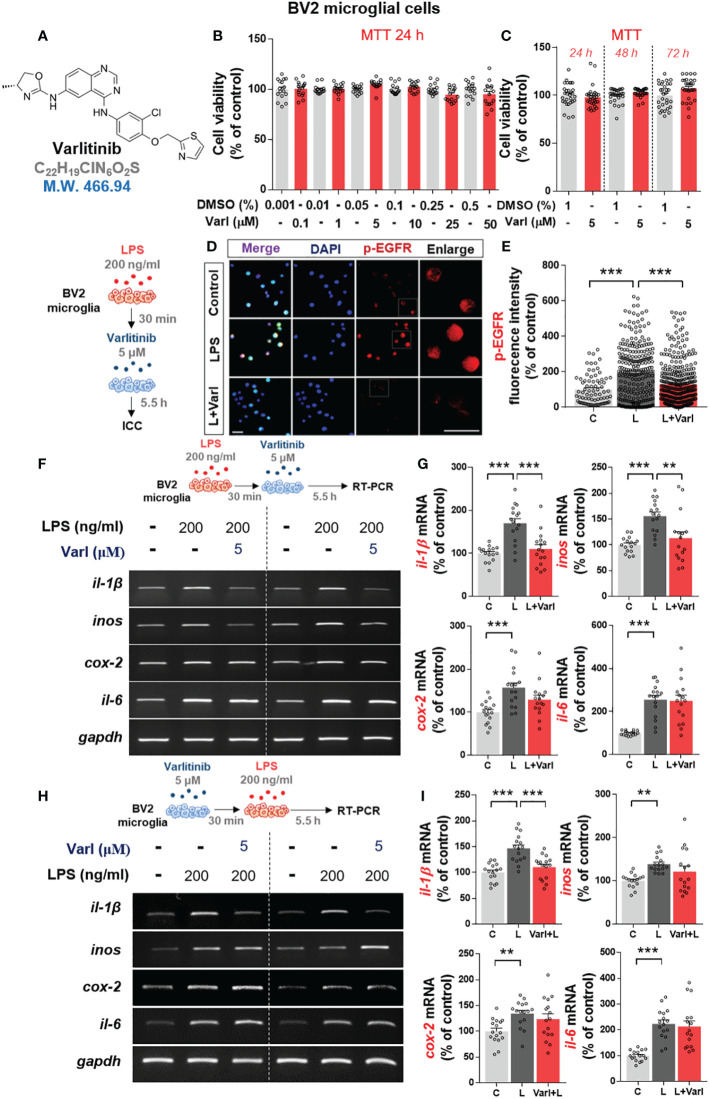
Varlitinib diminishes LPS-induced proinflammatory cytokine and mediator mRNA expression in BV2 microglial cells. **(A)** Structure of varlitinib. **(B)** Cells were treated with vehicle (DMSO) or varlitinib (0.1, 1, 5, 10, 25, 50 μM) for 24 h, and MTT assays were conducted (n = 16/group, 2 experimental replicates per dose). **(C)** Cells were treated with vehicle (1% DMSO) or varlitinib (5 μM) for 24 h, 48 h, and 72 h, and MTT assays were conducted (n = 30/group). **(D, E)** Cells were treated with LPS (200 ng/ml) or PBS for 30 min and varlitinib (5 μM) or vehicle (1% DMSO) for 5.5 h, and immunocytochemistry was conducted with anti-p-EGFR antibodies (C: n = 77; L: n = 263; L+Varl: n = 354, total number of examined cells). **(F, G)** Cells were treated as in **(D, E)**, and proinflammatory cytokine and *inos* mRNA levels were measured by RT-PCR (*il-1β, inos, cox-2*, and *il-6*: C: n = 16; L: n = 16; L+Varl: n = 16, independent biological replicates, 3 experimental replicates per group). **(H, I)** Cells were treated with varlitinib (5 μM) or vehicle (1% DMSO) for 30 min and LPS (200 ng/ml) or PBS for 5.5 h, and proinflammatory cytokine and *inos* mRNA levels were measured by RT-PCR (*il-1β, inos, cox-2*, and *il-6*: C: n = 16; L: n = 16; L+Varl: n = 16, independent biological replicates, 3 experimental replicates per group). ^**^ *p* < 0.01, ^***^ *p* < 0.001, C: Control; L: LPS; L+Varl: LPS+Varlitinib. Scale bar = 20 μm.

The EGFR/HER2 inhibitor varlitinib is used for the treatment of triple-negative breast cancer (TNBC), and its impact on LPS-induced neuroinflammation and the underlying mechanism of action have received little attention. Thus, we examined the effects of varlitinib on LPS-mediated proinflammatory responses. Before conducting further experiments, we assessed the impact of varlitinib on the viability of BV2 microglial cells by performing MTT assays of cells treated with vehicle or varlitinib (0.1, 1, 5, 10, 25, 50 μM) for 24 h. No decrease in cell viability was observed after incubation for 24 h with varlitinib concentrations of up to 50 μM (**Figures 1A, B**). The effects of longer treatment times on cell viability were assessed by treating cells with vehicle (1% DMSO) or 5 μM varlitinib for 24 h, 48 h, or 72 h. Subsequent MTT assays indicated that 5 μM varlitinib had no cytotoxicity at 72 h (**Figure 1C**).

The impact of varlitinib on p-EGFR levels was evaluated in BV2 microglial cells treated with LPS (200 ng/ml) or PBS for 30 min followed by varlitinib (5 μM) or vehicle (1% DMSO) for 5.5 h. Immunocytochemistry demonstrated that LPS-induced p-EGFR fluorescence intensity in BV2 microglial cells were significantly downregulated by varlitinib (**Figures 1D, E**).

To assess the curative and/or preventive anti-inflammatory effects of varlitinib *in vitro*, LPS-treated BV2 microglial cells were pre- or post-treated with varlitinib. For post-treatment, BV2 microglial cells were treated with LPS (200 ng/ml) or PBS for 30 min followed by varlitinib (5 μM) or vehicle (1% DMSO) for 5.5 h. Subsequent RT-PCR showed that post-treatment with varlitinib (as a curative treatment) significantly decreased LPS-induced *il-1β* and *inos* mRNA levels but not *cox-2* and *il-6* mRNA levels (**Figures 1F, G**).

Next, the effects of varlitinib pretreatment (as a preventive treatment) were assessed in BV2 microglial cells treated with varlitinib (5 μM) or vehicle (1% DMSO) for 30 min followed by LPS (200 ng/ml) or PBS for 5.5 h. RT-PCR showed that pretreatment with varlitinib only significantly reduced LPS-mediated *il-1β* mRNA levels (**Figures 1H, I**). These observations imply that varlitinib pre- and post-treatment differentially modulate LPS-stimulated proinflammatory cytokine levels in BV2 microglial cells.

### Varlitinib reduces LPS-mediated AKT/FAK signaling in BV2 microglial cells

**Figure 2:**
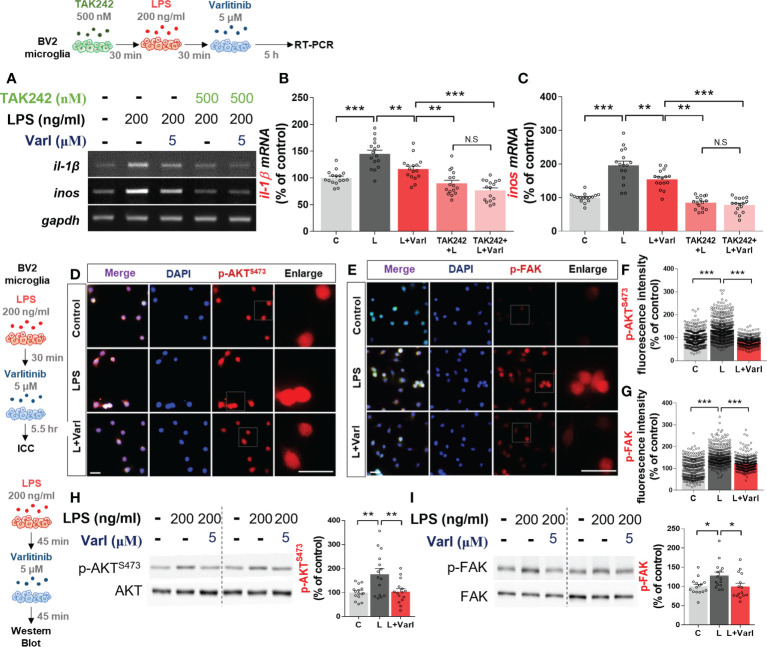
Varlitinib decreases LPS-induced AKT/FAK signaling in BV2 microglial cells. **(A–C)** Cells were treated with TAK242 (TLR4 inhibitor, 500 nM) or vehicle (1% DMSO) for 30 min, LPS (200 ng/ml) or PBS for 30 min, and varlitinib (5 μM) or vehicle (1% DMSO) for 5 h. *il-1β* and *inos* mRNA levels were analyzed by RT-PCR (*il-1β* and *inos*: C: n = 16; L: n = 16; L+Varl: n = 16, independent biological replicates, 3 experimental replicates per group). **(D–G)** Cells were treated with LPS (200 ng/ml) or PBS for 45 min and varlitinib (5 μM) or vehicle (1% DMSO) for 45 min, and immunostaining was conducted with anti-p-AKT and anti-p-FAK antibodies (p-AKT, C: n = 394; L: n = 460; L+Varl: n = 326: p-FAK, C: n = 331; L: n = 332; L+Varl: n = 358, total number of examined cells). **(H, I)** Cells were treated with LPS (200 ng/ml) or PBS for 45 min and varlitinib (5 μM) or vehicle (1% DMSO) for 45 min, and western blotting was conducted with anti-p-AKT and anti-p-FAK antibodies (p-AKT/p-FAK, C: n = 15; L: n = 15; L+Varl: n = 15, independent biological replicates, 3 experimental replicates per group). ^*^ *p* < 0.05, ^**^ *p* < 0.01, ^***^ *p* < 0.001, C: Control; L: LPS; L+Varl: LPS+Varlitinib. Scale bar = 20 μm.

Since varlitinib diminished LPS-stimulated proinflammatory cytokine levels *in vitro*, we next investigated whether varlitinib suppresses LPS-induced proinflammatory cytokine levels *via* TLR4 signaling. BV2 microglial cells were treated sequentially with TAK-242 (TLR4 inhibitor, 500 nM) or vehicle (1% DMSO) for 30 min, with LPS (200 ng/ml) or PBS for 30 min and finally with varlitinib (5 μM) or vehicle (1% DMSO) for 5 h. RT-PCR showed that similar to the results in **Figure 1**, LPS-induced *il-1β* and *inos* mRNA levels were significantly reduced in cells treated with varlitinib compared with LPS-treated cells (**Figures 2A–C**). By contrast, treatment with TAK-242, LPS, and varlitinib did not significantly alter LPS-induced *il-1β* and *inos* mRNA levels compared with treatment with TAK-242 and LPS (**Figures 2A–C**). These data imply that the downregulation of LPS-mediated *il-1β* or *inos* mRNA levels by varlitinib is partially dependent on TLR4 signaling in BV2 microglial cells.

AKT and FAK signaling are important contributors to LPS-induced inflammation *in vitro* and *in vivo* (16, 20). Thus, we tested the ability of varlitinib to modulate LPS-induced downstream AKT/FAK signaling *in vitro*. BV2 microglial cells were treated with LPS (200 ng/ml) or PBS for 30 min and varlitinib (5 μM) or vehicle (1% DMSO) for 5.5 h. Immunocytochemistry showed that varlitinib significantly reduced LPS-stimulated microglial p-AKT and p-FAK fluorescence intensity (**Figures 2D–G**). As a complementary approach, we performed western blotting and found that varlitinib significantly suppressed LPS-evoked microglial AKT and FAK phosphorylation (**Figures 2H, I**). These data indicate that varlitinib modulates LPS-stimulated microglial AKT and FAK signaling in BV2 microglial cells, which may play a role in the effects of varlitinib on LPS-induced proinflammatory responses.

### Varlitinib reduces LPS-stimulated nuclear NF-kB levels and NLRP3 inflammasome activation in BV2 microglial cells

**Figure 3:**
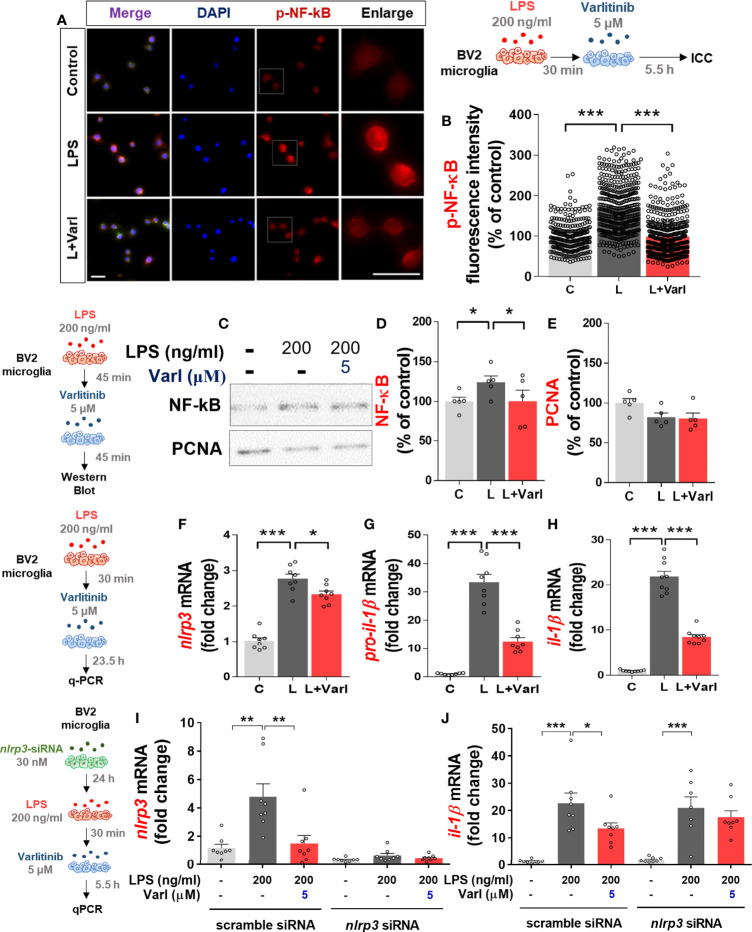
Varlitinib decreases LPS-evoked nuclear NF-kB phosphorylation and NLRP3 inflammasome activation in BV2 microglial cells. **(A, B)** Cells were treated with LPS (200 ng/ml) or PBS for 30 min and varlitinib (5 μM) or vehicle (1% DMSO) for 5.5 h, and immunostaining was conducted with an anti-p-NF-kB antibody (C: n = 274; L: n = 434; L+Varl: n = 481, total number of examined cells). **(C–E)** Cells were treated with LPS (200 ng/ml) or PBS for 30 min and varlitinib (5 μM) or vehicle (1% DMSO) for 5.5 h. The nuclear fraction was then used in western blotting with anti-NF-kB and anti-PCNA (nuclear marker) antibodies (C: n = 6; L: n = 6; L+Varl: n = 6, independent biological replicates). **(F–H)** Cells were treated with LPS (200 ng/ml) or PBS for 30 min and varlitinib (5 μM) or vehicle (1% DMSO) for 23.5 h, and proinflammatory cytokine levels were measured by real-time PCR (*nlrp3*, *pro-il-1β*, and *il-1β*: C: n = 8; L: n = 8; L+Varl: n = 8, independent biological replicates). (**I, J)** Cells were pretreated with *nlrp3* siRNA (30 nM) or scramble (control) siRNA for 24 h, treated with LPS (200 ng/ml) or PBS for 30 min, and treated with varlitinib (5 μM) or vehicle (1% DMSO) for 5.5 h. The mRNA levels of *nlrp3* and *il-1β* were analyzed by real-time PCR (*nlrp3* and *il-1β*: C: n = 8; L: n = 8; L+Varl: n = 8, independent biological replicates). ^*^ *p* < 0.05, ^**^ *p* < 0.01, ^***^ *p* < 0.001, C: Control; L: LPS; L+Varl: LPS+Varlitinib. Scale bar = 20 μm.

We and others have found that the EGFR inhibitor regorafenib decreases LPS-mediated NF-kB and STAT3 levels to ameliorate LPS-induced proinflammatory cytokine levels (19, 22). Therefore, we investigated whether varlitinib also affects LPS-mediated nuclear NF-kB levels in BV2 microglial cells treated as described above for the assessment of AKT/FAK phosphorylation. Immunocytochemistry demonstrated that varlitinib significantly reduced LPS-mediated nuclear p-NF-kB fluorescence intensity in BV2 microglial cells (**Figures 3A, B**). Further confirming these findings, nuclear fractionation of the treated cells demonstrated that varlitinib significantly diminished LPS-induced nuclear NF-kB activation in BV2 microglial cells (**Figures 3C–E**).

We then investigated the molecular mechanisms of the effects of varlitinib on the proinflammatory responses induced by LPS *in vitro*. BV2 microglial cells were pretreated with LPS (200 ng/mL) or PBS for 30 min as before, but the treatment time with varlitinib (5 μM) or vehicle (1% DMSO) was extended to 23.5 h. Real-time PCR indicated that varlitinib significantly diminished LPS-stimulated microglial *nlrp3*, *pro-il-1β*, and *il-1β* mRNA levels in BV2 microglial cells (**Figures 3F–H**).

We next examined whether the reduction in LPS-induced *il-1β* mRNA levels by varlitinib is involved in NLRP3 inflammasome activation. BV2 microglial cells transfected with *nlrp3* siRNA (30 nM) or scramble siRNA for 24 h were treated with LPS (200 ng/ml) or PBS for 30 min and varlitinib (5 μM) or vehicle for 23.5 h. Subsequent real-time PCR revealed that transfection with *nlrp3* siRNA significantly decreased *nlrp3* mRNA levels compared with transfection with scramble siRNA, indicating that *nlrp3* siRNA transfection was successful (**Figure 3I**). In addition, varlitinib did not alter LPS-induced *il-1β* mRNA levels in cells transfected with *nlrp3* siRNA compared with LPS-treated cells transfected with scramble siRNA (**Figure 3J**). These data suggest that varlitinib reduces NLRP3 inflammasome formation to downregulate proinflammatory cytokine *il-1β* mRNA levels in LPS-treated BV2 microglial cells.

### Varlitinib decreases LPS-stimulated astroglial proinflammatory cytokine *il-1β* mRNA levels and downstream AKT/NF-kB expression in primary astrocytes

**Figure 4:**
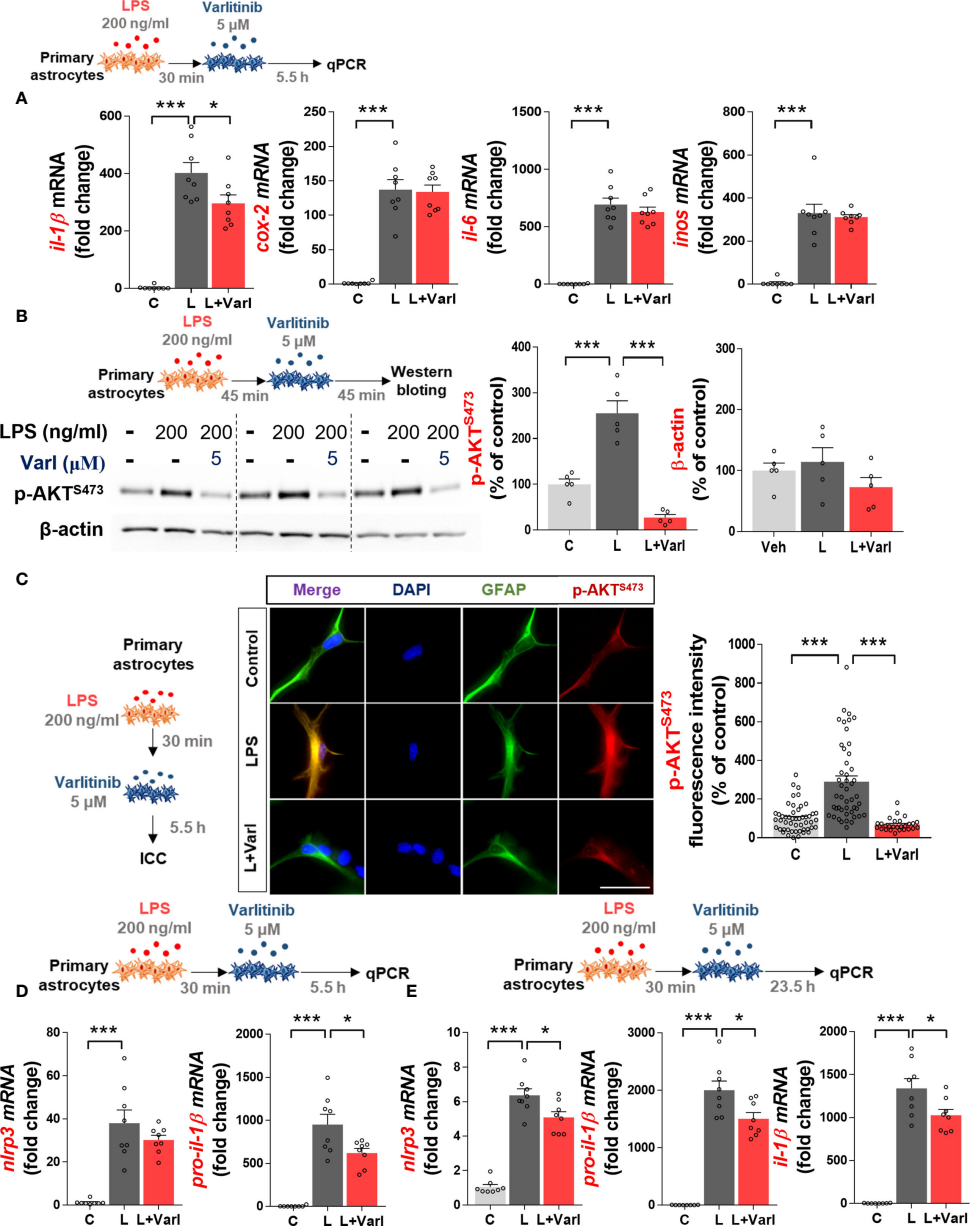
Varlitinib reduces LPS-stimulated astroglial *il-1β* mRNA levels, AKT signaling, and NLRP3 inflammasome activation in mouse primary astrocytes. **(A)** Cells were treated with LPS (200 ng/ml) or PBS for 30 min and varlitinib (5 μM) or vehicle (1% DMSO) for 5.5 h, and proinflammatory cytokine and mediator mRNA levels were measured by real-time PCR (*il-1β, cox-2, il-6*, and *inos*: C: n = 8; L: n = 8; L+Varl: n = 8, independent biological replicates). **(B)** Cells were treated with LPS (200 ng/ml) or PBS for 45 min and varlitinib (5 μM) or vehicle (1% DMSO) for 45 min, and western blotting was conducted with anti-p-AKT and anti-β-actin (C: n = 5; L: n = 5; L+Varl: n = 5, independent biological replicates) antibodies. **(C)** Cells were treated as in **(A)**, and immunocytochemistry was conducted with anti-p-AKT and anti-GFAP antibodies (C: n = 50; L: n = 47; L+Varl: n = 27, total number of examined cells). **(D)** Cells were treated as in **(A)**, and *nlrp3* and *pro-il-1β* mRNA levels were measured by real-time PCR (*nlrp3* and *pro-il-1β*: C: n = 8; L: n = 8; L+Varl: n = 8, independent biological replicates). **(E)** Cells were treated with LPS (200 ng/ml) or PBS for 30 min and varlitinib (5 μM) or vehicle (1% DMSO) for 23.5 h, and the expression of NLRP3 inflammasome-associated genes was measured by real-time PCR (*nlrp3, pro-il-1β,* and *il-1β*: C, n = 8; L, n = 8; L+Varl: n = 8, independent biological replicates). ^*^ *p* < 0.05, ^***^ *p* < 0.001, C: Control; L: LPS; L+Varl, LPS+Varlitinib. Scale bar = 20 μm.

Since varlitinib downregulated LPS-induced microglial proinflammatory responses, we assessed whether varlitinib regulates LPS-stimulated astroglial proinflammatory responses. Primary astrocytes were treated with LPS (200 ng/ml) or PBS for 30 min and varlitinib (5 μM) or vehicle (1% DMSO) for 5.5 h. Real-time PCR demonstrated that varlitinib significantly decreased the LPS-evoked increase in *il-1β* mRNA levels but not *cox-2*, *il-6*, or *inos* mRNA levels (**Figure 4A**). These data suggest that varlitinib specifically impacts LPS-mediated proinflammatory cytokine *il-1β* mRNA levels in primary astrocytes.

We then examined the effect of varlitinib on downstream AKT signaling in primary astrocytes, as varlitinib decreased LPS-induced microglial AKT signaling. Primary astrocytes were treated with LPS (200 ng/ml) or PBS for 45 min and varlitinib (5 μM) or vehicle (1% DMSO) for 45 min. Western blotting showed that varlitinib significantly downregulated LPS-mediated astroglial AKT phosphorylation (**Figure 4B**). To further confirm these findings, primary astrocytes treated with LPS (200 ng/ml) or PBS for 30 min and varlitinib (5 μM) or vehicle (1% DMSO) for 5.5 h were subjected to immunocytochemistry with anti-GFAP and anti-p-AKT antibodies. The results revealed that varlitinib significantly suppressed LPS-stimulated astroglial p-AKT fluorescence intensity (**Figure 4C**). These data indicate that varlitinib reduces LPS-mediated downstream AKT signaling in primary astrocytes.

### Varlitinib suppresses LPS-mediated NLRP3 inflammasome activation in primary astrocytes

In BV2 microglial cells, varlitinib diminished LPS-induced proinflammatory cytokine levels by inhibiting NLRP3 inflammasome activation. To determine whether these effects of varlitinib extend to primary astrocytes, cells were treated with LPS (200 ng/ml) or PBS for 30 min and varlitinib (5 μM) or vehicle (1% DMSO) for 5.5 or 23.5 h. Real-time PCR analysis showed that treatment with varlitinib for 5.5 h did not affect LPS-induced *nlrp3* mRNA levels in primary astrocytes but significantly reduced LPS-induced *pro-il-1β* mRNA levels (**Figure 4D**). By contrast, treatment with varlitinib for 23.5 h significantly reduced LPS-evoked astroglial *nlrp3*, *pro-il-1β*, and *il-1β* mRNA levels (**Figure 4E**), suggesting that varlitinib downregulates LPS-induced astroglial NLRP3 inflammasome activation in a time-dependent manner.

### Varlitinib treatment significantly reduces LPS-stimulated glial activation under chronic neuroinflammation conditions

To determine the effects of varlitinib on neuroinflammatory responses *in vivo*, we used LPS, which is known to induce neurodegenerative disease as well as neuroinflammation (23–26). Specifically, a recent study demonstrated that administration of 100 μg/kg LPS to wild-type mice increases tau phosphorylation, tau kinase p-GSK3β and p-CDK5 levels, and microglial activation (26). Another study found that the intra-frontal cortical and hippocampal injection of LPS significantly enhances tau hyperphosphorylation in rTg4510 mice (27). Based on these findings, we used an LPS-injected mouse model to determine the effects of varlitinib on tau-associated neuroinflammation and neuroinflammation-linked neurodegenerative disease.

**Figure 5:**
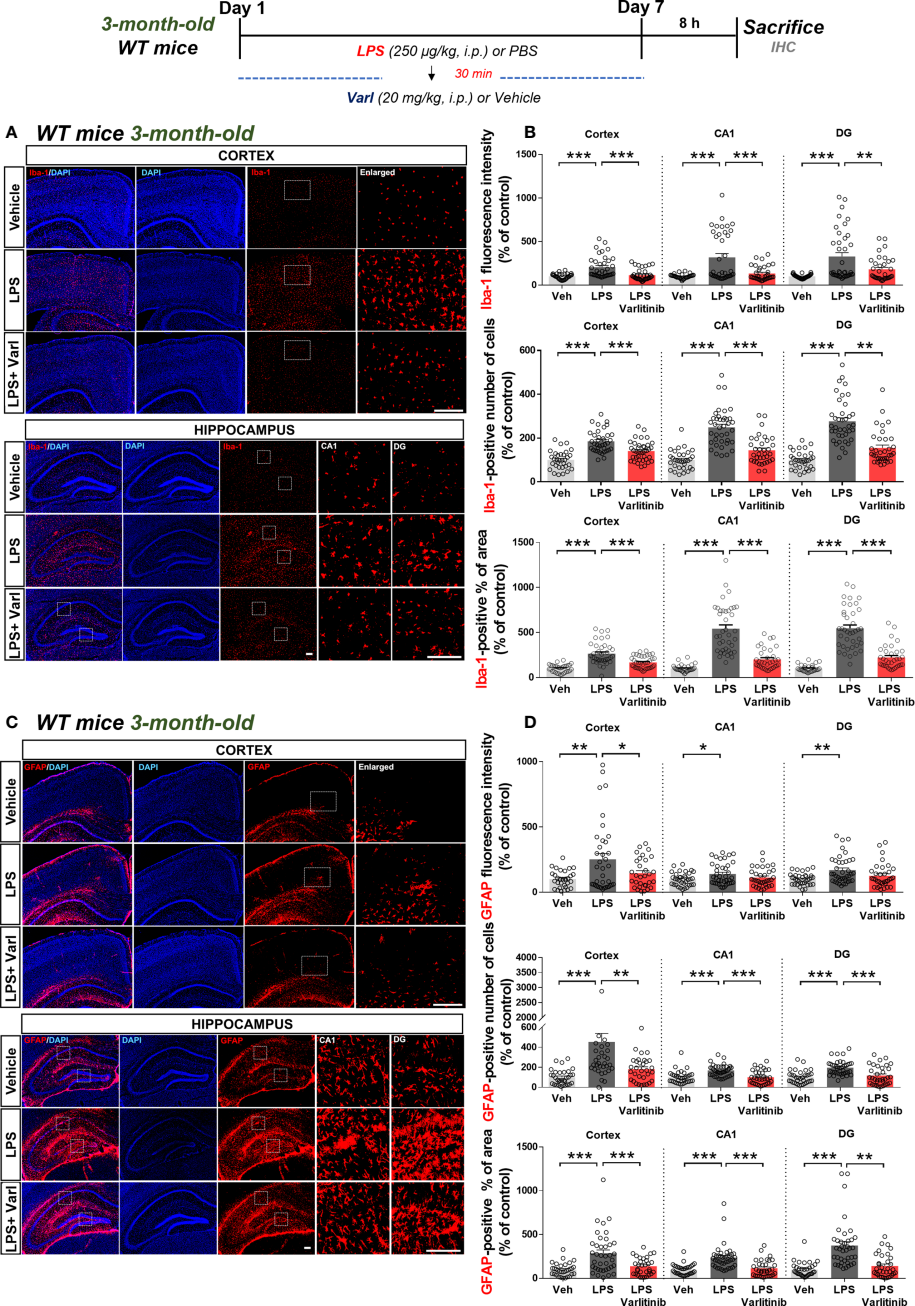
Varlitinib reduces LPS-stimulated glial activation in wild-type mice. **(A, C)** Wild-type mice were injected with LPS (250 μg/kg, i.p.) or PBS and, 30 min later, varlitinib (20 mg/kg, i.p.) or vehicle (5% DMSO, 10% PEG, 20% Tween-80, i.p.) daily for 7 days. On the 7^th^ day, 8 h after the LPS or PBS injection, immunofluorescence staining was performed with anti-Iba-1 and anti-GFAP antibodies. **(B, D)** Quantification of data from A and C (n = 8 mice/group, Veh: 32 brain sections; LPS: 38 brain sections; LPS+Varlitinib: 38 brain sections, 2 experimental replicates per group). ^*^ *p* < 0.05, ^**^ *p* < 0.01, ^***^ *p* < 0.001. Veh, Vehicle, Scale bar = 50 μm.

Since varlitinib affected LPS-evoked proinflammatory cytokine levels and downstream signaling *in vitro*, we next investigated whether varlitinib post-treatment regulates LPS-stimulated microgliosis and astrogliosis *in vivo.* To induce chronic neuroinflammation conditions, wild-type mice were injected daily for 7 days with a regimen consisting of LPS (250 μg/kg, i.p.) or PBS followed 30 min later by varlitinib (20 mg/kg, i.p.) or vehicle (5% DMSO, 10% PEG, 20% Tween-80). Eight hours after the final LPS or PBS injection on day 7, the mice were perfused and fixed, and brain sections were subjected to immunofluorescence staining with anti-Iba-1 or anti-GFAP antibodies. Under chronic neuroinflammation conditions, varlitinib post-treatment significantly inhibited microglial Iba-1 fluorescence intensity, the number of Iba-1-positive cells, and the percent Iba-1-positive area in the cortex and hippocampus (**Figures 5A, B**). In addition, post-treatment with varlitinib significantly downregulated astroglial GFAP fluorescence intensity in the cortex but not the hippocampus and significantly reduced the number of GFAP-positive cells and the percent GFAP-positive area in the cortex and hippocampus (**Figures 5C, D**) in LPS-treated wild-type mice. These data indicate that varlitinib post-treatment modulates LPS-mediated microgliosis more effectively than astrogliosis in wild-type mice under chronic neuroinflammation conditions.

### Varlitinib suppresses LPS-mediated IL-1β and NLRP3 levels in wild-type mice

**Figure 6:**
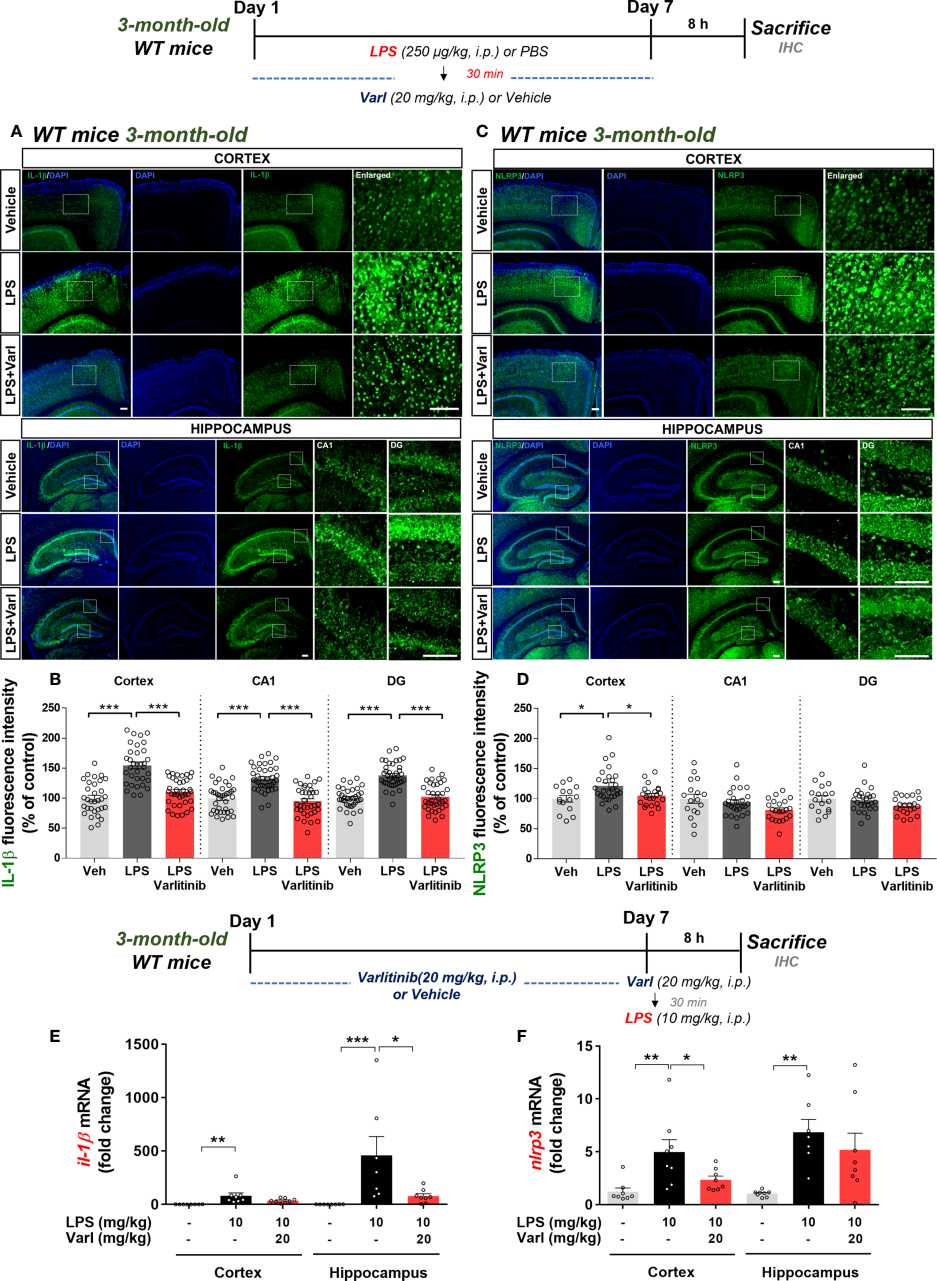
Varlitinib downregulates proinflammatory cytokine IL-1β levels and NLRP3 in LPS-treated C57BL/6 mice. **(A, C)** Wild-type mice were injected with LPS (250 μg/kg, i.p.) or PBS and, 30 min later, varlitinib (20 mg/kg, i.p.) or vehicle (5% DMSO, 10% PEG, 20% Tween-80, i.p.) daily for 7 days. On the 7^th^ day, 8 h after the LPS or PBS injection, immunofluorescence staining was conducted with anti-IL-1β and anti-NLRP3 antibodies. **(B, D)** Quantification of data from A and C (n = 4 mice/group, Veh: 15 brain sections; LPS: 28 brain sections; LPS+Varlitinib: 22 brain sections, Il-1β: 2 experimental replicates per group). **(E, F)** Wild-type mice were injected with varlitinib (20 mg/kg, i.p.) or vehicle (5% DMSO, 10% PEG, 20% Tween-80, i.p.) daily for 7 days. On day 7, the last varlitinib or vehicle injection was followed 30 min later by injection of LPS (10 mg/kg, i.p.) or PBS. Eight hours after the LPS or PBS injection, *nlrp3* and *il-1β* mRNA levels were measured by real-time PCR (*nlrp3* and *il-1β*: cortex: C: n = 8; L: n = 8; L+Varl: n = 8, hippocampus: C: n = 8; L: n = 8; L+Varl: n = 8). ^*^ *p* < 0.05, ^**^ *p* < 0.01, ^***^ *p* < 0.001. Veh, Vehicle; Scale bar = 50 μm.

To determine the effects of varlitinib on LPS-induced IL-1β levels and NLRP3 inflammasome activation under chronic neuroinflammation conditions, wild-type mice were injected with LPS (250 μg/kg, i.p.) or PBS followed by varlitinib (20 mg/kg, i.p.) or vehicle (5% DMSO, 10% PEG, 20% Tween-80) daily for 7 days as shown in **Figure 6**. Brain sections were immunostained with anti-IL-1β or anti-NLRP3 antibodies. Under these chronic neuroinflammation conditions, varlitinib post-treatment significantly decreased LPS-induced IL-1β levels in the cortex and hippocampus (**Figures 6A, B**) and LPS-mediated NLRP3 levels in the cortex but not the hippocampus (**Figures 6C, D**).

To assess the effects of varlitinib under acute neuroinflammation conditions, we pretreated wild-type mice with varlitinib and measured LPS-stimulated *il-1β* and *nlrp3* mRNA levels. Three-month-old C57BL/6 mice were injected with varlitinib (20 mg/kg, i.p.) or vehicle for 7 days. On the 7^th^ day, varlitinib (20 mg/kg, i.p.) or vehicle was injected, and 30 min later, LPS (10 mg/kg, i.p.) or PBS was administered. Eight hours after the last injection, the mice were sacrificed, and real-time PCR was conducted. Under these acute neuroinflammation conditions, pretreatment with varlitinib significantly decreased LPS-induced *il-1β* mRNA levels in the hippocampus only (**Figure 6E**) and *nlrp3* mRNA levels in the cortex only (**Figure 6F**). These observations imply that varlitinib suppresses LPS-mediated NLRP3 inflammasome activation and proinflammatory cytokine IL-1β expression in wild-type mice under acute and chronic neuroinflammation conditions.

### Varlitinib affects tau hyperphosphorylation and tau kinase DYRK1A levels in 3-month-old tau-overexpressing PS19 mice

**Figure 7:**
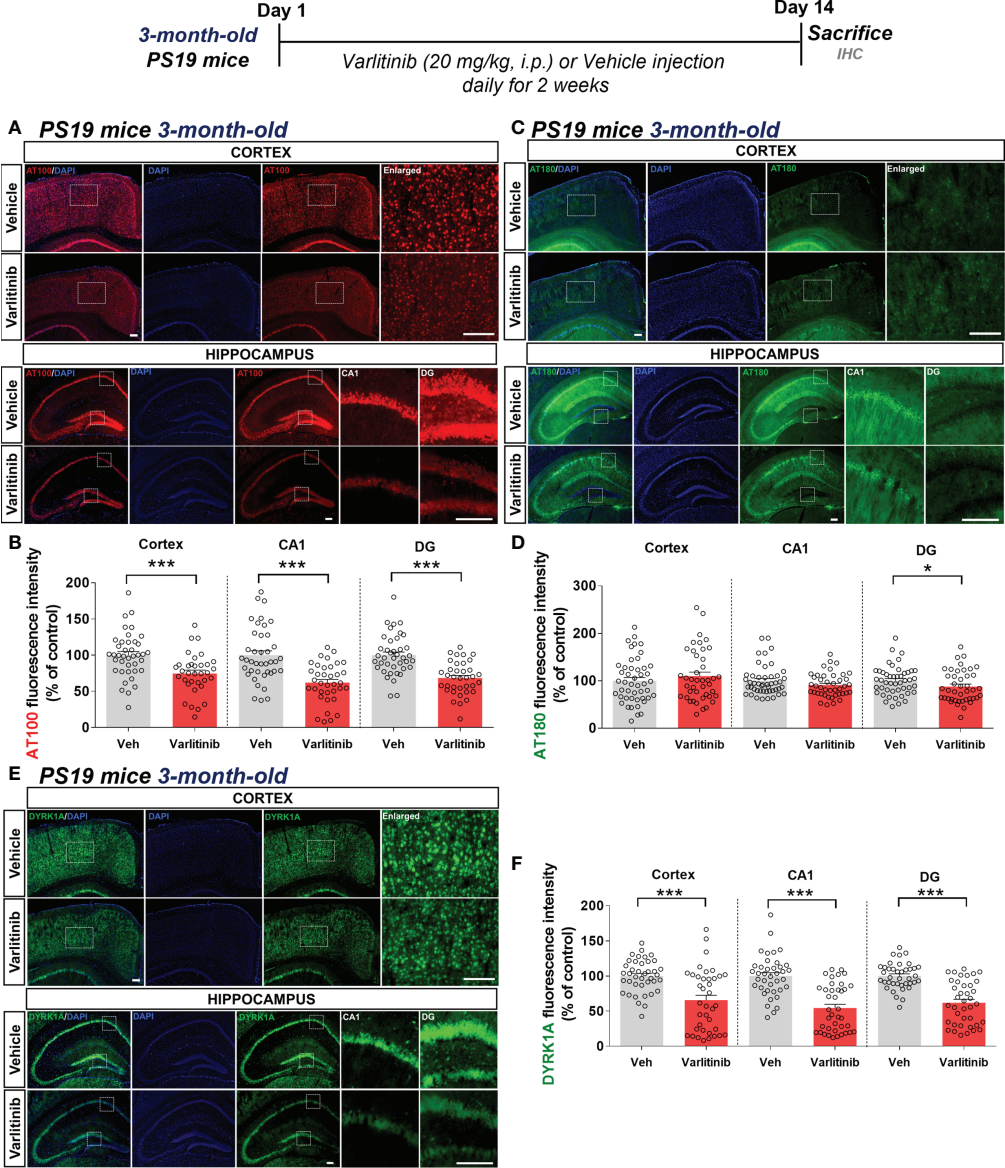
Varlitinib reduces tau hyperphosphorylation and tau kinase DYRK1A expression in 3-month-old tau-overexpressing PS19 mice. **(A, C, E)** Three-month-old tau-overexpressing PS19 mice were injected with varlitinib (20 mg/kg, i.p.) or vehicle (5% DMSO, 10% PEG, 20% Tween-80, i.p.) daily for 14 days, and immunofluorescence staining was conducted with anti-AT100, anti-AT180, and anti-DYRK1A antibodies. **(B, D, F)** Quantification of data from **(A, C, E)** (n = 11 mice/group, AT100: Veh: 40 brain sections, Varlitinib: 34 brain sections; AT180: Veh: 47 brain sections, Varlitinib: 42 brain sections; DYRK1A: Veh: 47 brain sections, Varlitinib: 42 brain sections, 2 experimental replicates per group). ^*^ *p* < 0.05, ^***^ *p* < 0.001. Veh: Vehicle. Scale bar=50 μm.

Since varlitinib downregulated gliosis in LPS-treated wild-type mice, we next examined the effects of varlitinib on AD pathology in a mouse model of AD. For this experiment, three-month-old tau-overexpressing PS19 mice were injected with varlitinib (20 mg/kg, i.p.) or vehicle (5% DMSO, 10% PEG, 20% Tween-80) daily for 2 weeks. Immunofluorescence staining with anti-AT100 (p-Tau^Thr212/Ser214^) or anti-AT180 (p-Tau^Thr231^) antibodies showed that varlitinib significantly inhibited tau phosphorylation at Thr212/Ser214 in the cortex and hippocampus (**Figures 7A, B**). In addition, varlitinib significantly decreased tau phosphorylation at Thr231 in the hippocampal DG region but not the cortex or hippocampal CA1 region (**Figures 7C, D**). These data indicate that varlitinib suppresses tau hyperphosphorylation in the early stage of tauopathy in tau-overexpressing PS19 mice.

Next, to examine the involvement of tau kinase activation in the effects of varlitinib on tau hyperphosphorylation in 3-month-old tau-overexpressing PS19 mice, we determined tau kinase levels by conducting immunofluorescence staining with anti-DYRK1A, anti-p-CDK5, or anti-p-GSK3β antibodies. Importantly, we found that varlitinib significantly downregulated the levels of the tau kinase DYRK1A in the cortex and hippocampus in 3-month-old tau-overexpressing PS19 mice (**Figures 7E, F**). By contrast, varlitinib had no effect on the levels of the tau kinases p-GSK3β and p-CDK5 in 3-month-old tau-overexpressing PS19 mice (**Supplementary Figures 1**, **2**). These data indicate that varlitinib selectively reduces tau kinase DYRK1A levels in the early stage of tauopathy in tau-overexpressing PS19 mice.

### The effects of varlitinib on tau hyperphosphorylation are reduced in 6-month-old tau-overexpressing PS19 mice

**Figure 8:**
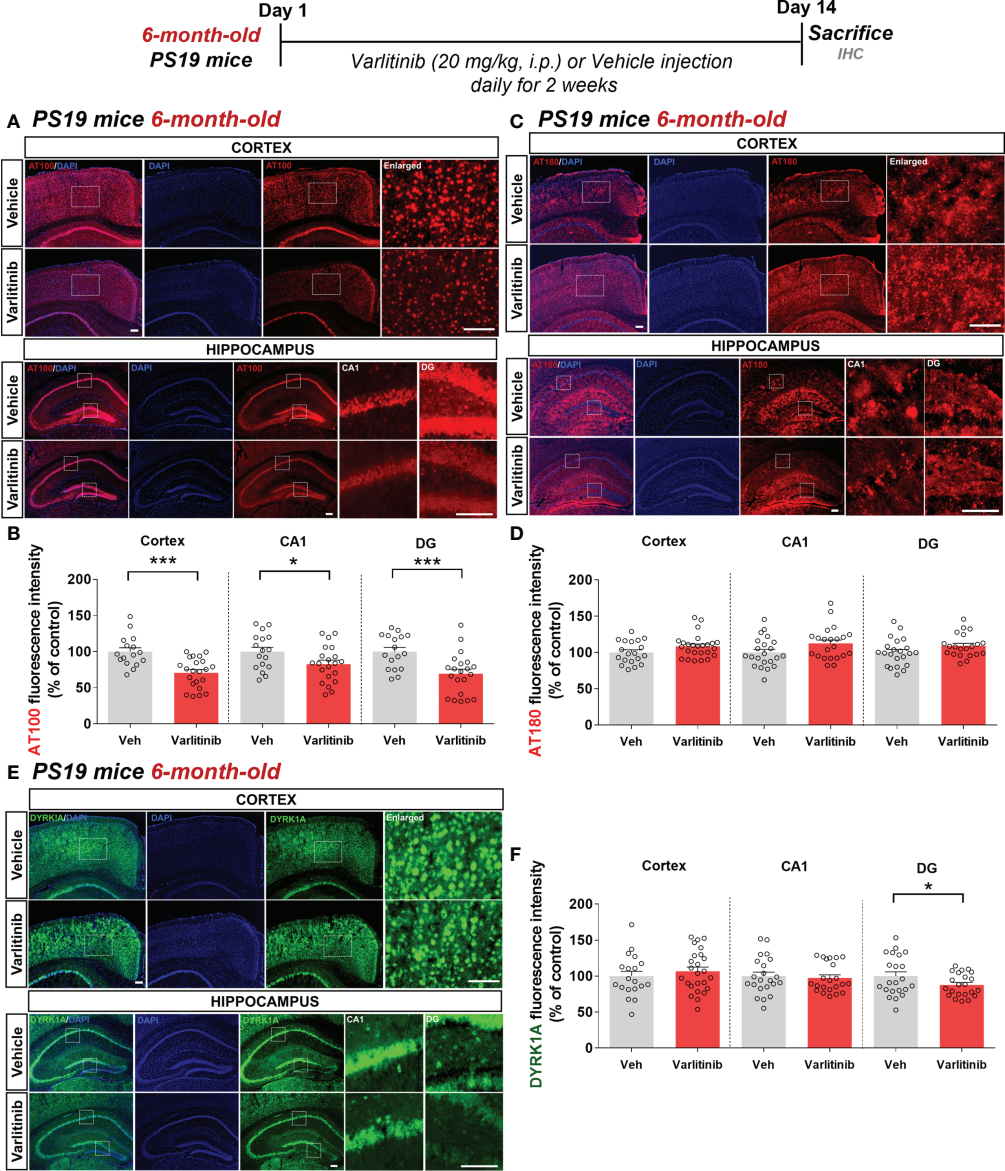
Varlitinib selectively diminishes tau phosphorylation in 6-month-old tau-overexpressing PS19 mice. **(A, C, E)** Six-month-old tau-overexpressing PS19 mice were injected with varlitinib (20 mg/kg, i.p.) or vehicle (5% DMSO, 10% PEG, 20% Tween-80, i.p.) daily for 14 days, and immunofluorescence staining was conducted with anti-AT100, anti-AT180, and anti-DYRK1A antibodies. **(B, D, F)** Quantification of data from **(A, C, E)** (n = 5 mice/group, AT100: Veh: 17 brain sections, Varlitinib: 21 brain sections; AT180: Veh: 17 brain sections, Varlitinib: 21 brain sections; DYRK1A: Veh: 17 brain sections, Varlitinib: 21 brain sections) ^*^ *p* < 0.05, ^***^ *p* < 0.001. Veh, Vehicle. Scale bar=50 μm.

To investigate whether varlitinib downregulates tau hyperphosphorylation in an age-dependent manner, 6-month-old PS19 mice were injected with varlitinib (20 mg/kg, i.p.) or vehicle (5% DMSO, 10% PEG, 20% Tween-80) daily for 2 weeks. Immunofluorescence staining with anti-AT100 or anti-AT180 antibodies showed that varlitinib significantly inhibited tau phosphorylation at Thr212/Ser214 in the cortex and hippocampus (**Figures 8A, B**) but did not affect tau phosphorylation at Thr231 in the cortex or hippocampus (**Figures 8C, D**).

In parallel with the experiments in 3-month-old mice, we determined the effects of varlitinib on tau kinase DYRK1A levels in 6-month-old tau-overexpressing PS19 mice treated as described above (**Figures 8E, F**). Interestingly, immunofluorescence staining with an anti-DYRK1A antibody showed that varlitinib significantly decreased DYRK1A levels in the hippocampal DG region but not in the cortex and hippocampal CA1 region (**Figures 8E, F**). These data indicate that varlitinib downregulates tau pathology and tau kinase DYRK1A levels more effectively in the early stage of tau overexpression than in aged PS19 mice.

### Varlitinib suppresses glial activation in 3-month-old tau-overexpressing PS19 mice

**Figure 9:**
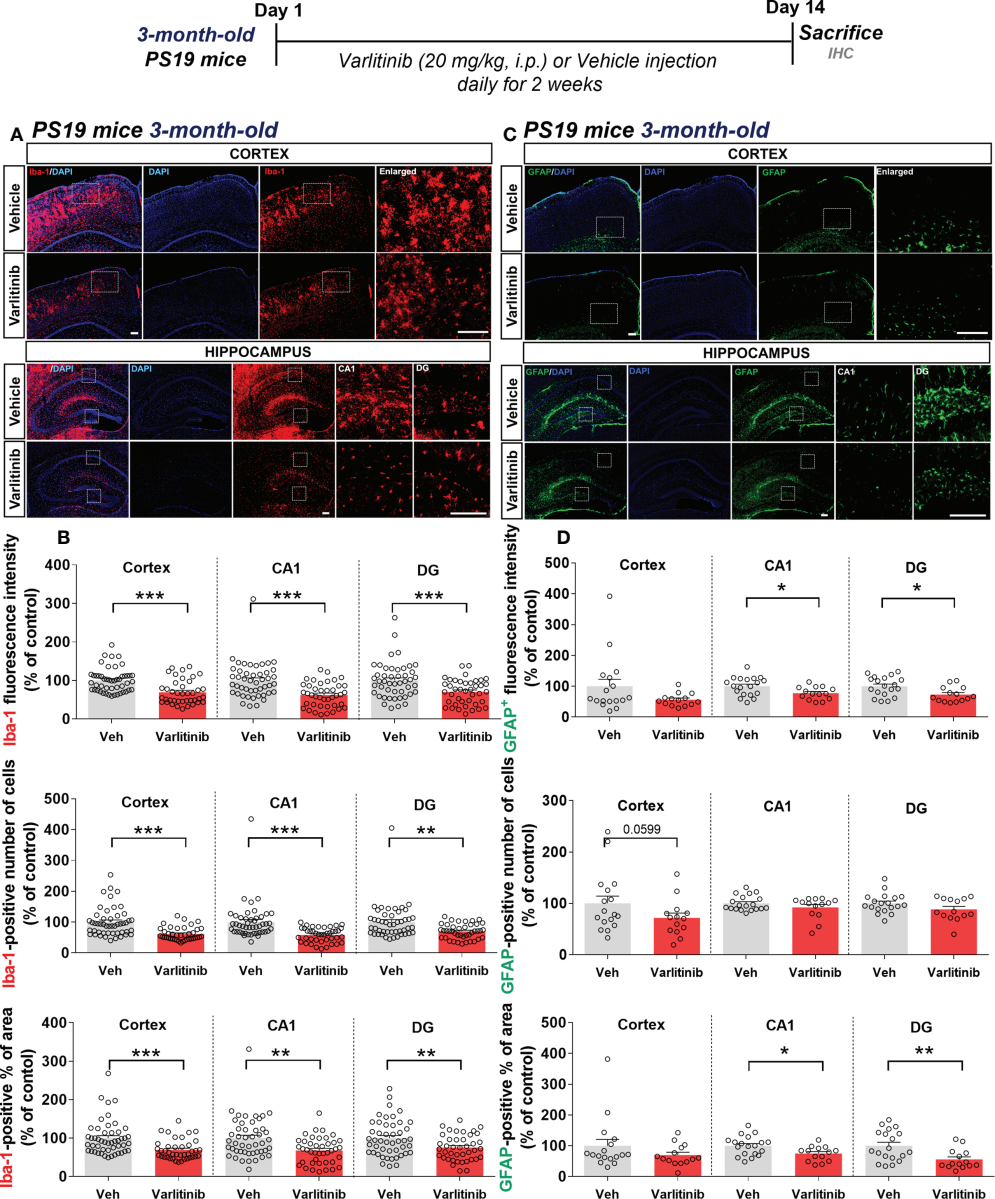
Varlitinib suppresses tau-mediated glial activation in 3-month-old tau-overexpressing PS19 mice. **(A, C)** Three-month-old tau-overexpressing PS19 mice were injected with varlitinib (20 mg/kg, i.p.) or vehicle (5% DMSO, 10% PEG, 20% Tween-80, i.p.) daily for 14 days, and immunofluorescence staining was carried out with anti-Iba-1 and anti-GFAP antibodies. **(B, D)** Quantification of data from A (n = 11 mice/group, Iba-1: Veh: 47 brain sections, Varlitinib: 40 brain sections, 2 experimental replicates per group) and C (n = 5 mice/group, GFAP: Veh: 17 brain sections, Varlitinib: 14 brain sections). ^*^ *p* < 0.05, ^**^ *p* < 0.01, ^***^ *p* < 0.001. Veh, Vehicle. Scale bar=50 μm.

Given the inhibitory effects of varlitinib on LPS-induced neuroinflammation in wild-type mice, we examined the impact of varlitinib on tau-induced neuroinflammation in the early stage of tauopathy. Three-month-old tau-overexpressing PS19 mice (Tau Tg PS19) were injected with varlitinib (20 mg/kg, i.p.) or vehicle (5% DMSO, 10% PEG, 20% Tween-80) daily for 2 weeks. Immunofluorescence staining with anti-Iba-1 and anti-GFAP antibodies showed that varlitinib significantly suppressed Iba-1 fluorescence intensity, the number of Iba-1-positive cells, and the percent Iba-1-positive area in the cortex and hippocampus (**Figures 9A, B**). In addition, GFAP immunofluorescence intensity and the percent GFAP-positive area were significantly reduced in the hippocampal CA1 and DG regions but not the cortex (**Figures 9C, D**). Moreover, varlitinib exhibited a trend toward decreasing the number of GFAP-positive cells in the cortex but not the hippocampus (**Figures 9C, D**). These data indicate that varlitinib suppresses tau-induced microglial and astrocyte activation in the early stage of tau overexpression.

Since varlitinib diminished LPS-induced proinflammatory responses by inhibiting NLRP3 inflammasome activation in wild-type mice, we investigated the effects of varlitinib on proinflammatory cytokine IL-1β levels and NLRP3 inflammasome formation in 3-month-old tau-overexpressing PS19 mice. Three-month-old tau-overexpressing PS19 mice were treated as described above, and immunofluorescence staining was conducted with anti-IL-1β or anti-NLRP3 antibodies. Unexpectedly, we observed that varlitinib did not alter IL-1β and NLRP3 levels in 3-month-old tau-overexpressing PS19 mice (**Supplementary Figure 3**).

### Varlitinib diminishes astrocyte activation in 6-month-old tau-overexpressing PS19 mice

**Figure 10:**
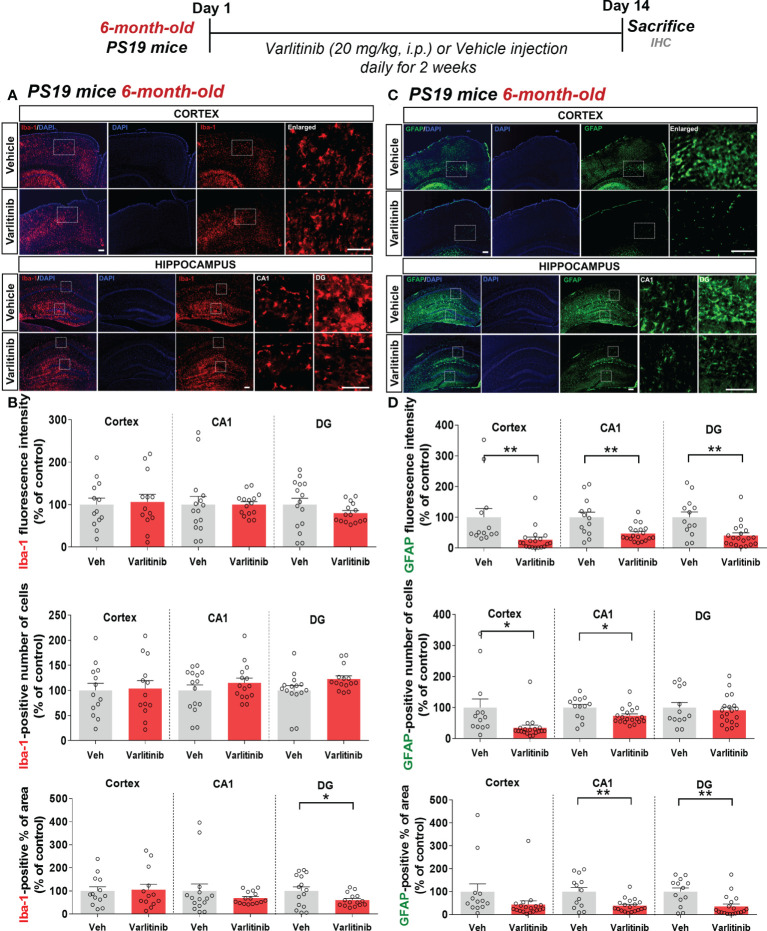
Varlitinib downregulates astroglial activation in 6-month-old tau-overexpressing PS19 mice. **(A, C)** Six-month-old tau-overexpressing PS19 mice were injected with varlitinib (20 mg/kg, i.p.) or vehicle (5% DMSO, 10% PEG, 20% Tween-80, i.p.) daily for 14 days, and immunofluorescence staining was conducted with anti-Iba-1 and anti-GFAP antibodies. **(B, D)** Quantification of data from **(A, C)** (n = 5 mice/group, Iba-1: Veh: 15 brain sections, Varlitinib: 15 brain sections; GFAP: Veh, 15 brain sections, Varlitinib: 15 brain sections). ^*^ *p* < 0.05, ^**^ *p* < 0.01. Veh: Vehicle. Scale bar=50 μm.

Varlitinib modulated gliosis in the early stage of tauopathy in 3-month-old PS19 mice; thus, we investigated the effects of varlitinib on microglial and astrocyte activation in older tau-overexpressing PS19 mice. Six-month-old PS19 mice were subjected to the same treatment regimen as 3-month-old mice. Immunofluorescence staining with anti-Iba-1 and anti-GFAP antibodies showed that varlitinib significantly reduced the percent Iba-1-positive area in the hippocampal DG region but not the cortex and hippocampal CA1 region (**Figures 10A, B**). Iba-1 fluorescence intensity and the number of Iba-1-positive cells in the cortex and hippocampus were not altered by varlitinib administration (**Figures 10A, B**). Interestingly, varlitinib significantly decreased GFAP fluorescence intensity in the cortex and hippocampus compared with vehicle in 6-month-old tau-overexpressing PS19 mice (**Figures 10C, D**). Moreover, varlitinib significantly suppressed the number of GFAP-positive cells in the cortex and hippocampal CA1 region but not in the hippocampal DG region (**Figures 10C, D**). Varlitinib significantly diminished the basal percent GFAP-positive area in the hippocampal CA1 and DG regions but not in the cortex (**Figures 10C, D**). These data suggest that the inhibitory effect of varlitinib on glial activation is reduced in 6-month-old tau-overexpressing PS19 mice compared with 3-month-old tau-overexpressing PS19 mice.

Additionally, we investigated whether varlitinib differentially modulates proinflammatory cytokine IL-1β and NLRP3 levels in 6-month-old tau-overexpressing PS19 mice treated as described. Immunofluorescence staining with anti-IL-1β or anti-NLRP3 antibodies demonstrated that varlitinib did not affect IL-1β and NLRP3 levels in 6-month-old tau-overexpressing PS19 mice (**Supplementary Figure 4**). These data suggest that varlitinib affects another neuroinflammation-associated molecular target (i.e., tau kinases) to regulate neuroinflammation in tau-overexpressing PS19 mice.

## Discussion

**Figure 11:**
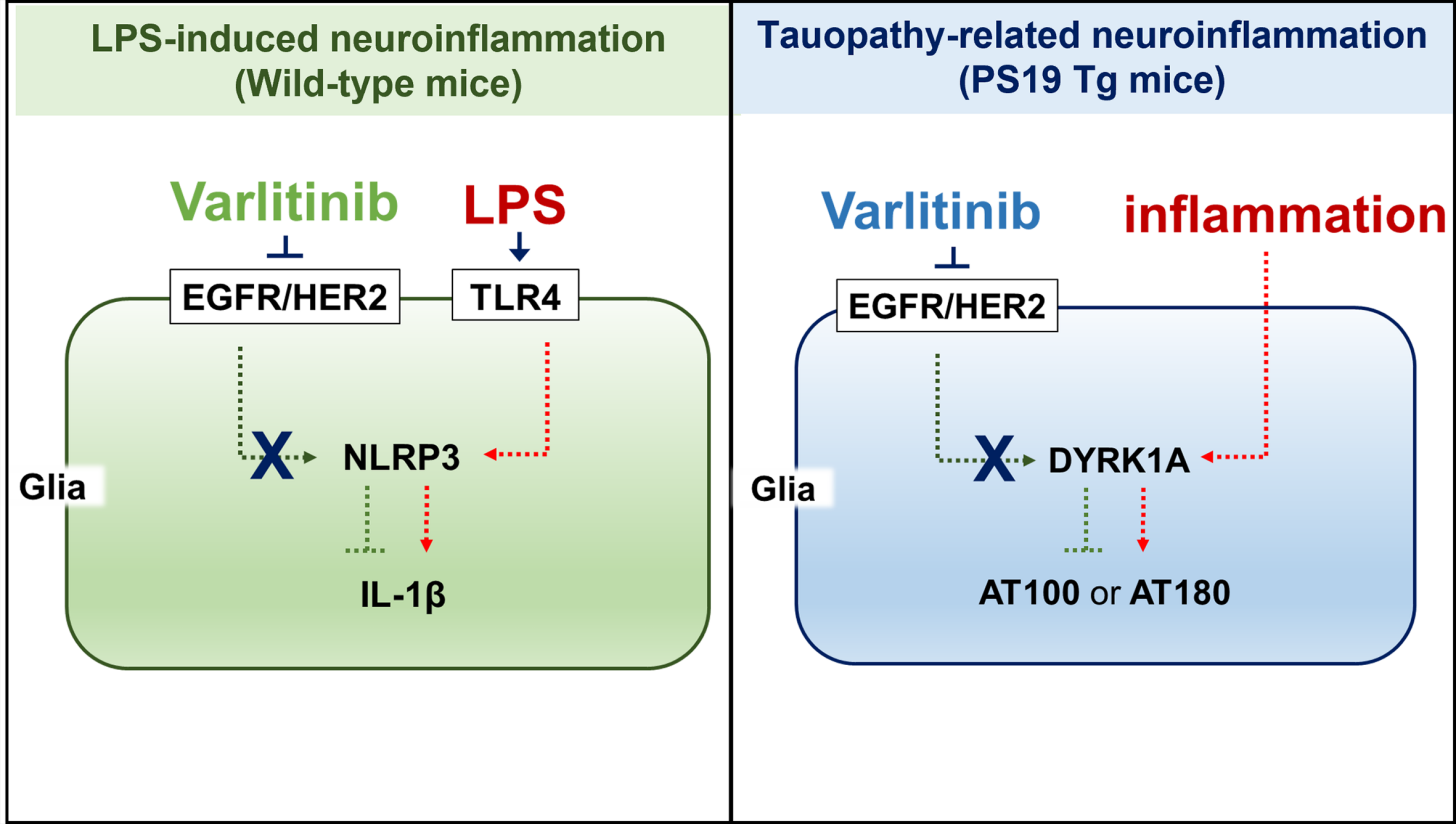
**(A)** working model for how varlitinib alters LPS/tau-mediated neuroinflammatory responses and tauopathy *in vivo*. In the LPS-induced model of neuroinflammation in wild-type mice, varlitinib inhibits EGFR/HER2 and TLR4-linked NLRP3 inflammasome activation, leading to suppression of LPS-induced proinflammatory/neuroinflammatory responses. In tau-overexpressing PS19 mice, varlitinib inhibits tau kinase DYRK1A activation, resulting in downregulation of neuroinflammatory responses and tau pathology.

Varlitinib, an EGFR/HER2 inhibitor and anti-breast cancer drug, is FDA approved for the treatment of breast cancer and is known to cross the blood–brain barrier (28). In the present study, we investigated the effects of varlitinib on LPS-evoked proinflammatory responses and found that varlitinib decreased LPS-induced proinflammatory/neuroinflammatory responses in BV2 microglial cells, primary astrocytes, and wild-type mice. We then investigated whether varlitinib modulates neuroinflammation and AD pathology in a mouse model of AD and found that varlitinib downregulated neuroinflammatory responses, tau hyperphosphorylation, and tau kinase DYRK1A expression in 3-month-old tau-overexpressing PS19 mice and, to a lesser extent, in 6-month-old tau-overexpressing PS19 mice (**Figure 11**). In summary, our findings imply that varlitinib is a potential therapy for reducing glial activation in the brain in response to neuroinflammation and the early stage of tauopathy.

EGFR is involved in the neuroinflammatory response, and EGFR inhibitors modulate proinflammatory responses related to LPS and/or AD pathology (12, 29, 30). For instance, the pharmacological EGFR inhibitors AG1478 and AG451 inhibit LPS-induced EGFR signaling and proinflammatory cytokine *il-1β*, *tnf-α*, *il-6* and *il-8* mRNA levels in human epithelial BEAS-2B cells (29). The EGFR inhibitor AG1478 attenuates LPS-induced chemokinetic migration of reactive primary microglia (31), and AG1478 and the EGFR inhibitor C225 significantly reduce the induction of *il-1β* and *tnf-α* mRNA expression in LPS-treated primary microglia and BV2 microglial cells (32). In addition, EGFR siRNA significantly reduces LPS-induced proinflammatory cytokine levels in BEAS-2B cells (29). We recently reported that the EGFR inhibitor regorafenib diminishes LPS-mediated proinflammatory cytokine levels in BV2 microglial cells (19). Moreover, we found that the tyrosine kinase inhibitor ibrutinib (on-target: BTK (Bruton’s tyrosine kinase); off-target: EGFR) downregulates LPS-induced proinflammatory cytokine *cox-2*, *il-1β*, *il-6*, *tnf-α* and *inos* mRNA levels in BV2 microglial cells (33). Thus, EGFR could be a useful target for treating neuroinflammation-associated neurodegenerative diseases. Here, we showed that pre- and post-treatment with the EGFR inhibitor varlitinib differentially affected LPS-induced proinflammatory cytokine and mediator levels (**Figure 1**); post-treatment regulated LPS-evoked proinflammatory cytokine levels more effectively than pretreatment in BV2 microglial cells (**Figure 1**). Together, the literature and our findings suggest that EGFR inhibitors differentially reduce LPS-mediated proinflammatory cytokine release by microglia *in vitro*.

TLR4 is a major member of the TLR family and plays an important role in the development of the innate immune response as a primary responder to LPS (34). TLR4 is activated by LPS, which leads to downstream signaling and proinflammatory cytokine release. We previously reported that the downregulation of *cox-2* and *il-1β* mRNA levels by the receptor tyrosine kinase (RTK) and EGFR inhibitor regorafenib in BV2 microglia is partially dependent on TLR4 signaling (19). In addition, the BTK/EGFR inhibitor ibrutinib suppresses LPS-induced *il-1β* but not *cox-2* mRNA levels in a TLR4-dependent manner in BV2 microglia (33). This study is the first to examine the role of TLR4 signaling in the effects of the EGFR inhibitor varlitinib on LPS-mediated proinflammatory cytokine levels. We confirmed that the impact of varlitinib on LPS-stimulated proinflammatory responses is partially dependent on TLR4 signaling in microglia (**Figure 2**). Our findings suggest that varlitinib downregulates neuroinflammatory-associated downstream signaling factors/transcription factors/molecular targets to ameliorate LPS-mediated proinflammatory responses.

TLR4 signaling is closely mediated by the MAPK/AKT and/or FAK signaling pathways in microglia (35). Several studies have shown that EGFR increases macrophage proinflammatory cytokine gene expression by activating downstream AKT and FAK signaling (36–39). For instance, the recently reported EGFR inhibitor FAG from *Ficus benghalensis* significantly diminishes LPS-induced proinflammatory responses *via* PI3K-AKT signaling in RAW 264.7 macrophage cells (40). Another EGFR inhibitor, AG1478, decreases morphine-promoted EGFR/FAK signaling in BV2 microglial cells (41). The EGFR inhibitor gefitinib reduces AKT phosphorylation in an ovalbumin-induced inflammation model (42). We previously found that the BTK/EGFR or RTK/EGFR inhibitors ibrutinib and regorafenib diminish the LPS-stimulated activation of AKT but not p-JNK and p-P38 in BV2 microglial cells (19, 33). Here, we found that varlitinib significantly decreased LPS-induced AKT and FAK phosphorylation in BV2 microglia (**Figure 2**). Thus, our findings and previous work indicate that EGFR inhibition suppresses LPS-stimulated proinflammatory cytokine expression by modulating AKT and FAK activation in microglia.

The transcription of proinflammatory cytokines in microglia is associated with the activity of the transcription factor NF-κB (43). The EGFR inhibitor gefitinib reduces nuclear NF-κB phosphorylation in an ovalbumin-mediated mouse inflammation model (42). Knockdown of the EGFR subunit ErbB4 abrogates NF-κB activation in reactive M2 microglia (44). The induction of proinflammatory cytokines by *Escherichia coli* is downregulated by the EGFR-NF-kB signaling pathway *in vitro* (45). In our previous study, we found that ibrutinib and regorafenib reduce LPS-induced nuclear STAT3 activation in BV2 microglial cells (19, 33). Here, we found that the EGFR inhibitor varlitinib downregulated LPS-mediated NF-kB levels in the nucleus in BV2 microglial cells (**Figure 3**). This body of evidence suggests that EGFR inhibition modulates the AKT/FAK and NF-kB/STAT3 signaling pathways to regulate the neuroinflammatory response in microglial cells.

The inflammasome is a multi-protein system that activates pro-caspase-1, IL-18 and IL-1β (46). NOD-, LRR-domain-containing protein (NLR) family proteins are crucial members of the inflammasome and have been linked to innate and adaptive immune system regulation, inflammation, and autoimmunity (47). The NLR family member NLRP3 regulates the expression of the proinflammatory cytokine IL-1β. Increased or prolonged NLRP3 inflammasome activation is associated with the development of a variety of neuroinflammation-related disorders, including AD (48). In a mouse model of lung inflammation, particulate matter 2.5 (PM2.5) significantly increases *il-1β* mRNA levels *via* EGFR/NLRP3 activation (49), suggesting that EGFR signaling and NLRP3 are involved in the inflammatory response (50). Moreover, the BTK and EGFR inhibitor ibrutinib significantly suppresses IL-1β protein levels by downregulating NLRP3 expression in macrophages (51). When *nlrp3* is knocked down in human primary keratinocytes, EGFR stimulation no longer increases *il-1β* expression (52). In this study, the EGFR inhibitor varlitinib significantly inhibited LPS-mediated *nlrp3* and *pro-il-1β* mRNA expression, and varlitinib did not alter LPS-induced *il-1β* mRNA levels in BV2 microglial cells when *nlrp3* was knocked down (**Figure 3**). These findings suggest that inhibiting EGFR alters LPS-induced proinflammatory responses by inhibiting the formation of the NLRP3 inflammasome in microglia. Of course, it is possible that EGFR inhibition impacts other neuroinflammation-associated molecular targets (e.g., CDK6, DYRK1A) to influence LPS-evoked proinflammatory responses. Future studies will address this issue and determine whether EGFR knockdown or overexpression directly downregulates NLRP3 inflammasome activation.

Neuroinflammatory responses in the CNS are also regulated by astrocytes (53), which are activated by extracellular pathogens or molecules (e.g., LPS) or activated microglia (53). Activated astrocytes release chemokines and cytokines *via* stimulation of AKT signaling or the NLRP3 inflammasome (53–55). Several recent studies have demonstrated that EGFR inhibitors modulate LPS-mediated proinflammatory responses through AKT/MAPK signaling in primary astrocytes (19, 56). For example, the EGFR-TKI inhibitor afatinib significantly reduces oxygen-glucose deprivation-induced *cox-2* and *il-1β* mRNA levels and NO levels by suppressing AKT phosphorylation in primary astrocytes (56). The TKI regorafenib reduces proinflammatory cytokine levels by deactivating AKT signaling in primary astrocytes (19). Inhibitor of differentiation 3 (ID3)-induced increases in IL-6 and IL-8 are significantly decreased by treatment with an EGFR inhibitor that directly dephosphorylates AKT in glioma cells (57). In the present study, varlitinib significantly downregulated proinflammatory cytokine release and AKT phosphorylation in primary astrocytes (**Figure 4**). Taken together, these data suggest that inhibition of EGFR is required to suppress neuroinflammatory responses and that EGFR-linked AKT signaling contributes to the reduction of proinflammatory cytokine levels in astrocytes.

Because there is a clear association between EGFR and NLRP3 in cancer cells (58, 59), we examined EGFR-associated NLRP3 inflammasome activation in astrocytes in this study. Interestingly, varlitinib reduced LPS-stimulated *nlrp3* and *il-1β* mRNA levels in primary astrocytes (**Figure 4**). Our results are supported by Zhu et al.’s report that the EGFR inhibitor UNC9995 inhibits LPS-induced astrocytic NLRP3 inflammasome and IL-1β levels in a mouse model of Parkinson’s disease (PD) (60). These observations indicate that inhibition of EGFR in astrocytes may reduce proinflammatory cytokine release *via* AKT signaling and/or the NRLP3 inflammasome. However, we recognize that the inhibition of EGFR by varlitinib may alter LPS-mediated MAPK signaling (in addition to AKT signaling) and other neuroinflammation-associated molecular targets in primary astrocytes. Future studies will address this issue.

LPS induces both microglial and astrocyte activation *in vivo*, but acute and chronic LPS treatment differentially affect glial activation and morphology. Under acute neuroinflammation conditions *in vivo*, LPS activates glial cells, which migrate to the lesion and secrete growth factors (e.g., GDNF, NGF) and pro- and anti-inflammatory cytokines (61). Under chronic neuroinflammation conditions, microglia become highly activated, as indicated by swelling, and reactive microglia and astrocytes secrete proinflammatory cytokines and increase the cell death rate of neurons (61). EGFR inhibition has been shown to regulate microglial and astrocyte activities and neuroinflammatory responses *in vivo* (19, 33). For example, we found that the RTK/EGFR inhibitor regorafenib significantly reduces LPS-induced microglial activation but is less effective against astrocyte activation in wild-type mice under acute neuroinflammation conditions (19). In addition, we previously observed that daily injection of the BTK/EGFR inhibitor ibrutinib for 3 days suppresses LPS-stimulated micro- and astroglial activation in wild-type mice under acute neuroinflammation conditions (33). Another EGFR inhibitor, erlotinib, decreases microglial and astrocyte activation in LPS-treated mice (62). The present study is the first to show that the EGFR inhibitor varlitinib can significantly reduce LPS-evoked microglia/astrocyte activation and morphology in wild-type mice (**Figure 5**), which indicates that EGFR inhibition has the potential to suppress glial activation *in vivo*. Varlitinib significantly reduced IL-1β and NLRP3 expression in wild-type mice under both chronic and acute LPS-induced neuroinflammation (**Figure 6**). Exposure to PM2.5 significantly increases EGFR and NLRP3 expression in a mouse model of cardiac inflammation (63). In addition, in a mouse model of lung inflammation, EGFR signaling and the NLRP inflammasome directly increase IL-1β levels (49). In summary, our results and previous observations suggest that varlitinib modulates the neuroinflammatory response in the brains of LPS-treated wild-type mice by inhibiting the NLRP3 inflammasome.

Hyperphosphorylation of tau and aggregation of neurofibrillary tangles (NFTs) activate chronic microglial and astrocyte activation and vice versa (9, 64). In tau-overexpressing PS19 mice, phospho-tau pathology begins in the entorhinal cortex and hippocampal DG at 2 months of age (65). Yin et al. reported that DYRK1A overexpression leads to increased expression of three microtubule binding repeat tau (3R-Tau) and cognitive deficits in Ts65Dn mice (66). In addition, tau phosphorylation at Thr212 in the hippocampus is significantly higher in human DYRK1A-overexpressing transgenic mice than in wild-type mice (67). Interestingly, we previously found that the TKI/EGFR inhibitor ibrutinib significantly downregulates tau phosphorylation at Ser202/Thr205 (AT8) and Thr212/Ser214 (AT100) and tau kinase p-CDK5 levels in 3-month-old tau-overexpressing PS19 mice (12). However, the effects of EGFR inhibition on tau pathology in this mouse model of AD remain unclear. Thus, we assessed whether varlitinib can reduce tauopathy and found that varlitinib significantly decreased tau phosphorylation at Thr212/Ser214 and Thr231 (AT180) as well as tau kinase DYRK1A levels in 3-month-old PS19 mice (**Figure 7**). However, in 6-month-old PS19 mice, varlitinib significantly reduced tau phosphorylation at Thr212/Ser214 but not Thr231 (**Figure 8**). These divergent findings indicate that varlitinib suppresses tau phosphorylation more effectively in the early stage of tau pathology than in the middle phase of tauopathy. However, it is possible that a longer treatment period (e.g., 1 month) or a higher dose of varlitinib might differentially regulate tau phosphorylation and tau kinase activity in aged tau-overexpressing PS19 mice. Future studies will compare the effects of EGFR inhibitors on tauopathy in young and aged mice.

Neuroinflammation is a common feature of neurodegenerative diseases, including AD. Glial activation, which is crucial for regulating neuroinflammatory responses, is induced in the brain in AD (12, 68, 69) and may play a role in AD pathology (e.g., Aβ aggregation, tau fibrils). Accordingly, regulating glial activity should be a good target for alleviating neuroinflammation-associated diseases such as AD. Several recent studies have focused on EGFR and/or EGFR inhibitors for the treatment of AD because of the contribution of EGFR to AD pathology and neuroinflammation. The EGFR agonist neuregulin-1 (NRG-1) is highly co-expressed with the EGFR subunit ErbB4 and microglia/astrocytes in the hippocampus in AD patients and APP_K670N;M671L_/PS1_M146L_ mutant mice (68). Astrogenesis is absent in ErbB4 knockout mice even when NRG-1 is administered to the developing brain (69). Additionally, the TKI/EGFR inhibitor ibrutinib effectively ameliorates the neuroinflammatory response by reducing microglial and astrocyte activation in 5xFAD mice and/or tau-overexpressing PS19 mice (12). Consistent with these findings, varlitinib downregulated neuroinflammation by altering glial inactivation in 3-month-old PS19 mice (**Figure 9**) in the present study. Unexpectedly, varlitinib reduced astroglial activation but was less effective against microglial activation in 6-month-old PS19 mice (**Figure 10**). How does varlitinib differentially regulate microglial and/or astrocyte activity in the early stage (3 months old) vs middle stage (6 months old) of tauopathy in tau-overexpressing PS19 mice? It has been suggested that the role and expression of EGFR are differentially regulated in a disease-dependent manner. For example, the expression of EGF and EGFR is reduced in the brains of PD patients and does not repair the parkin protein mutation (70). Aged APP/PS1 transgenic mice, a model of AD, exhibit significantly lower EGFR expression than mice in the early stage of AD, resulting in increased Aβ plaques, memory loss, and neurodegeneration (70). However, no research has investigated the possible role of EGFR in gliosis in aged tau-overexpressing mice compared with mice in the early stage of tau pathology. Based on the literature, we speculate that malfunction or disruption of EGFR homeostasis occurs in the brains of aged PS19 mice. To address this hypothesis, we will verify the different roles of EGFR and EGFR inhibitors in the early and middle phases of gliosis and the mechanisms by which EGFR inhibitors modulate tauopathy in a later study.

In this study, we unexpectedly found that varlitinib did not regulate IL-1β expression and NLRP3 inflammasome formation in 3- and 6-month-old tau-overexpressing PS19 mice (**Supplementary Figures 1**, **2**). It is possible that varlitinib regulates other neuroinflammation-related molecular targets (e.g., DYRK1A, which is reduced in varlitinib-treated tau-overexpressing PS19 mice and is an important molecule for neuroinflammation) to alter tauopathy-induced neuroinflammation, which will be investigated in future studies. Overall, our work and previous observations imply that EGFR inhibition may differentially downregulate LPS-induced microglial and astroglial activity in the early and middle phases of tau pathology.

## Conclusion

Varlitinib suppresses LPS-mediated proinflammatory cytokine release and AKT and/or FAK/NF-kB signaling in BV2 microglial cells and primary astrocytes. Importantly, varlitinib significantly suppresses LPS-induced NLRP3 inflammasome formation in BV2 microglial and primary astrocytes. In a mouse model of neuroinflammation induced by LPS, varlitinib reduces microglial and astrocyte activation as well as IL-1β/NLRP3 levels. Moreover, varlitinib more effectively inhibits tau-induced gliosis, tau phosphorylation and tau kinase DYRK1A expression in the early stage of tauopathy than in the middle stage of tauopathy in tau-overexpressing PS19 mice. In summary, our results suggest that varlitinib can protect the brain from neuroinflammation and may have therapeutic effects on tau-mediated neuroinflammatory responses and tau pathology.

## Data availability statement

The original contributions presented in the study are included in the article/**Supplementary Material**. Further inquiries can be directed to the corresponding authors.

## Ethics statement

The animal study was reviewed and approved by the institutional biosafety committee (IBC) and performed in accordance with approved animal protocols of the Korea Brain Research Institute (KBRI, approval nos. IACUC-19-00042 and IACUC-19-00049).

## Author contributions

Study conception and design: MM and H-SH. Acquisition of data: JK, S-JK, H-RJ, and J-HP. Preparation of all figures: JK and S-JK. Preparation of tables: JK Writing of manuscript: JK, S-JK, and H-SH. Revision and replication experiments: JK. Writing of revised manuscript: JK and H-SH. All authors contributed to the article and approved the submitted version.

## Funding

This work was supported by the KBRI basic research program through KBRI funded by the Ministry of Science, ICT & Future Planning (grant numbers 22-BR-02-03, 22-BR-03-05, and 22-BR-04-01, H-SH), the National Research Foundation of Korea (grant numbers 2019R1A2B5B01070108 H-SH, and 2021R1F1A1057865, JK), and the Korea Health Technology R&D Project through the Korea Health Industry Development Institute (KHIDI) funded by the Ministry of Health & Welfare, Republic of Korea (grant number HF21C0021, MM). This work was supported by a National Research Council of Science & Technology (NST) grant funded by the Korean government [CCL22061-100, H-SH].

## Acknowledgments

Confocal microscopy (Nikon, TI-RCP) data were acquired at the Advanced Neural Imaging Center at the Korea Brain Research Institute (KBRI). We thank previous and current members of the neurodegenerative diseases lab for editing and valuable comments on our manuscript and fortechnical assistance with *in vitro* work and *in vivo* studies.

## Conflict of interest

The authors declare that the research was conducted in the absence of any commercial or financial relationships that could be construed as a potential conflict of interest.

## Publisher’s note

All claims expressed in this article are solely those of the authors and do not necessarily represent those of their affiliated organizations, or those of the publisher, the editors and the reviewers. Any product that may be evaluated in this article, or claim that may be made by its manufacturer, is not guaranteed or endorsed by the publisher.

## Abbreviations

TNBC, triple-negative breast cancer; CNS, central nervous system; LPS, lipopolysaccharide; AD, Alzheimer’s disease; Aβ, amyloid β; NFTs, neurofibrillary tangles; PD, Parkinson’s disease; DYRK1A, dual specificity tyrosine phosphorylation regulated kinase 1A; TLR4, toll-like receptor 4; COX-2, cyclooxygenase-2; NF-kB, nuclear factor kappa B; RTK, receptor tyrosine kinase; BTK, Bruton’s tyrosine kinase; EGFR, epidermal growth factor receptor.
