# Supplementary material for: Inhibiting EGFR/HER-2 ameliorates neuroinflammatory responses and the early stage of tau pathology through DYRK1A
Source: Front Immunol. 2022 Oct 20;13:903309. doi: 10.3389/fimmu.2022.903309 (PMC9632417; doi:10.3389/fimmu.2022.903309)
Supplement: Supplementary file 1 [file DataSheet_1.docx]

# Inhibiting EGFR/HER-2 ameliorates neuroinflammatory responses and the early stage of tau pathology through DYRK1A

Jieun Kim^1,4^, Su-Jin Kim^3,4^, Ha-Ram Jeong^1^, Jin-Hee Park^1,2^, Minho Moon^3,*^, Hyang-Sook Hoe^1,2,*^

^1^Department of Neural Development and Disease, Korea Brain Research Institute (KBRI), 61, Cheomdan-ro, Dong-gu, Daegu, Korea. 41068; ^2^Department of Brain & Cognitive Sciences, Daegu Gyeongbuk Institute of Science & Technology (DGIST), Daegu, Korea, 42988; ^3^Department of Biochemistry, College of Medicine, Konyang University, Daejeon, 35365, Korea; ^4^These authors contributed equally to this work.

*Corresponding author

Hyang-Sook Hoe, Ph.D.: Department of Neural Development and Disease, Korea Brain Research Institute (KBRI), 61 Cheomdan-ro, Dong-gu, Daegu, Korea, 41068

E-mail: [sookhoe72@kbri.re.kr](mailto:sookhoe72@kbri.re.kr)

Minho Moon, Ph.D.: Department of Biochemistry, College of Medicine, Konyang University, Daejeon, Korea, 35365

E-mail: [hominmoon@konyang.ac.kr](mailto:hominmoon@konyang.ac.kr)

**
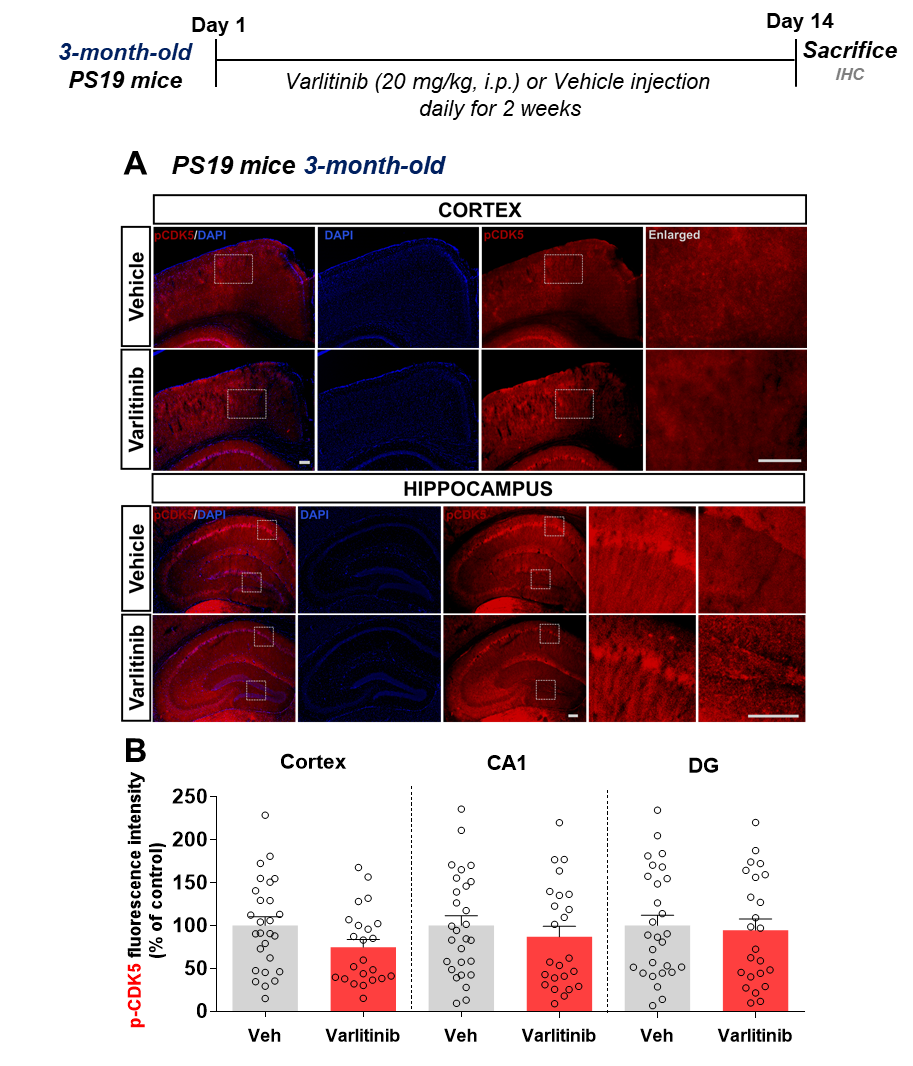
**

**Supplementary Figure 1**. Varlitinib does not affect p-CDK5 levels in 3-month-old tau-overexpressing Tau Tg PS19 mice. (A) Immunostaining of p-CDK5 expression in 3-month-old Tau Tg PS19 mice injected (i.p.) with varlitinib (20 mg/kg) or vehicle (5% DMSO, 10% PEG, 20% Tween-80) daily for 14 days. (B) Quantification of data from A (n = 6 mice/group, Veh: 27 brain sections; Varlitinib: 23 brain sections). Veh: Vehicle. Scale bar=50 μm.

**
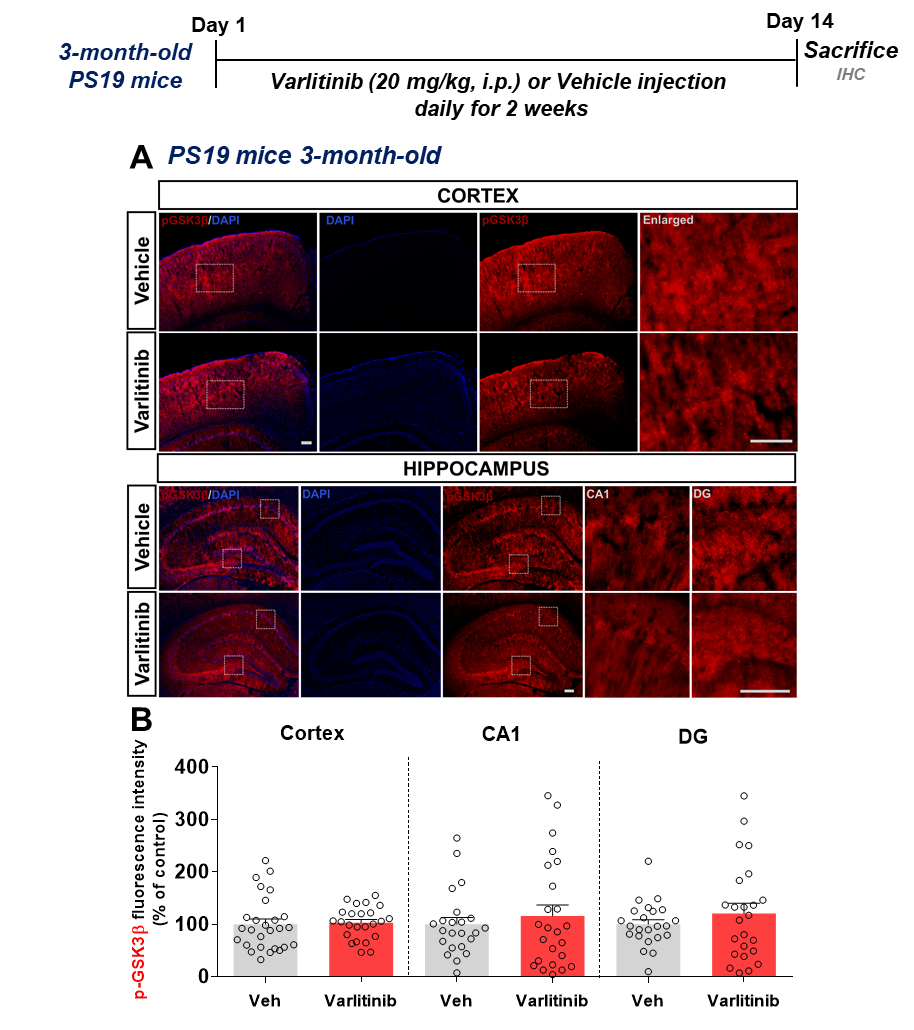
**

**Supplementary Figure 2**. Varlitinib does not modulate p-GSK3β levels in 3-month-old tau-overexpressing mice. (A) Immunostaining of p-GSK3β expression in 3-month-old Tau Tg PS19 mice injected (i.p.) with varlitinib (20 mg/kg) or vehicle (5% DMSO, 10% PEG, 20% Tween-80) daily for 14 days. (B) Quantification of data from A (n = 6 mice/group, Veh: 23 brain sections; Varlitinib: 24 brain sections). Veh: Vehicle. Scale bar=50 μm.

**
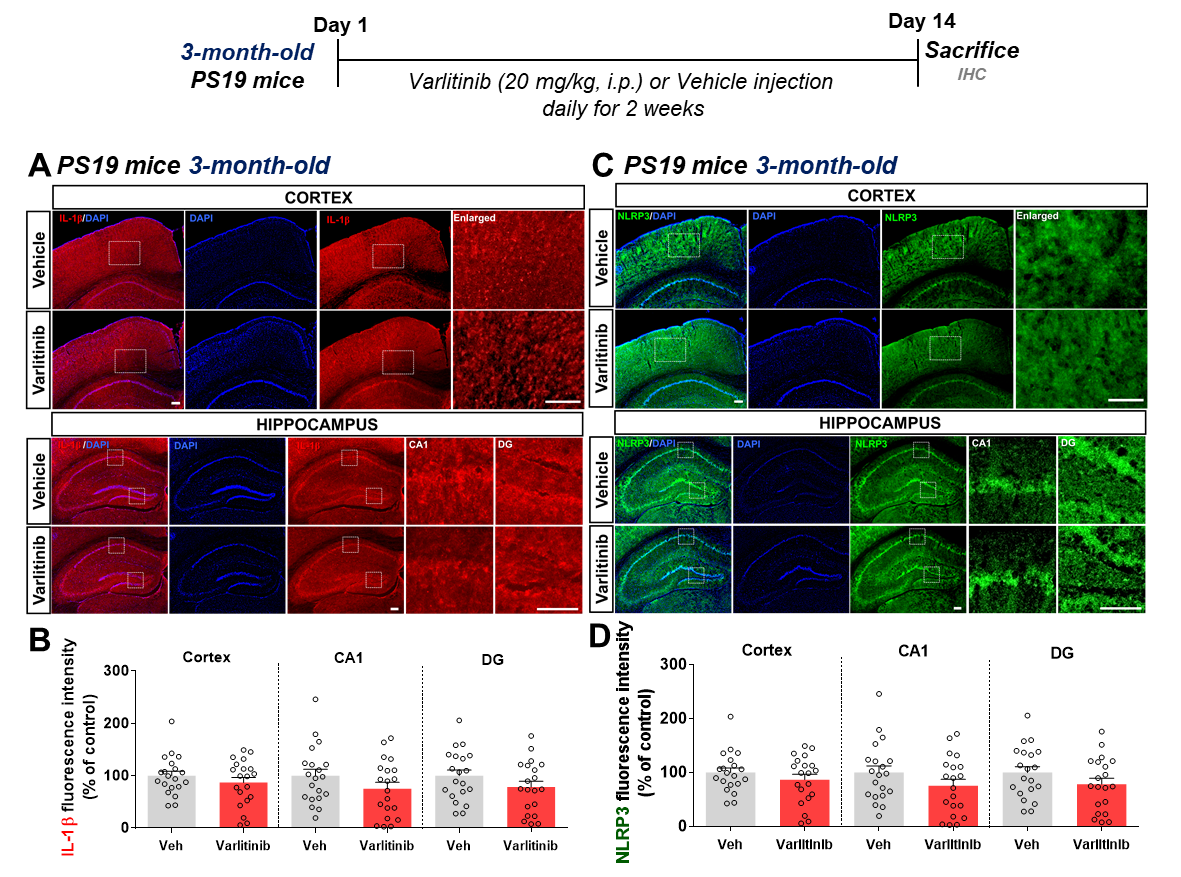
**

**Supplementary Figure 3**. Varlitinib does not alter IL-1β and NLRP3 levels in 3-month-old tau-overexpressing PS19 mice (Tau Tg PS19). (A) Immunostaining of IL-1β expression in 3-month-old Tau Tg PS19 mice injected (i.p.) with varlitinib (20 mg/kg) or vehicle (5% DMSO, 10% PEG, 20% Tween-80) daily for 14 days. (B) Quantification of data from A (Veh: n = 6 mice/group, 20 brain sections/group). (C) Immunostaining of NLRP3 expression in 3-month-old Tau Tg PS19 mice injected with varlitinib (20 mg/kg) or vehicle (5% DMSO, 10% PEG, 20% Tween-80) daily for 14 days. (D) Quantification of data from C (Veh: n = 6 mice/group, 20 brain sections/group). Veh: Vehicle. Scale bar=50 μm.

**
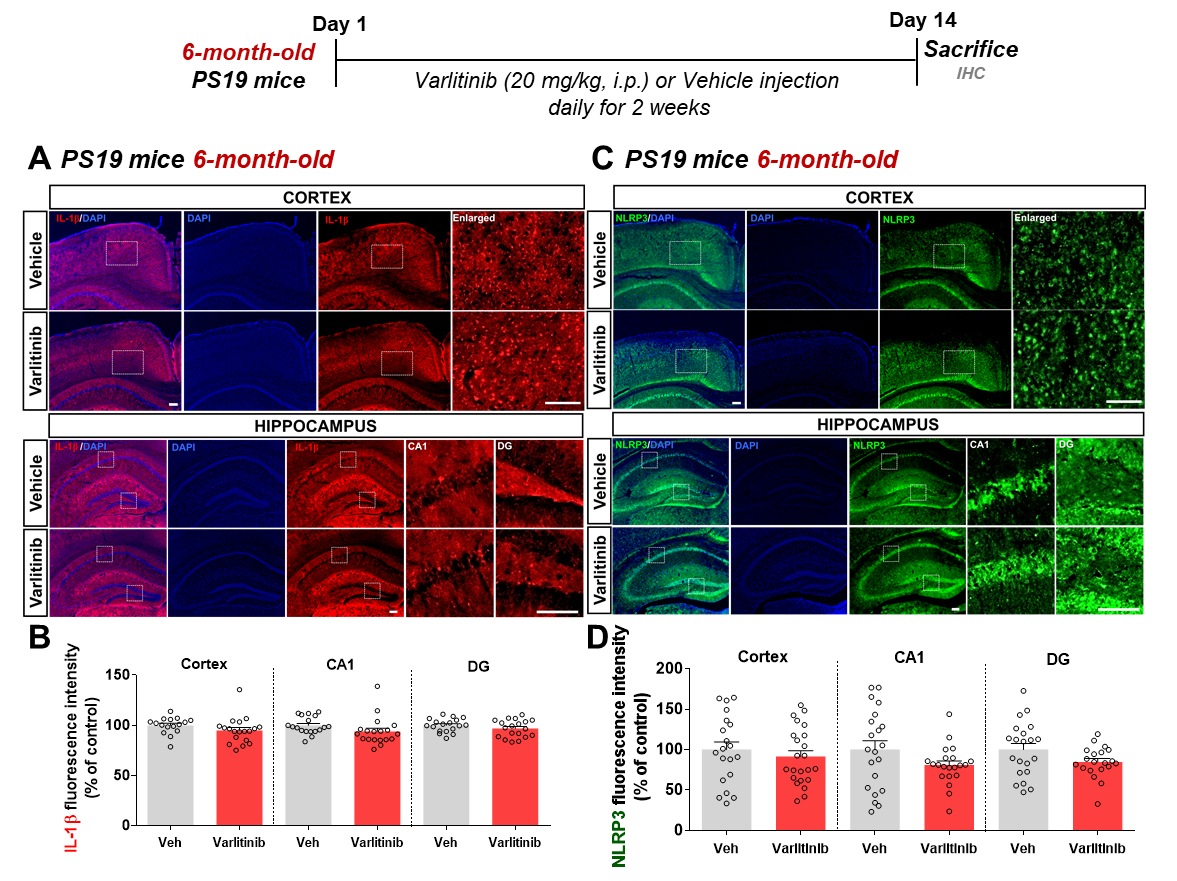
**

**Supplementary Figure 4**. Varlitinib does not affect IL-1β and NLRP3 levels in 6-month-old tau-overexpressing mice. (A) Immunostaining of IL-1β expression in 6-month-old tau-overexpressing PS19 (Tau Tg PS19) mice injected with varlitinib (20 mg/kg) or vehicle (5% DMSO, 10% PEG, 20% Tween-80) daily for 14 days. (B) Quantification of data from A (n=5 mice/group, Veh: 18 brain sections; Varlitinib: 19 brain sections). (C) Immunostaining of NLRP3 expression in 6-month-old Tau Tg PS19 mice injected (i.p.) with varlitinib (20 mg/kg) or vehicle (5% DMSO, 10% PEG, 20% Tween-80) daily for 14 days. (D) Quantification of data from C. (n=5 mice/group, Veh: 18 brain sections; Varlitinib: 19 brain sections). Veh: Vehicle. Scale bar=50 μm.

**Supplementary table 1**. One-way ANOVA (Tukey’s test) and significance of the results of the *in vitro* experiments in this study.

| **Figure 1B MTT** | |
| --- | --- |
| \| Number of families \| 1 \|  \|  \|  \|  \|  \| \| --- \| --- \| --- \| --- \| --- \| --- \| --- \| \| Number of comparisons per family \| 66 \|  \|  \|  \|  \|  \| \| Alpha \| 0.05 \|  \|  \|  \|  \|  \| \|  \|  \|  \|  \|  \|  \|  \| \| Tukey's multiple comparisons test \| Mean Diff. \| 95.00% CI of diff. \| Significant? \| Summary \| Adjusted P Value \|  \| \|  \|  \|  \|  \|  \|  \|  \| \| Column A vs. Column B \| -0.652 \| -8.492 to 7.188 \| No \| ns \| >0.9999 \| A-B \| \| Column A vs. Column C \| 1.187e-005 \| -7.84 to 7.84 \| No \| ns \| >0.9999 \| A-C \| \| Column A vs. Column D \| -0.6576 \| -8.498 to 7.183 \| No \| ns \| >0.9999 \| A-D \| \| Column A vs. Column E \| 7.5e-006 \| -7.84 to 7.84 \| No \| ns \| >0.9999 \| A-E \| \| Column A vs. Column F \| -5.03 \| -12.87 to 2.81 \| No \| ns \| 0.6060 \| A-F \| \| Column A vs. Column G \| 8.125e-006 \| -7.84 to 7.84 \| No \| ns \| >0.9999 \| A-G \| \| Column A vs. Column H \| -2.433 \| -10.27 to 5.407 \| No \| ns \| 0.9969 \| A-H \| \| Column A vs. Column I \| 4.375e-006 \| -7.84 to 7.84 \| No \| ns \| >0.9999 \| A-I \| \| Column A vs. Column J \| 5.219 \| -2.621 to 13.06 \| No \| ns \| 0.5487 \| A-J \| \| Column A vs. Column K \| 1.187e-005 \| -7.84 to 7.84 \| No \| ns \| >0.9999 \| A-K \| \| Column A vs. Column L \| 4.868 \| -2.972 to 12.71 \| No \| ns \| 0.6542 \| A-L \| \| Column B vs. Column C \| 0.652 \| -7.188 to 8.492 \| No \| ns \| >0.9999 \| B-C \| \| Column B vs. Column D \| -0.005572 \| -7.846 to 7.835 \| No \| ns \| >0.9999 \| B-D \| \| Column B vs. Column E \| 0.652 \| -7.188 to 8.492 \| No \| ns \| >0.9999 \| B-E \| \| Column B vs. Column F \| -4.378 \| -12.22 to 3.462 \| No \| ns \| 0.7883 \| B-F \| \| Column B vs. Column G \| 0.652 \| -7.188 to 8.492 \| No \| ns \| >0.9999 \| B-G \| \| Column B vs. Column H \| -1.781 \| -9.621 to 6.059 \| No \| ns \| 0.9998 \| B-H \| \| Column B vs. Column I \| 0.652 \| -7.188 to 8.492 \| No \| ns \| >0.9999 \| B-I \| \| Column B vs. Column J \| 5.871 \| -1.969 to 13.71 \| No \| ns \| 0.3595 \| B-J \| \| Column B vs. Column K \| 0.652 \| -7.188 to 8.492 \| No \| ns \| >0.9999 \| B-K \| \| Column B vs. Column L \| 5.52 \| -2.32 to 13.36 \| No \| ns \| 0.4584 \| B-L \| \| Column C vs. Column D \| -0.6576 \| -8.498 to 7.183 \| No \| ns \| >0.9999 \| C-D \| \| Column C vs. Column E \| -4.375e-006 \| -7.84 to 7.84 \| No \| ns \| >0.9999 \| C-E \| \| Column C vs. Column F \| -5.03 \| -12.87 to 2.81 \| No \| ns \| 0.6060 \| C-F \| \| Column C vs. Column G \| -3.75e-006 \| -7.84 to 7.84 \| No \| ns \| >0.9999 \| C-G \| \| Column C vs. Column H \| -2.433 \| -10.27 to 5.407 \| No \| ns \| 0.9969 \| C-H \| \| Column C vs. Column I \| -7.5e-006 \| -7.84 to 7.84 \| No \| ns \| >0.9999 \| C-I \| \| Column C vs. Column J \| 5.219 \| -2.621 to 13.06 \| No \| ns \| 0.5487 \| C-J \| \| Column C vs. Column K \| 0 \| -7.84 to 7.84 \| No \| ns \| >0.9999 \| C-K \| \| Column C vs. Column L \| 4.868 \| -2.972 to 12.71 \| No \| ns \| 0.6543 \| C-L \| \| Column D vs. Column E \| 0.6576 \| -7.183 to 8.498 \| No \| ns \| >0.9999 \| D-E \| \| Column D vs. Column F \| -4.372 \| -12.21 to 3.468 \| No \| ns \| 0.7897 \| D-F \| \| Column D vs. Column G \| 0.6576 \| -7.183 to 8.498 \| No \| ns \| >0.9999 \| D-G \| \| Column D vs. Column H \| -1.775 \| -9.615 to 6.065 \| No \| ns \| 0.9998 \| D-H \| \| Column D vs. Column I \| 0.6576 \| -7.183 to 8.498 \| No \| ns \| >0.9999 \| D-I \| \| Column D vs. Column J \| 5.876 \| -1.964 to 13.72 \| No \| ns \| 0.3580 \| D-J \| \| Column D vs. Column K \| 0.6576 \| -7.183 to 8.498 \| No \| ns \| >0.9999 \| D-K \| \| Column D vs. Column L \| 5.526 \| -2.315 to 13.37 \| No \| ns \| 0.4567 \| D-L \| \| Column E vs. Column F \| -5.03 \| -12.87 to 2.81 \| No \| ns \| 0.6060 \| E-F \| \| Column E vs. Column G \| 6.25e-007 \| -7.84 to 7.84 \| No \| ns \| >0.9999 \| E-G \| \| Column E vs. Column H \| -2.433 \| -10.27 to 5.407 \| No \| ns \| 0.9969 \| E-H \| \| Column E vs. Column I \| -3.125e-006 \| -7.84 to 7.84 \| No \| ns \| >0.9999 \| E-I \| \| Column E vs. Column J \| 5.219 \| -2.621 to 13.06 \| No \| ns \| 0.5487 \| E-J \| \| Column E vs. Column K \| 4.375e-006 \| -7.84 to 7.84 \| No \| ns \| >0.9999 \| E-K \| \| Column E vs. Column L \| 4.868 \| -2.972 to 12.71 \| No \| ns \| 0.6543 \| E-L \| \| Column F vs. Column G \| 5.03 \| -2.81 to 12.87 \| No \| ns \| 0.6060 \| F-G \| \| Column F vs. Column H \| 2.597 \| -5.243 to 10.44 \| No \| ns \| 0.9945 \| F-H \| \| Column F vs. Column I \| 5.03 \| -2.81 to 12.87 \| No \| ns \| 0.6060 \| F-I \| \| Column F vs. Column J \| 10.25 \| 2.408 to 18.09 \| Yes \| ** \| 0.0015 \| F-J \| \| Column F vs. Column K \| 5.03 \| -2.81 to 12.87 \| No \| ns \| 0.6060 \| F-K \| \| Column F vs. Column L \| 9.898 \| 2.058 to 17.74 \| Yes \| ** \| 0.0026 \| F-L \| \| Column G vs. Column H \| -2.433 \| -10.27 to 5.407 \| No \| ns \| 0.9969 \| G-H \| \| Column G vs. Column I \| -3.75e-006 \| -7.84 to 7.84 \| No \| ns \| >0.9999 \| G-I \| \| Column G vs. Column J \| 5.219 \| -2.621 to 13.06 \| No \| ns \| 0.5487 \| G-J \| \| Column G vs. Column K \| 3.75e-006 \| -7.84 to 7.84 \| No \| ns \| >0.9999 \| G-K \| \| Column G vs. Column L \| 4.868 \| -2.972 to 12.71 \| No \| ns \| 0.6543 \| G-L \| \| Column H vs. Column I \| 2.433 \| -5.407 to 10.27 \| No \| ns \| 0.9969 \| H-I \| \| Column H vs. Column J \| 7.652 \| -0.1882 to 15.49 \| No \| ns \| 0.0629 \| H-J \| \| Column H vs. Column K \| 2.433 \| -5.407 to 10.27 \| No \| ns \| 0.9969 \| H-K \| \| Column H vs. Column L \| 7.301 \| -0.5391 to 15.14 \| No \| ns \| 0.0943 \| H-L \| \| Column I vs. Column J \| 5.219 \| -2.621 to 13.06 \| No \| ns \| 0.5487 \| I-J \| \| Column I vs. Column K \| 7.5e-006 \| -7.84 to 7.84 \| No \| ns \| >0.9999 \| I-K \| \| Column I vs. Column L \| 4.868 \| -2.972 to 12.71 \| No \| ns \| 0.6543 \| I-L \| \| Column J vs. Column K \| -5.219 \| -13.06 to 2.621 \| No \| ns \| 0.5487 \| J-K \| \| Column J vs. Column L \| -0.3509 \| -8.191 to 7.489 \| No \| ns \| >0.9999 \| J-L \| \| Column K vs. Column L \| 4.868 \| -2.972 to 12.71 \| No \| ns \| 0.6543 \| K-L \| \|  \|  \|  \|  \|  \|  \|  \| | |
| **Figure 1C MTT** | |
| \| Number of families \| 1 \|  \|  \|  \|  \|  \| \| --- \| --- \| --- \| --- \| --- \| --- \| --- \| \| Number of comparisons per family \| 15 \|  \|  \|  \|  \|  \| \| Alpha \| 0.05 \|  \|  \|  \|  \|  \| \|  \|  \|  \|  \|  \|  \|  \| \| Tukey's multiple comparisons test \| Mean Diff. \| 95.00% CI of diff. \| Significant? \| Summary \| Adjusted P Value \|  \| \|  \|  \|  \|  \|  \|  \|  \| \| Column A vs. Column B \| 0.4724 \| -5.692 to 6.637 \| No \| ns \| >0.9999 \| A-B \| \| Column A vs. Column C \| -1.728 \| -8.711 to 5.256 \| No \| ns \| 0.9804 \| A-C \| \| Column A vs. Column D \| -4.848 \| -11.83 to 2.135 \| No \| ns \| 0.3476 \| A-D \| \| Column A vs. Column E \| -1.728 \| -8.711 to 5.256 \| No \| ns \| 0.9804 \| A-E \| \| Column A vs. Column F \| -8.799 \| -15.78 to -1.815 \| Yes \| ** \| 0.0048 \| A-F \| \| Column B vs. Column C \| -2.2 \| -9.184 to 4.783 \| No \| ns \| 0.9446 \| B-C \| \| Column B vs. Column D \| -5.32 \| -12.3 to 1.663 \| No \| ns \| 0.2462 \| B-D \| \| Column B vs. Column E \| -2.2 \| -9.183 to 4.783 \| No \| ns \| 0.9446 \| B-E \| \| Column B vs. Column F \| -9.271 \| -16.25 to -2.288 \| Yes \| ** \| 0.0024 \| B-F \| \| Column C vs. Column D \| -3.12 \| -10.84 to 4.596 \| No \| ns \| 0.8537 \| C-D \| \| Column C vs. Column E \| 4e-006 \| -7.716 to 7.716 \| No \| ns \| >0.9999 \| C-E \| \| Column C vs. Column F \| -7.071 \| -14.79 to 0.645 \| No \| ns \| 0.0933 \| C-F \| \| Column D vs. Column E \| 3.12 \| -4.596 to 10.84 \| No \| ns \| 0.8537 \| D-E \| \| Column D vs. Column F \| -3.951 \| -11.67 to 3.765 \| No \| ns \| 0.6820 \| D-F \| \| Column E vs. Column F \| -7.071 \| -14.79 to 0.645 \| No \| ns \| 0.0933 \| E-F \| | |
| **Figure 1E p-EGFR-non-parametric test (column statistics in graph prism)** | |
| \| Number of values \| 77 \| 263 \| 354 \| \| --- \| --- \| --- \| --- \| \|  \|  \|  \|  \| \| Minimum \| 11.5 \| 0 \| 0 \| \| 25% Percentile \| 26 \| 39.8 \| 43.83 \| \| Median \| 72.7 \| 197.3 \| 101 \| \| 75% Percentile \| 152.8 \| 298.8 \| 185 \| \| Maximum \| 325.4 \| 625 \| 537 \| \|  \|  \|  \|  \| \| Mean \| 100 \| 195.2 \| 130 \| \| Std. Deviation \| 82.13 \| 155.2 \| 116 \| \| Std. Error of Mean \| 9.359 \| 9.571 \| 6.167 \| \|  \|  \|  \|  \| \| Lower 95% CI of mean \| 81.36 \| 176.4 \| 117.9 \| \| Upper 95% CI of mean \| 118.6 \| 214.1 \| 142.1 \| \|  \|  \|  \|  \| \| Sum \| 7700 \| 51342 \| 46027 \| | |
| **Figure 1E p-EGFR Kruskal-Wallis test** | |
| \| Number of families \| 1 \|  \|  \|  \|  \| \| --- \| --- \| --- \| --- \| --- \| --- \| \| Number of comparisons per family \| 3 \|  \|  \|  \|  \| \| Alpha \| 0.05 \|  \|  \|  \|  \| \|  \|  \|  \|  \|  \|  \| \| Dunn's multiple comparisons test \| Mean rank diff. \| Significant? \| Summary \| Adjusted P Value \|  \| \|  \|  \|  \|  \|  \|  \| \| C vs. L \| -112.9 \| Yes \| **** \| <0.0001 \| A-B \| \| C vs. L+Varl \| -38.28 \| No \| ns \| 0.3868 \| A-C \| \| L vs. L+Varl \| 74.65 \| Yes \| **** \| <0.0001 \| B-C \| | |
| **Figure 1G IL-1β** | |
| \| Number of families \| 1 \|  \|  \|  \|  \|  \| \| --- \| --- \| --- \| --- \| --- \| --- \| --- \| \| Number of comparisons per family \| 3 \|  \|  \|  \|  \|  \| \| Alpha \| 0.05 \|  \|  \|  \|  \|  \| \|  \|  \|  \|  \|  \|  \|  \| \| Tukey's multiple comparisons test \| Mean Diff. \| 95.00% CI of diff. \| Significant? \| Summary \|  \|  \| \|  \|  \|  \|  \|  \|  \|  \| \| C vs. L \| -69.94 \| -100.7 to -39.16 \| Yes \| **** \|  \| A-B \| \| C vs. L+Varl \| -10.31 \| -41.08 to 20.47 \| No \| ns \|  \| A-C \| \| L vs. L+Varl \| 59.63 \| 28.85 to 90.4 \| Yes \| **** \|  \| B-C \| | |
| **Figure 1G iNOS** | |
| \| Number of families \| 1 \|  \|  \|  \|  \|  \| \| --- \| --- \| --- \| --- \| --- \| --- \| --- \| \| Number of comparisons per family \| 3 \|  \|  \|  \|  \|  \| \| Alpha \| 0.05 \|  \|  \|  \|  \|  \| \|  \|  \|  \|  \|  \|  \|  \| \| Tukey's multiple comparisons test \| Mean Diff. \| 95.00% CI of diff. \| Significant? \| Summary \|  \|  \| \|  \|  \|  \|  \|  \|  \|  \| \| C vs. L \| -55.62 \| -85.25 to -25.99 \| Yes \| *** \|  \| A-B \| \| C vs. L+Varl \| -12.83 \| -42.46 to 16.8 \| No \| ns \|  \| A-C \| \| L vs. L+Varl \| 42.79 \| 13.17 to 72.42 \| Yes \| ** \|  \| B-C \| | |
| **Figure 1G COX-2** | |
| \| Number of families \| 1 \|  \|  \|  \|  \|  \| \| --- \| --- \| --- \| --- \| --- \| --- \| --- \| \| Number of comparisons per family \| 3 \|  \|  \|  \|  \|  \| \| Alpha \| 0.05 \|  \|  \|  \|  \|  \| \|  \|  \|  \|  \|  \|  \|  \| \| Tukey's multiple comparisons test \| Mean Diff. \| 95.00% CI of diff. \| Significant? \| Summary \|  \|  \| \|  \|  \|  \|  \|  \|  \|  \| \| C vs. L \| -57.25 \| -89.8 to -24.7 \| Yes \| *** \|  \| A-B \| \| C vs. L+Varl \| -29.58 \| -62.13 to 2.97 \| No \| ns \|  \| A-C \| \| L vs. L+Varl \| 27.67 \| -4.878 to 60.22 \| No \| ns \|  \| B-C \| | |
| **Figure 1G IL-6** | |
| \| Number of families \| 1 \|  \|  \|  \|  \|  \| \| --- \| --- \| --- \| --- \| --- \| --- \| --- \| \| Number of comparisons per family \| 3 \|  \|  \|  \|  \|  \| \| Alpha \| 0.05 \|  \|  \|  \|  \|  \| \|  \|  \|  \|  \|  \|  \|  \| \| Tukey's multiple comparisons test \| Mean Diff. \| 95.00% CI of diff. \| Significant? \| Summary \|  \|  \| \|  \|  \|  \|  \|  \|  \|  \| \| C vs. L \| -155.2 \| -221.2 to -89.23 \| Yes \| **** \|  \| A-B \| \| C vs. L+Varl \| -149.3 \| -215.4 to -83.34 \| Yes \| **** \|  \| A-C \| \| L vs. L+Varl \| 5.891 \| -60.11 to 71.89 \| No \| ns \|  \| B-C \| | |
| **Figure 1I IL-1β** | |
| \| Number of families \| 1 \|  \|  \|  \|  \|  \| \| --- \| --- \| --- \| --- \| --- \| --- \| --- \| \| Number of comparisons per family \| 3 \|  \|  \|  \|  \|  \| \| Alpha \| 0.05 \|  \|  \|  \|  \|  \| \|  \|  \|  \|  \|  \|  \|  \| \| Tukey's multiple comparisons test \| Mean Diff. \| 95.00% CI of diff. \| Significant? \| Summary \|  \|  \| \|  \|  \|  \|  \|  \|  \|  \| \| C vs. L \| -46.39 \| -65.88 to -26.9 \| Yes \| **** \|  \| A-B \| \| C vs. Varl+L \| -10.25 \| -29.75 to 9.242 \| No \| ns \|  \| A-C \| \| L vs. Varl+L \| 36.14 \| 16.64 to 55.63 \| Yes \| *** \|  \| B-C \| | |
| **Figure 1I iNOS** | |
| \| Number of families \| 1 \|  \|  \|  \|  \|  \| \| --- \| --- \| --- \| --- \| --- \| --- \| --- \| \| Number of comparisons per family \| 3 \|  \|  \|  \|  \|  \| \| Alpha \| 0.05 \|  \|  \|  \|  \|  \| \|  \|  \|  \|  \|  \|  \|  \| \| Tukey's multiple comparisons test \| Mean Diff. \| 95.00% CI of diff. \| Significant? \| Summary \|  \|  \| \|  \|  \|  \|  \|  \|  \|  \| \| C vs. L \| -38.54 \| -66.43 to -10.66 \| Yes \| ** \|  \| A-B \| \| C vs. Varl+L \| -21.2 \| -49.08 to 6.686 \| No \| ns \|  \| A-C \| \| L vs. Varl+L \| 17.34 \| -10.54 to 45.23 \| No \| ns \|  \| B-C \| | |
| **Figure 1I COX-2** | |
| \| Number of families \| 1 \|  \|  \|  \|  \|  \| \| --- \| --- \| --- \| --- \| --- \| --- \| --- \| \| Number of comparisons per family \| 3 \|  \|  \|  \|  \|  \| \| Alpha \| 0.05 \|  \|  \|  \|  \|  \| \|  \|  \|  \|  \|  \|  \|  \| \| Tukey's multiple comparisons test \| Mean Diff. \| 95.00% CI of diff. \| Significant? \| Summary \|  \|  \| \|  \|  \|  \|  \|  \|  \|  \| \| C vs. L \| -34.25 \| -60.9 to -7.592 \| Yes \| ** \|  \| A-B \| \| C vs. Varl+L \| -23.48 \| -50.14 to 3.174 \| No \| ns \|  \| A-C \| \| L vs. Varl+L \| 10.77 \| -15.89 to 37.42 \| No \| ns \|  \| B-C \| | |
| **Figure 1I IL-6** | |
| \| Number of families \| 1 \|  \|  \|  \|  \|  \| \| --- \| --- \| --- \| --- \| --- \| --- \| --- \| \| Number of comparisons per family \| 3 \|  \|  \|  \|  \|  \| \| Alpha \| 0.05 \|  \|  \|  \|  \|  \| \|  \|  \|  \|  \|  \|  \|  \| \| Tukey's multiple comparisons test \| Mean Diff. \| 95.00% CI of diff. \| Significant? \| Summary \|  \|  \| \|  \|  \|  \|  \|  \|  \|  \| \| C vs. L \| -123.5 \| -176.5 to -70.54 \| Yes \| **** \|  \| A-B \| \| C vs. L+Varl \| -112.7 \| -165.7 to -59.74 \| Yes \| **** \|  \| A-C \| \| L vs. L+Varl \| 10.81 \| -42.15 to 63.77 \| No \| ns \|  \| B-C \| | |
| **Figure 2B IL-1β** | |
| \| Number of families \| 1 \|  \|  \|  \|  \|  \| \| --- \| --- \| --- \| --- \| --- \| --- \| --- \| \| Number of comparisons per family \| 10 \|  \|  \|  \|  \|  \| \| Alpha \| 0.05 \|  \|  \|  \|  \|  \| \|  \|  \|  \|  \|  \|  \|  \| \| Tukey's multiple comparisons test \| Mean Diff. \| 95.00% CI of diff. \| Significant? \| Summary \| Adjusted P Value \|  \| \|  \|  \|  \|  \|  \|  \|  \| \| C vs. L \| -44.97 \| -65.87 to -24.07 \| Yes \| **** \| <0.0001 \| A-B \| \| C vs. L+Varl \| -16.57 \| -37.47 to 4.331 \| No \| ns \| 0.1852 \| A-C \| \| C vs. L+TAK242 \| 10.19 \| -10.71 to 31.09 \| No \| ns \| 0.6530 \| A-D \| \| C vs. L+Varl+ TAK242 \| 23.39 \| 2.489 to 44.29 \| Yes \| * \| 0.0205 \| A-E \| \| L vs. L+Varl \| 28.4 \| 7.504 to 49.3 \| Yes \| ** \| 0.0026 \| B-C \| \| L vs. L+TAK242 \| 55.16 \| 34.26 to 76.06 \| Yes \| **** \| <0.0001 \| B-D \| \| L vs. L+Varl+ TAK242 \| 68.36 \| 47.46 to 89.26 \| Yes \| **** \| <0.0001 \| B-E \| \| L+Varl vs. L+TAK242 \| 26.76 \| 5.859 to 47.66 \| Yes \| ** \| 0.0054 \| C-D \| \| L+Varl vs. L+Varl+ TAK242 \| 39.96 \| 19.06 to 60.86 \| Yes \| **** \| <0.0001 \| C-E \| \| L+TAK242 vs. L+Varl+ TAK242 \| 13.2 \| -7.701 to 34.1 \| No \| ns \| 0.4014 \| D-E \| | |
| **Figure 2C iNOS** | |
| \| Number of families \| 1 \|  \|  \|  \|  \|  \| \| --- \| --- \| --- \| --- \| --- \| --- \| --- \| \| Number of comparisons per family \| 10 \|  \|  \|  \|  \|  \| \| Alpha \| 0.05 \|  \|  \|  \|  \|  \| \|  \|  \|  \|  \|  \|  \|  \| \| Tukey's multiple comparisons test \| Mean Diff. \| 95.00% CI of diff. \| Significant? \| Summary \| Adjusted P Value \|  \| \|  \|  \|  \|  \|  \|  \|  \| \| C vs. L \| -96.2 \| -125 to -67.36 \| Yes \| **** \| <0.0001 \| A-B \| \| C vs. L+Varl \| -54.52 \| -83.36 to -25.68 \| Yes \| **** \| <0.0001 \| A-C \| \| C vs. L+TAK242 \| 15.2 \| -13.64 to 44.04 \| No \| ns \| 0.5829 \| A-D \| \| C vs. L+Varl+ TAK242 \| 21.8 \| -7.044 to 50.64 \| No \| ns \| 0.2256 \| A-E \| \| L vs. L+Varl \| 41.68 \| 12.84 to 70.52 \| Yes \| ** \| 0.0012 \| B-C \| \| L vs. L+TAK242 \| 111.4 \| 82.56 to 140.2 \| Yes \| **** \| <0.0001 \| B-D \| \| L vs. L+Varl+ TAK242 \| 118 \| 89.16 to 146.8 \| Yes \| **** \| <0.0001 \| B-E \| \| L+Varl vs. L+TAK242 \| 69.72 \| 40.88 to 98.56 \| Yes \| **** \| <0.0001 \| C-D \| \| L+Varl vs. L+Varl+ TAK242 \| 76.32 \| 47.48 to 105.2 \| Yes \| **** \| <0.0001 \| C-E \| \| L+TAK242 vs. L+Varl+ TAK242 \| 6.596 \| -22.24 to 35.44 \| No \| ns \| 0.9681 \| D-E \| | |
| **Figure 2F p-AKT non-parametric test (column statistics in graph prism)** | |
| \| Number of values \| 394 \| 460 \| 326 \| \| --- \| --- \| --- \| --- \| \|  \|  \|  \|  \| \| Minimum \| 28.4 \| 46.1 \| 38.9 \| \| 25% Percentile \| 74.6 \| 99.6 \| 69.38 \| \| Median \| 95.6 \| 129 \| 84.15 \| \| 75% Percentile \| 118.8 \| 162.2 \| 95.43 \| \| Maximum \| 225.1 \| 306.3 \| 152.3 \| \|  \|  \|  \|  \| \| Mean \| 98.18 \| 135.8 \| 83.62 \| \| Std. Deviation \| 33.24 \| 47.84 \| 20.18 \| \| Std. Error of Mean \| 1.675 \| 2.23 \| 1.118 \| \|  \|  \|  \|  \| \| Lower 95% CI of mean \| 94.89 \| 131.4 \| 81.42 \| \| Upper 95% CI of mean \| 101.5 \| 140.2 \| 85.82 \| \|  \|  \|  \|  \| \| Sum \| 38682 \| 62459 \| 27261 \| | |
| **Figure 2F p-AKT Kruskal-Wallis test** | |
| \| Number of families \| 1 \|  \|  \|  \|  \| \| --- \| --- \| --- \| --- \| --- \| --- \| \| Number of comparisons per family \| 3 \|  \|  \|  \|  \| \| Alpha \| 0.05 \|  \|  \|  \|  \| \|  \|  \|  \|  \|  \|  \| \| Dunn's multiple comparisons test \| Mean rank diff. \| Significant? \| Summary \| Adjusted P Value \|  \| \|  \|  \|  \|  \|  \|  \| \| C vs. L \| -273.3 \| Yes \| **** \| <0.0001 \| A-B \| \| C vs. L+Varl \| 143.6 \| Yes \| **** \| <0.0001 \| A-C \| \| L vs. L+Varl \| 416.9 \| Yes \| **** \| <0.0001 \| B-C \| | |
| **Figure 2G p-FAK non-parametric test (column statistics in graph prism)** | |
| \| Number of values \| 331 \| 332 \| 358 \| \| --- \| --- \| --- \| --- \| \|  \|  \|  \|  \| \| Minimum \| 14.7 \| 57.05 \| 44.6 \| \| 25% Percentile \| 60.36 \| 126.6 \| 102.6 \| \| Median \| 103.3 \| 148.7 \| 118.6 \| \| 75% Percentile \| 135.5 \| 179.7 \| 137.6 \| \| Maximum \| 261.9 \| 337.2 \| 277.2 \| \|  \|  \|  \|  \| \| Mean \| 100 \| 157 \| 122.3 \| \| Std. Deviation \| 43.44 \| 42.28 \| 29.51 \| \| Std. Error of Mean \| 2.388 \| 2.321 \| 1.559 \| \|  \|  \|  \|  \| \| Lower 95% CI of mean \| 95.3 \| 152.4 \| 119.2 \| \| Upper 95% CI of mean \| 104.7 \| 161.5 \| 125.3 \| \|  \|  \|  \|  \| \| Sum \| 33100 \| 52113 \| 43775 \| | |
| **Figure 2G p-FAK Kruskal-Wallis test** | |
| \| Number of families \| 1 \|  \|  \|  \|  \| \| --- \| --- \| --- \| --- \| --- \| --- \| \| Number of comparisons per family \| 3 \|  \|  \|  \|  \| \| Alpha \| 0.05 \|  \|  \|  \|  \| \|  \|  \|  \|  \|  \|  \| \| Dunn's multiple comparisons test \| Mean rank diff. \| Significant? \| Summary \| Adjusted P Value \|  \| \|  \|  \|  \|  \|  \|  \| \| C vs. L \| -359.6 \| Yes \| **** \| <0.0001 \| A-B \| \| C vs. L+Varl \| -119.2 \| Yes \| **** \| <0.0001 \| A-C \| \| L vs. L+Varl \| 240.4 \| Yes \| **** \| <0.0001 \| B-C \| | |
| **Figure 2H p-AKT** | |
| \| Number of families \| 1 \|  \|  \|  \|  \|  \| \| --- \| --- \| --- \| --- \| --- \| --- \| --- \| \| Number of comparisons per family \| 3 \|  \|  \|  \|  \|  \| \| Alpha \| 0.05 \|  \|  \|  \|  \|  \| \|  \|  \|  \|  \|  \|  \|  \| \| Tukey's multiple comparisons test \| Mean Diff. \| 95.00% CI of diff. \| Significant? \| Summary \|  \|  \| \|  \|  \|  \|  \|  \|  \|  \| \| C vs. L \| -76.36 \| -130.3 to -22.44 \| Yes \| ** \|  \| A-B \| \| C vs. L+Varl \| -3.727 \| -57.64 to 50.19 \| No \| ns \|  \| A-C \| \| L vs. L+Varl \| 72.63 \| 18.71 to 126.5 \| Yes \| ** \|  \| B-C \| | |
| **Figure 2I p-FAK** | |
| \| Number of families \| 1 \|  \|  \|  \|  \|  \| \| --- \| --- \| --- \| --- \| --- \| --- \| --- \| \| Number of comparisons per family \| 3 \|  \|  \|  \|  \|  \| \| Alpha \| 0.05 \|  \|  \|  \|  \|  \| \|  \|  \|  \|  \|  \|  \|  \| \| Tukey's multiple comparisons test \| Mean Diff. \| 95.00% CI of diff. \| Significant? \| Summary \|  \|  \| \|  \|  \|  \|  \|  \|  \|  \| \| C vs. L \| -28.47 \| -55.15 to -1.798 \| Yes \| * \|  \| A-B \| \| C vs. L+Varl \| 0.13 \| -26.54 to 26.8 \| No \| ns \|  \| A-C \| \| L vs. L+Varl \| 28.6 \| 1.928 to 55.28 \| Yes \| * \|  \| B-C \| | |
| **Figure 3B p-NF-kB non-parametric test (column statistics in graph prism)** | |
| \| Number of values \| 274 \| 434 \| 481 \| \| --- \| --- \| --- \| --- \| \|  \|  \|  \|  \| \| Minimum \| 36.72 \| 50.65 \| 25.31 \| \| 25% Percentile \| 72.7 \| 119.5 \| 66.23 \| \| Median \| 94.5 \| 161.9 \| 89.72 \| \| 75% Percentile \| 119 \| 213.4 \| 122.6 \| \| Maximum \| 253 \| 318.9 \| 303.7 \| \|  \|  \|  \|  \| \| Mean \| 100 \| 169.5 \| 99.07 \| \| Std. Deviation \| 37.38 \| 61.73 \| 46.78 \| \| Std. Error of Mean \| 2.258 \| 2.963 \| 2.133 \| \|  \|  \|  \|  \| \| Lower 95% CI of mean \| 95.55 \| 163.7 \| 94.88 \| \| Upper 95% CI of mean \| 104.4 \| 175.4 \| 103.3 \| \|  \|  \|  \|  \| \| Sum \| 27400 \| 73583 \| 47652 \| | |
| **Figure 3B p-NF-kB Kruskal-Wallis test** | |
| \| Number of families \| 1 \|  \|  \|  \|  \| \| --- \| --- \| --- \| --- \| --- \| --- \| \| Number of comparisons per family \| 3 \|  \|  \|  \|  \| \| Alpha \| 0.05 \|  \|  \|  \|  \| \|  \|  \|  \|  \|  \|  \| \| Dunn's multiple comparisons test \| Mean rank diff. \| Significant? \| Summary \| Adjusted P Value \|  \| \|  \|  \|  \|  \|  \|  \| \| C vs. L \| -376.6 \| Yes \| **** \| <0.0001 \| A-B \| \| C vs. L+Varl \| 23.92 \| No \| ns \| >0.9999 \| A-C \| \| L vs. L+Varl \| 400.6 \| Yes \| **** \| <0.0001 \| B-C \| | |
| **Figure 3D NF-kB** | |
| \| Table Analyzed \| NF-kB \| \| --- \| --- \| \|  \|  \| \| Column B \| L \| \| vs. \| vs. \| \| Column A \| C \| \|  \|  \| \| Unpaired t test \|  \| \| P value \| 0.0360 \| \| P value summary \| * \| \| Significantly different (P < 0.05)? \| Yes \| \| One- or two-tailed P value? \| Two-tailed \| \| t, df \| t=2.516 df=8 \| | \| Table Analyzed \| NF-kB \| \| --- \| --- \| \|  \|  \| \| Column C \| L+Varl \| \| vs. \| vs. \| \| Column B \| L \| \|  \|  \| \| Paired t test \|  \| \| P value \| 0.0459 \| \| P value summary \| * \| \| Significantly different (P < 0.05)? \| Yes \| \| One- or two-tailed P value? \| One-tailed \| \| t, df \| t=2.207 df=4 \| \| Number of pairs \| 5 \| |
| **Figure 3E PCNA** | |
| \| Number of families \| 1 \|  \|  \|  \|  \|  \| \| --- \| --- \| --- \| --- \| --- \| --- \| --- \| \| Number of comparisons per family \| 3 \|  \|  \|  \|  \|  \| \| Alpha \| 0.05 \|  \|  \|  \|  \|  \| \|  \|  \|  \|  \|  \|  \|  \| \| Tukey's multiple comparisons test \| Mean Diff. \| 95.00% CI of diff. \| Significant? \| Summary \|  \|  \| \|  \|  \|  \|  \|  \|  \|  \| \| C vs. L \| 17.75 \| -4.485 to 39.99 \| No \| ns \|  \| A-B \| \| C vs. L+Varl \| 19.5 \| -2.735 to 41.74 \| No \| ns \|  \| A-C \| \| L vs. L+Varl \| 1.75 \| -20.49 to 23.99 \| No \| ns \|  \| B-C \| | |
| **Figure 3F NLRP3** | |
| \| Number of families \| 1 \|  \|  \|  \|  \|  \| \| --- \| --- \| --- \| --- \| --- \| --- \| --- \| \| Number of comparisons per family \| 3 \|  \|  \|  \|  \|  \| \| Alpha \| 0.05 \|  \|  \|  \|  \|  \| \|  \|  \|  \|  \|  \|  \|  \| \| Tukey's multiple comparisons test \| Mean Diff. \| 95.00% CI of diff. \| Significant? \| Summary \|  \|  \| \|  \|  \|  \|  \|  \|  \|  \| \| C vs. L \| -1.752 \| -2.124 to -1.38 \| Yes \| **** \|  \| A-B \| \| C vs. L+Varl \| -1.31 \| -1.682 to -0.9383 \| Yes \| **** \|  \| A-C \| \| L vs. L+Varl \| 0.442 \| 0.07022 to 0.8139 \| Yes \| * \|  \| B-C \| | |
| **Figure 3G pro-IL-1β** | |
| \| Number of families \| 1 \|  \|  \|  \|  \|  \| \| --- \| --- \| --- \| --- \| --- \| --- \| --- \| \| Number of comparisons per family \| 3 \|  \|  \|  \|  \|  \| \| Alpha \| 0.05 \|  \|  \|  \|  \|  \| \|  \|  \|  \|  \|  \|  \|  \| \| Tukey's multiple comparisons test \| Mean Diff. \| 95.00% CI of diff. \| Significant? \| Summary \|  \|  \| \|  \|  \|  \|  \|  \|  \|  \| \| C vs. L \| -32.37 \| -38.77 to -25.97 \| Yes \| **** \|  \| A-B \| \| C vs. L+Varl \| -11.56 \| -17.96 to -5.16 \| Yes \| *** \|  \| A-C \| \| L vs. L+Varl \| 20.81 \| 14.41 to 27.21 \| Yes \| **** \|  \| B-C \| | |
| **Figure 3H IL-1β** | |
| \| Number of families \| 1 \|  \|  \|  \|  \|  \| \| --- \| --- \| --- \| --- \| --- \| --- \| --- \| \| Number of comparisons per family \| 3 \|  \|  \|  \|  \|  \| \| Alpha \| 0.05 \|  \|  \|  \|  \|  \| \|  \|  \|  \|  \|  \|  \|  \| \| Tukey's multiple comparisons test \| Mean Diff. \| 95.00% CI of diff. \| Significant? \| Summary \|  \|  \| \|  \|  \|  \|  \|  \|  \|  \| \| C vs. L \| -20.83 \| -23.58 to -18.09 \| Yes \| **** \|  \| A-B \| \| C vs. L+Varl \| -7.453 \| -10.2 to -4.705 \| Yes \| **** \|  \| A-C \| \| L vs. L+Varl \| 13.38 \| 10.63 to 16.13 \| Yes \| **** \|  \| B-C \| | |
| **Figure 3I NLRP3 mRNA-scrambled siRNA** | |
| \| Number of families \| 1 \|  \|  \|  \|  \|  \| \| --- \| --- \| --- \| --- \| --- \| --- \| --- \| \| Number of comparisons per family \| 3 \|  \|  \|  \|  \|  \| \| Alpha \| 0.05 \|  \|  \|  \|  \|  \| \|  \|  \|  \|  \|  \|  \|  \| \| Tukey's multiple comparisons test \| Mean Diff. \| 95.00% CI of diff. \| Significant? \| Summary \| Adjusted P Value \|  \| \|  \|  \|  \|  \|  \|  \|  \| \| - vs. + \| -3.616 \| -5.881 to -1.352 \| Yes \| ** \| 0.0017 \| A-B \| \| - vs. + \| -0.3163 \| -2.581 to 1.948 \| No \| ns \| 0.9342 \| A-C \| \| + vs. + \| 3.3 \| 1.035 to 5.564 \| Yes \| ** \| 0.0039 \| B-C \| | |
| **Figure 3I NLRP3 mRNA-NLRP3 siRNA** | |
| \| Number of families \| 1 \|  \|  \|  \|  \|  \| \| --- \| --- \| --- \| --- \| --- \| --- \| --- \| \| Number of comparisons per family \| 3 \|  \|  \|  \|  \|  \| \| Alpha \| 0.05 \|  \|  \|  \|  \|  \| \|  \|  \|  \|  \|  \|  \|  \| \| Tukey's multiple comparisons test \| Mean Diff. \| 95.00% CI of diff. \| Significant? \| Summary \| Adjusted P Value \|  \| \|  \|  \|  \|  \|  \|  \|  \| \| - vs. + \| -0.3113 \| -0.6635 to 0.04092 \| No \| ns \| 0.0896 \| E-F \| \| - vs. + \| -0.1242 \| -0.4764 to 0.228 \| No \| ns \| 0.6532 \| E-G \| \| + vs. + \| 0.1871 \| -0.1651 to 0.5393 \| No \| ns \| 0.3901 \| F-G \| | |
| **Figure 3J IL-1β mRNA-scrambled siRNA** | |
| \| Number of families \| 1 \|  \|  \|  \|  \|  \| \| --- \| --- \| --- \| --- \| --- \| --- \| --- \| \| Number of comparisons per family \| 3 \|  \|  \|  \|  \|  \| \| Alpha \| 0.05 \|  \|  \|  \|  \|  \| \|  \|  \|  \|  \|  \|  \|  \| \| Tukey's multiple comparisons test \| Mean Diff. \| 95.00% CI of diff. \| Significant? \| Summary \| Adjusted P Value \|  \| \|  \|  \|  \|  \|  \|  \|  \| \| - vs. + \| -21.54 \| -30.27 to -12.81 \| Yes \| **** \| <0.0001 \| A-B \| \| - vs. + \| -12.28 \| -21.02 to -3.55 \| Yes \| ** \| 0.0052 \| A-C \| \| + vs. + \| 9.258 \| 0.5248 to 17.99 \| Yes \| * \| 0.0365 \| B-C \| | |
| **Figure 3J IL-1β mRNA-NLRP3 siRNA** | |
| \| Number of families \| 1 \|  \|  \|  \|  \|  \| \| --- \| --- \| --- \| --- \| --- \| --- \| --- \| \| Number of comparisons per family \| 3 \|  \|  \|  \|  \|  \| \| Alpha \| 0.05 \|  \|  \|  \|  \|  \| \|  \|  \|  \|  \|  \|  \|  \| \| Tukey's multiple comparisons test \| Mean Diff. \| 95.00% CI of diff. \| Significant? \| Summary \| Adjusted P Value \|  \| \|  \|  \|  \|  \|  \|  \|  \| \| - vs. + \| -19.14 \| -28.36 to -9.928 \| Yes \| *** \| 0.0001 \| E-F \| \| - vs. + \| -15.74 \| -24.64 to -6.837 \| Yes \| *** \| 0.0007 \| E-G \| \| + vs. + \| 3.404 \| -5.811 to 12.62 \| No \| ns \| 0.6254 \| F-G \| | |
| **Figure 4A IL-1β** | |
| \| Number of families \| 1 \|  \|  \|  \|  \|  \| \| --- \| --- \| --- \| --- \| --- \| --- \| --- \| \| Number of comparisons per family \| 3 \|  \|  \|  \|  \|  \| \| Alpha \| 0.05 \|  \|  \|  \|  \|  \| \|  \|  \|  \|  \|  \|  \|  \| \| Tukey's multiple comparisons test \| Mean Diff. \| 95.00% CI of diff. \| Significant? \| Summary \|  \|  \| \|  \|  \|  \|  \|  \|  \|  \| \| C vs. L \| -399.3 \| -494.9 to -303.7 \| Yes \| **** \|  \| A-B \| \| C vs. L+Varl \| -293.5 \| -389.1 to -197.9 \| Yes \| **** \|  \| A-C \| \| L vs. L+Varl \| 105.8 \| 10.23 to 201.4 \| Yes \| * \|  \| B-C \| | |
| **Figure 4A COX-2** | |
| \| Number of families \| 1 \|  \|  \|  \|  \|  \| \| --- \| --- \| --- \| --- \| --- \| --- \| --- \| \| Number of comparisons per family \| 3 \|  \|  \|  \|  \|  \| \| Alpha \| 0.05 \|  \|  \|  \|  \|  \| \|  \|  \|  \|  \|  \|  \|  \| \| Tukey's multiple comparisons test \| Mean Diff. \| 95.00% CI of diff. \| Significant? \| Summary \|  \|  \| \|  \|  \|  \|  \|  \|  \|  \| \| C vs. L \| -135.8 \| -172.2 to -99.43 \| Yes \| **** \|  \| A-B \| \| C vs. L+Varl \| -132.4 \| -168.8 to -95.99 \| Yes \| **** \|  \| A-C \| \| L vs. L+Varl \| 3.433 \| -32.95 to 39.81 \| No \| ns \|  \| B-C \| | |
| **Figure 4A IL-6** | |
| \| Number of families \| 1 \|  \|  \|  \|  \|  \| \| --- \| --- \| --- \| --- \| --- \| --- \| --- \| \| Number of comparisons per family \| 3 \|  \|  \|  \|  \|  \| \| Alpha \| 0.05 \|  \|  \|  \|  \|  \| \|  \|  \|  \|  \|  \|  \|  \| \| Tukey's multiple comparisons test \| Mean Diff. \| 95.00% CI of diff. \| Significant? \| Summary \|  \|  \| \|  \|  \|  \|  \|  \|  \|  \| \| C vs. L \| -691.8 \| -837.2 to -546.4 \| Yes \| **** \|  \| A-B \| \| C vs. L+Varl \| -627.4 \| -772.8 to -482 \| Yes \| **** \|  \| A-C \| \| L vs. L+Varl \| 64.39 \| -81.02 to 209.8 \| No \| ns \|  \| B-C \| | |
| **Figure 4A iNOS** | |
| \| Number of families \| 1 \|  \|  \|  \|  \|  \| \| --- \| --- \| --- \| --- \| --- \| --- \| --- \| \| Number of comparisons per family \| 3 \|  \|  \|  \|  \|  \| \| Alpha \| 0.05 \|  \|  \|  \|  \|  \| \|  \|  \|  \|  \|  \|  \|  \| \| Tukey's multiple comparisons test \| Mean Diff. \| 95.00% CI of diff. \| Significant? \| Summary \|  \|  \| \|  \|  \|  \|  \|  \|  \|  \| \| C vs. L \| -323.7 \| -413.5 to -233.9 \| Yes \| **** \|  \| A-B \| \| C vs. L+Varl \| -305 \| -394.8 to -215.2 \| Yes \| **** \|  \| A-C \| \| L vs. L+Varl \| 18.62 \| -71.17 to 108.4 \| No \| ns \|  \| B-C \| | |
| **Figure 4B p-AKT** | |
| \| Number of families \| 1 \|  \|  \|  \|  \|  \| \| --- \| --- \| --- \| --- \| --- \| --- \| --- \| \| Number of comparisons per family \| 3 \|  \|  \|  \|  \|  \| \| Alpha \| 0.05 \|  \|  \|  \|  \|  \| \|  \|  \|  \|  \|  \|  \|  \| \| Tukey's multiple comparisons test \| Mean Diff. \| 95.00% CI of diff. \| Significant? \| Summary \|  \|  \| \|  \|  \|  \|  \|  \|  \|  \| \| C vs. L \| -155.1 \| -221.2 to -89.07 \| Yes \| *** \|  \| A-B \| \| C vs. L+Varl \| 72.69 \| 6.619 to 138.8 \| Yes \| * \|  \| A-C \| \| L vs. L+Varl \| 227.8 \| 161.8 to 293.9 \| Yes \| **** \|  \| B-C \| | |
| **Figure 4B β-actin** | |
| \| Number of families \| 1 \|  \|  \|  \|  \|  \| \| --- \| --- \| --- \| --- \| --- \| --- \| --- \| \| Number of comparisons per family \| 3 \|  \|  \|  \|  \|  \| \| Alpha \| 0.05 \|  \|  \|  \|  \|  \| \|  \|  \|  \|  \|  \|  \|  \| \| Tukey's multiple comparisons test \| Mean Diff. \| 95.00% CI of diff. \| Significant? \| Summary \| Adjusted P Value \|  \| \|  \|  \|  \|  \|  \|  \|  \| \| Veh vs. LPS \| -14.25 \| -81.11 to 52.61 \| No \| ns \| 0.8390 \| A-B \| \| Veh vs. LPS+Varl \| 27.22 \| -39.63 to 94.08 \| No \| ns \| 0.5400 \| A-C \| \| LPS vs. LPS+Varl \| 41.48 \| -25.38 to 108.3 \| No \| ns \| 0.2616 \| B-C \| | |
| **Figure 4C p-AKT non-parametric test (column statistics in graph prism)** | |
| \| Number of values \| 50 \| 47 \| 27 \| \| --- \| --- \| --- \| --- \| \|  \|  \|  \|  \| \| Minimum \| 0.65 \| 53.5 \| 22.9 \| \| 25% Percentile \| 43.34 \| 132.7 \| 46.4 \| \| Median \| 93.01 \| 213.7 \| 58.6 \| \| 75% Percentile \| 126.7 \| 434.8 \| 80 \| \| Maximum \| 325.2 \| 882.4 \| 181.5 \| \|  \|  \|  \|  \| \| Mean \| 100 \| 290.1 \| 68.35 \| \| Std. Deviation \| 70.72 \| 200.9 \| 32.77 \| \| Std. Error of Mean \| 10 \| 29.3 \| 6.307 \| \|  \|  \|  \|  \| \| Lower 95% CI of mean \| 79.9 \| 231.1 \| 55.39 \| \| Upper 95% CI of mean \| 120.1 \| 349.1 \| 81.32 \| \|  \|  \|  \|  \| \| Sum \| 5000 \| 13634 \| 1846 \| | |
| **Figure 4C p-AKT Kruskal-Wallis test** | |
| \| Number of families \| 1 \|  \|  \|  \|  \| \| --- \| --- \| --- \| --- \| --- \| --- \| \| Number of comparisons per family \| 3 \|  \|  \|  \|  \| \| Alpha \| 0.05 \|  \|  \|  \|  \| \|  \|  \|  \|  \|  \|  \| \| Dunn's multiple comparisons test \| Mean rank diff. \| Significant? \| Summary \| Adjusted P Value \|  \| \|  \|  \|  \|  \|  \|  \| \| C vs. L \| -376.6 \| Yes \| **** \| <0.0001 \| A-B \| \| C vs. L+Varl \| 23.92 \| No \| ns \| >0.9999 \| A-C \| \| L vs. L+Varl \| 400.6 \| Yes \| **** \| <0.0001 \| B-C \| | |
| **Figure 4D NLRP3** | |
| \| Number of families \| 1 \|  \|  \|  \|  \|  \| \| --- \| --- \| --- \| --- \| --- \| --- \| --- \| \| Number of comparisons per family \| 3 \|  \|  \|  \|  \|  \| \| Alpha \| 0.05 \|  \|  \|  \|  \|  \| \|  \|  \|  \|  \|  \|  \|  \| \| Tukey's multiple comparisons test \| Mean Diff. \| 95.00% CI of diff. \| Significant? \| Summary \|  \|  \| \|  \|  \|  \|  \|  \|  \|  \| \| C vs. L \| -36.77 \| -50.16 to -23.39 \| Yes \| **** \|  \| A-B \| \| C vs. L+Varl \| -28.99 \| -42.37 to -15.61 \| Yes \| **** \|  \| A-C \| \| L vs. L+Varl \| 7.788 \| -5.594 to 21.17 \| No \| ns \|  \| B-C \| | |
| **Figure 4D pro-IL-1β** | |
| \| Number of families \| 1 \|  \|  \|  \|  \|  \| \| --- \| --- \| --- \| --- \| --- \| --- \| --- \| \| Number of comparisons per family \| 3 \|  \|  \|  \|  \|  \| \| Alpha \| 0.05 \|  \|  \|  \|  \|  \| \|  \|  \|  \|  \|  \|  \|  \| \| Tukey's multiple comparisons test \| Mean Diff. \| 95.00% CI of diff. \| Significant? \| Summary \|  \|  \| \|  \|  \|  \|  \|  \|  \|  \| \| C vs. L \| -946.9 \| -1216 to -677.9 \| Yes \| **** \|  \| A-B \| \| C vs. L+Varl \| -617.3 \| -886.3 to -348.3 \| Yes \| **** \|  \| A-C \| \| L vs. L+Varl \| 329.6 \| 60.59 to 598.7 \| Yes \| * \|  \| B-C \| | |
| **Figure 4E NLRP3** | |
| \| Number of families \| 1 \|  \|  \|  \|  \|  \| \| --- \| --- \| --- \| --- \| --- \| --- \| --- \| \| Number of comparisons per family \| 3 \|  \|  \|  \|  \|  \| \| Alpha \| 0.05 \|  \|  \|  \|  \|  \| \|  \|  \|  \|  \|  \|  \|  \| \| Tukey's multiple comparisons test \| Mean Diff. \| 95.00% CI of diff. \| Significant? \| Summary \|  \|  \| \|  \|  \|  \|  \|  \|  \|  \| \| C vs. L \| -5.313 \| -6.386 to -4.24 \| Yes \| **** \|  \| A-B \| \| C vs. L+Varl \| -4.036 \| -5.109 to -2.962 \| Yes \| **** \|  \| A-C \| \| L vs. L+Varl \| 1.277 \| 0.2043 to 2.351 \| Yes \| * \|  \| B-C \| | |
| **Figure 4E pro-IL-1β** | |
| \| Number of families \| 1 \|  \|  \|  \|  \|  \| \| --- \| --- \| --- \| --- \| --- \| --- \| --- \| \| Number of comparisons per family \| 3 \|  \|  \|  \|  \|  \| \| Alpha \| 0.05 \|  \|  \|  \|  \|  \| \|  \|  \|  \|  \|  \|  \|  \| \| Tukey's multiple comparisons test \| Mean Diff. \| 95.00% CI of diff. \| Significant? \| Summary \|  \|  \| \|  \|  \|  \|  \|  \|  \|  \| \| C vs. L \| -2001 \| -2395 to -1607 \| Yes \| **** \|  \| A-B \| \| C vs. L+Varl \| -1503 \| -1897 to -1109 \| Yes \| **** \|  \| A-C \| \| L vs. L+Varl \| 497.9 \| 104.2 to 891.6 \| Yes \| * \|  \| B-C \| | |
| **Figure 4E IL-1β** | |
| \| Number of families \| 1 \|  \|  \|  \|  \|  \| \| --- \| --- \| --- \| --- \| --- \| --- \| --- \| \| Number of comparisons per family \| 3 \|  \|  \|  \|  \|  \| \| Alpha \| 0.05 \|  \|  \|  \|  \|  \| \|  \|  \|  \|  \|  \|  \|  \| \| Tukey's multiple comparisons test \| Mean Diff. \| 95.00% CI of diff. \| Significant? \| Summary \|  \|  \| \|  \|  \|  \|  \|  \|  \|  \| \| C vs. L \| -1337 \| -1611 to -1064 \| Yes \| **** \|  \| A-B \| \| C vs. L+Varl \| -1024 \| -1297 to -750.6 \| Yes \| **** \|  \| A-C \| \| L vs. L+Varl \| 313.6 \| 40.36 to 586.8 \| Yes \| * \|  \| B-C \| | |

**Supplementary table 2**. One-way ANOVA (Tukey’s test) or t-test (two-tailed) and significance of the results of the *in vivo* experiments in this study.

| **Figure 5B Iba-1 Fluorescence intensity-Cortex** |
| --- |
| \| Number of families \| 1 \|  \|  \|  \|  \|  \| \| --- \| --- \| --- \| --- \| --- \| --- \| --- \| \| Number of comparisons per family \| 3 \|  \|  \|  \|  \|  \| \| Alpha \| 0.05 \|  \|  \|  \|  \|  \| \|  \|  \|  \|  \|  \|  \|  \| \| Tukey's multiple comparisons test \| Mean Diff. \| 95.00% CI of diff. \| Significant? \| Summary \| Adjusted P Value \|  \| \|  \|  \|  \|  \|  \|  \|  \| \| Veh vs. LPS \| -104.9 \| -152.9 to -56.99 \| Yes \| **** \| <0.0001 \| A-B \| \| Veh vs. LPS  Varlitinib \| -14.78 \| -62.73 to 33.17 \| No \| ns \| 0.7446 \| A-C \| \| LPS vs. LPS  Varlitinib \| 90.15 \| 44.3 to 136 \| Yes \| **** \| <0.0001 \| B-C \| |
| **Figure 5B Iba-1 Fluorescence intensity-CA1** |
| \| Number of families \| 1 \|  \|  \|  \|  \|  \| \| --- \| --- \| --- \| --- \| --- \| --- \| --- \| \| Number of comparisons per family \| 3 \|  \|  \|  \|  \|  \| \| Alpha \| 0.05 \|  \|  \|  \|  \|  \| \|  \|  \|  \|  \|  \|  \|  \| \| Tukey's multiple comparisons test \| Mean Diff. \| 95.00% CI of diff. \| Significant? \| Summary \| Adjusted P Value \|  \| \|  \|  \|  \|  \|  \|  \|  \| \| Veh vs. LPS \| -218.1 \| -317.3 to -118.9 \| Yes \| **** \| <0.0001 \| E-F \| \| Veh vs. LPS  Varlitinib \| -33.07 \| -134.8 to 68.66 \| No \| ns \| 0.7203 \| E-G \| \| LPS vs. LPS  Varlitinib \| 185 \| 88.18 to 281.9 \| Yes \| **** \| <0.0001 \| F-G \| |
| **Figure 5B Iba-1 Fluorescence intensity-DG** |
| \| Number of families \| 1 \|  \|  \|  \|  \|  \| \| --- \| --- \| --- \| --- \| --- \| --- \| --- \| \| Number of comparisons per family \| 3 \|  \|  \|  \|  \|  \| \| Alpha \| 0.05 \|  \|  \|  \|  \|  \| \|  \|  \|  \|  \|  \|  \|  \| \| Tukey's multiple comparisons test \| Mean Diff. \| 95.00% CI of diff. \| Significant? \| Summary \| Adjusted P Value \|  \| \|  \|  \|  \|  \|  \|  \|  \| \| Veh vs. LPS \| -227.3 \| -336.4 to -118.2 \| Yes \| **** \| <0.0001 \| I-J \| \| Veh vs. LPS  Varlitinib \| -80.09 \| -192 to 31.8 \| No \| ns \| 0.2093 \| I-K \| \| LPS vs. LPS  Varlitinib \| 147.2 \| 40.68 to 253.7 \| Yes \| ** \| 0.0039 \| J-K \| |
| **Figure 5B Iba-1^+^ cells-Cortex** |
| \| Number of families \| 1 \|  \|  \|  \|  \|  \| \| --- \| --- \| --- \| --- \| --- \| --- \| --- \| \| Number of comparisons per family \| 3 \|  \|  \|  \|  \|  \| \| Alpha \| 0.05 \|  \|  \|  \|  \|  \| \|  \|  \|  \|  \|  \|  \|  \| \| Tukey's multiple comparisons test \| Mean Diff. \| 95.00% CI of diff. \| Significant? \| Summary \| Adjusted P Value \|  \| \|  \|  \|  \|  \|  \|  \|  \| \| Veh vs. LPS \| -85.05 \| -111.2 to -58.93 \| Yes \| **** \| <0.0001 \| A-B \| \| Veh vs. LPS  Varlitinib \| -40.8 \| -66.76 to -14.84 \| Yes \| *** \| 0.0009 \| A-C \| \| LPS vs. LPS  Varlitinib \| 44.25 \| 19.26 to 69.25 \| Yes \| *** \| 0.0002 \| B-C \| |
| **Figure 5B Iba-1^+^ cells-CA1** |
| \|  \|  \|  \| \| Number of families \| 1 \|  \|  \|  \|  \|  \| \| --- \| --- \| --- \| --- \| --- \| --- \| --- \| \| Number of comparisons per family \| 3 \|  \|  \|  \|  \|  \| \| Alpha \| 0.05 \|  \|  \|  \|  \|  \| \|  \|  \|  \|  \|  \|  \|  \| \| Tukey's multiple comparisons test \| Mean Diff. \| 95.00% CI of diff. \| Significant? \| Summary \| Adjusted P Value \|  \| \|  \|  \|  \|  \|  \|  \|  \| \| Veh vs. LPS \| -149.4 \| -187.8 to -111.1 \| Yes \| **** \| <0.0001 \| E-F \| \| Veh vs. LPS  Varlitinib \| -44.23 \| -83.54 to -4.924 \| Yes \| * \| 0.0234 \| E-G \| \| LPS vs. LPS  Varlitinib \| 105.2 \| 67.79 to 142.6 \| Yes \| **** \| <0.0001 \| F-G \| \|  \|  \|  \| \| --- \| --- \| --- \| --- \| --- \| --- \| --- \| --- \| --- \| --- \| --- \| --- \| --- \| --- \| --- \| --- \| --- \| --- \| --- \| --- \| --- \| --- \| --- \| --- \| --- \| --- \| --- \| --- \| --- \| --- \| --- \| --- \| --- \| --- \| --- \| --- \| --- \| --- \| --- \| --- \| --- \| --- \| --- \| --- \| --- \| --- \| --- \| --- \| --- \| --- \| --- \| --- \| --- \| --- \| --- \| --- \| --- \| --- \| --- \| --- \| --- \| --- \| --- \| --- \| --- \| --- \| --- \| --- \| --- \| --- \| |
| **Figure 5B Iba-1^+^ cells-DG** |
| \| Number of families \| 1 \|  \|  \|  \|  \|  \| \| --- \| --- \| --- \| --- \| --- \| --- \| --- \| \| Number of comparisons per family \| 3 \|  \|  \|  \|  \|  \| \| Alpha \| 0.05 \|  \|  \|  \|  \|  \| \|  \|  \|  \|  \|  \|  \|  \| \| Tukey's multiple comparisons test \| Mean Diff. \| 95.00% CI of diff. \| Significant? \| Summary \| Adjusted P Value \|  \| \|  \|  \|  \|  \|  \|  \|  \| \| Veh vs. LPS \| -176.8 \| -221.3 to -132.3 \| Yes \| **** \| <0.0001 \| I-J \| \| Veh vs. LPS  Varlitinib \| -55.37 \| -101 to -9.727 \| Yes \| * \| 0.0131 \| I-K \| \| LPS vs. LPS  Varlitinib \| 121.4 \| 77.97 to 164.9 \| Yes \| **** \| <0.0001 \| J-K \| |
| **Figure 5B Iba-1^+^ area-Cortex** |
| \| Number of families \| 1 \|  \|  \|  \|  \|  \| \| --- \| --- \| --- \| --- \| --- \| --- \| --- \| \| Number of comparisons per family \| 3 \|  \|  \|  \|  \|  \| \| Alpha \| 0.05 \|  \|  \|  \|  \|  \| \|  \|  \|  \|  \|  \|  \|  \| \| Tukey's multiple comparisons test \| Mean Diff. \| 95.00% CI of diff. \| Significant? \| Summary \| Adjusted P Value \|  \| \|  \|  \|  \|  \|  \|  \|  \| \| Veh vs. LPS \| -165 \| -213.6 to -116.4 \| Yes \| **** \| <0.0001 \| A-B \| \| Veh vs. LPS  Varlitinib \| -66.32 \| -114.9 to -17.75 \| Yes \| ** \| 0.0044 \| A-C \| \| LPS vs. LPS  Varlitinib \| 98.67 \| 52.23 to 145.1 \| Yes \| **** \| <0.0001 \| B-C \| |
| **Figure 5B Iba-1^+^ area-CA1** |
| \| Number of families \| 1 \|  \|  \|  \|  \|  \| \| --- \| --- \| --- \| --- \| --- \| --- \| --- \| \| Number of comparisons per family \| 3 \|  \|  \|  \|  \|  \| \| Alpha \| 0.05 \|  \|  \|  \|  \|  \| \|  \|  \|  \|  \|  \|  \|  \| \| Tukey's multiple comparisons test \| Mean Diff. \| 95.00% CI of diff. \| Significant? \| Summary \| Adjusted P Value \|  \| \|  \|  \|  \|  \|  \|  \|  \| \| Veh vs. LPS \| -442 \| -541.4 to -342.5 \| Yes \| **** \| <0.0001 \| E-F \| \| Veh vs. LPS  Varlitinib \| -100.9 \| -202.9 to 1.087 \| No \| ns \| 0.0532 \| E-G \| \| LPS vs. LPS  Varlitinib \| 341 \| 244 to 438.1 \| Yes \| **** \| <0.0001 \| F-G \| |
| **Figure 5B Iba-1^+^ area-DG** |
| \| Number of families \| 1 \|  \|  \|  \|  \|  \| \| --- \| --- \| --- \| --- \| --- \| --- \| --- \| \| Number of comparisons per family \| 3 \|  \|  \|  \|  \|  \| \| Alpha \| 0.05 \|  \|  \|  \|  \|  \| \|  \|  \|  \|  \|  \|  \|  \| \| Tukey's multiple comparisons test \| Mean Diff. \| 95.00% CI of diff. \| Significant? \| Summary \| Adjusted P Value \|  \| \|  \|  \|  \|  \|  \|  \|  \| \| Veh vs. LPS \| -446.4 \| -537.5 to -355.3 \| Yes \| **** \| <0.0001 \| I-J \| \| Veh vs. LPS  Varlitinib \| -122.1 \| -215.5 to -28.66 \| Yes \| ** \| 0.0068 \| I-K \| \| LPS vs. LPS  Varlitinib \| 324.4 \| 235.4 to 413.3 \| Yes \| **** \| <0.0001 \| J-K \| |
| **Figure 5D GFAP Fluorescence intensity-Cortex** |
| \| Number of families \| 1 \|  \|  \|  \|  \|  \| \| --- \| --- \| --- \| --- \| --- \| --- \| --- \| \| Number of comparisons per family \| 3 \|  \|  \|  \|  \|  \| \| Alpha \| 0.05 \|  \|  \|  \|  \|  \| \|  \|  \|  \|  \|  \|  \|  \| \| Tukey's multiple comparisons test \| Mean Diff. \| 95.00% CI of diff. \| Significant? \| Summary \| Adjusted P Value \|  \| \|  \|  \|  \|  \|  \|  \|  \| \| Veh vs. LPS \| -152 \| -254.3 to -49.76 \| Yes \| ** \| 0.0018 \| A-B \| \| Veh vs. LPS  Varlitinib \| -45.03 \| -152.4 to 62.36 \| No \| ns \| 0.5800 \| A-C \| \| LPS vs. LPS  Varlitinib \| 107 \| 3.844 to 210.1 \| Yes \| * \| 0.0402 \| B-C \| |
| **Figure 5D GFAP Fluorescence intensity-CA1** |
| \| Number of families \| 1 \|  \|  \|  \|  \|  \| \| --- \| --- \| --- \| --- \| --- \| --- \| --- \| \| Number of comparisons per family \| 3 \|  \|  \|  \|  \|  \| \| Alpha \| 0.05 \|  \|  \|  \|  \|  \| \|  \|  \|  \|  \|  \|  \|  \| \| Tukey's multiple comparisons test \| Mean Diff. \| 95.00% CI of diff. \| Significant? \| Summary \| Adjusted P Value \|  \| \|  \|  \|  \|  \|  \|  \|  \| \| Veh vs. LPS \| -39.5 \| -78.72 to -0.2674 \| Yes \| * \| 0.0481 \| E-F \| \| Veh vs. LPS  Varlitinib \| -12.22 \| -52.77 to 28.32 \| No \| ns \| 0.7541 \| E-G \| \| LPS vs. LPS  Varlitinib \| 27.27 \| -11.96 to 66.5 \| No \| ns \| 0.2283 \| F-G \| |
| **Figure 5D GFAP Fluorescence intensity-DG** |
| \| Number of families \| 1 \|  \|  \|  \|  \|  \| \| --- \| --- \| --- \| --- \| --- \| --- \| --- \| \| Number of comparisons per family \| 3 \|  \|  \|  \|  \|  \| \| Alpha \| 0.05 \|  \|  \|  \|  \|  \| \|  \|  \|  \|  \|  \|  \|  \| \| Tukey's multiple comparisons test \| Mean Diff. \| 95.00% CI of diff. \| Significant? \| Summary \| Adjusted P Value \|  \| \|  \|  \|  \|  \|  \|  \|  \| \| Veh vs. LPS \| -69.49 \| -116.5 to -22.5 \| Yes \| ** \| 0.0019 \| I-J \| \| Veh vs. LPS  Varlitinib \| -25.72 \| -74.28 to 22.85 \| No \| ns \| 0.4216 \| I-K \| \| LPS vs. LPS  Varlitinib \| 43.77 \| -3.214 to 90.76 \| No \| ns \| 0.0733 \| J-K \| |
| **Figure 5D GFAP^+^ cells-Cortex** |
| \| Number of families \| 1 \|  \|  \|  \|  \|  \| \| --- \| --- \| --- \| --- \| --- \| --- \| --- \| \| Number of comparisons per family \| 3 \|  \|  \|  \|  \|  \| \| Alpha \| 0.05 \|  \|  \|  \|  \|  \| \|  \|  \|  \|  \|  \|  \|  \| \| Tukey's multiple comparisons test \| Mean Diff. \| 95.00% CI of diff. \| Significant? \| Summary \| Adjusted P Value \|  \| \|  \|  \|  \|  \|  \|  \|  \| \| Veh vs. LPS \| -354.5 \| -542.1 to -167 \| Yes \| **** \| <0.0001 \| A-B \| \| Veh vs. LPS  Varlitinib \| -82.13 \| -277.5 to 113.3 \| No \| ns \| 0.5785 \| A-C \| \| LPS vs. LPS  Varlitinib \| 272.4 \| 84.9 to 459.9 \| Yes \| ** \| 0.0023 \| B-C \| |
| **Figure 5D GFAP^+^ cells-CA1** |
| \| Number of families \| 1 \|  \|  \|  \|  \|  \| \| --- \| --- \| --- \| --- \| --- \| --- \| --- \| \| Number of comparisons per family \| 3 \|  \|  \|  \|  \|  \| \| Alpha \| 0.05 \|  \|  \|  \|  \|  \| \|  \|  \|  \|  \|  \|  \|  \| \| Tukey's multiple comparisons test \| Mean Diff. \| 95.00% CI of diff. \| Significant? \| Summary \| Adjusted P Value \|  \| \|  \|  \|  \|  \|  \|  \|  \| \| Veh vs. LPS \| -69.23 \| -104.1 to -34.32 \| Yes \| **** \| <0.0001 \| E-F \| \| Veh vs. LPS  Varlitinib \| -0.7307 \| -36.82 to 35.36 \| No \| ns \| 0.9987 \| E-G \| \| LPS vs. LPS  Varlitinib \| 68.5 \| 33.59 to 103.4 \| Yes \| **** \| <0.0001 \| F-G \| |
| **Figure 5D GFAP^+^ cells-DG** |
| \| Number of families \| 1 \|  \|  \|  \|  \|  \| \| --- \| --- \| --- \| --- \| --- \| --- \| --- \| \| Number of comparisons per family \| 3 \|  \|  \|  \|  \|  \| \| Alpha \| 0.05 \|  \|  \|  \|  \|  \| \|  \|  \|  \|  \|  \|  \|  \| \| Tukey's multiple comparisons test \| Mean Diff. \| 95.00% CI of diff. \| Significant? \| Summary \| Adjusted P Value \|  \| \|  \|  \|  \|  \|  \|  \|  \| \| Veh vs. LPS \| -96.72 \| -139.3 to -54.11 \| Yes \| **** \| <0.0001 \| I-J \| \| Veh vs. LPS  Varlitinib \| -17.48 \| -61.52 to 26.56 \| No \| ns \| 0.6139 \| I-K \| \| LPS vs. LPS  Varlitinib \| 79.24 \| 36.63 to 121.8 \| Yes \| **** \| <0.0001 \| J-K \| |
| **Figure 5D GFAP^+^ area-Cortex** |
| \| Number of families \| 1 \|  \|  \|  \|  \|  \| \| --- \| --- \| --- \| --- \| --- \| --- \| --- \| \| Number of comparisons per family \| 3 \|  \|  \|  \|  \|  \| \| Alpha \| 0.05 \|  \|  \|  \|  \|  \| \|  \|  \|  \|  \|  \|  \|  \| \| Tukey's multiple comparisons test \| Mean Diff. \| 95.00% CI of diff. \| Significant? \| Summary \| Adjusted P Value \|  \| \|  \|  \|  \|  \|  \|  \|  \| \| Veh vs. LPS \| -187.6 \| -277.1 to -98.15 \| Yes \| **** \| <0.0001 \| A-B \| \| Veh vs. LPS  Varlitinib \| -38.78 \| -132 to 54.48 \| No \| ns \| 0.5853 \| A-C \| \| LPS vs. LPS  Varlitinib \| 148.9 \| 59.37 to 238.4 \| Yes \| *** \| 0.0004 \| B-C \| |
| **Figure 5D GFAP^+^ area-CA1** |
| \| Number of families \| 1 \|  \|  \|  \|  \|  \| \| --- \| --- \| --- \| --- \| --- \| --- \| --- \| \| Number of comparisons per family \| 3 \|  \|  \|  \|  \|  \| \| Alpha \| 0.05 \|  \|  \|  \|  \|  \| \|  \|  \|  \|  \|  \|  \|  \| \| Tukey's multiple comparisons test \| Mean Diff. \| 95.00% CI of diff. \| Significant? \| Summary \| Adjusted P Value \|  \| \|  \|  \|  \|  \|  \|  \|  \| \| Veh vs. LPS \| -138.6 \| -198.6 to -78.55 \| Yes \| **** \| <0.0001 \| E-F \| \| Veh vs. LPS  Varlitinib \| -15.25 \| -77.29 to 46.79 \| No \| ns \| 0.8288 \| E-G \| \| LPS vs. LPS  Varlitinib \| 123.3 \| 63.3 to 183.3 \| Yes \| **** \| <0.0001 \| F-G \| |
| **Figure 5D GFAP^+^ area-DG** |
| \| Number of families \| 1 \|  \|  \|  \|  \|  \| \| --- \| --- \| --- \| --- \| --- \| --- \| --- \| \| Number of comparisons per family \| 3 \|  \|  \|  \|  \|  \| \| Alpha \| 0.05 \|  \|  \|  \|  \|  \| \|  \|  \|  \|  \|  \|  \|  \| \| Tukey's multiple comparisons test \| Mean Diff. \| 95.00% CI of diff. \| Significant? \| Summary \| Adjusted P Value \|  \| \|  \|  \|  \|  \|  \|  \|  \| \| Veh vs. LPS \| -275.9 \| -377.6 to -174.2 \| Yes \| **** \| <0.0001 \| I-J \| \| Veh vs. LPS  Varlitinib \| -41.77 \| -146.9 to 63.37 \| No \| ns \| 0.6134 \| I-K \| \| LPS vs. LPS  Varlitinib \| 234.1 \| 132.4 to 335.8 \| Yes \| **** \| <0.0001 \| J-K \| |
| **Figure 6B IL-1β Fluorescence intensity-Cortex** |
| \| Number of families \| 1 \|  \|  \|  \|  \|  \| \| --- \| --- \| --- \| --- \| --- \| --- \| --- \| \| Number of comparisons per family \| 3 \|  \|  \|  \|  \|  \| \| Alpha \| 0.05 \|  \|  \|  \|  \|  \| \|  \|  \|  \|  \|  \|  \|  \| \| Tukey's multiple comparisons test \| Mean Diff. \| 95.00% CI of diff. \| Significant? \| Summary \| Adjusted P Value \|  \| \|  \|  \|  \|  \|  \|  \|  \| \| Veh vs. LPS \| -54.48 \| -70.39 to -38.57 \| Yes \| **** \| <0.0001 \| A-B \| \| Veh vs. LPS Varlitinib \| -10.61 \| -26.2 to 4.984 \| No \| ns \| 0.2426 \| A-C \| \| LPS vs. LPS Varlitinib \| 43.87 \| 28.53 to 59.22 \| Yes \| **** \| <0.0001 \| B-C \| |
| **Figure 6B IL-1β Fluorescence intensity-CA1** |
| \| Number of families \| 1 \|  \|  \|  \|  \|  \| \| --- \| --- \| --- \| --- \| --- \| --- \| --- \| \| Number of comparisons per family \| 3 \|  \|  \|  \|  \|  \| \| Alpha \| 0.05 \|  \|  \|  \|  \|  \| \|  \|  \|  \|  \|  \|  \|  \| \| Tukey's multiple comparisons test \| Mean Diff. \| 95.00% CI of diff. \| Significant? \| Summary \| Adjusted P Value \|  \| \|  \|  \|  \|  \|  \|  \|  \| \| Veh vs. LPS \| -32.56 \| -44.97 to -20.15 \| Yes \| **** \| <0.0001 \| D-E \| \| Veh vs. LPS Varlitinib \| 4.931 \| -7.645 to 17.51 \| No \| ns \| 0.6214 \| D-F \| \| LPS vs. LPS Varlitinib \| 37.49 \| 25.08 to 49.9 \| Yes \| **** \| <0.0001 \| E-F \| |
| **Figure 6B IL-1β Fluorescence intensity-DG** |
| \| Number of families \| 1 \|  \|  \|  \|  \|  \| \| --- \| --- \| --- \| --- \| --- \| --- \| --- \| \| Number of comparisons per family \| 3 \|  \|  \|  \|  \|  \| \| Alpha \| 0.05 \|  \|  \|  \|  \|  \| \|  \|  \|  \|  \|  \|  \|  \| \| Tukey's multiple comparisons test \| Mean Diff. \| 95.00% CI of diff. \| Significant? \| Summary \| Adjusted P Value \|  \| \|  \|  \|  \|  \|  \|  \|  \| \| Veh vs. LPS \| -38.03 \| -48.75 to -27.3 \| Yes \| **** \| <0.0001 \| G-H \| \| Veh vs. LPS Varlitinib \| -2.384 \| -13.32 to 8.553 \| No \| ns \| 0.8627 \| G-I \| \| LPS vs. LPS Varlitinib \| 35.64 \| 24.92 to 46.37 \| Yes \| **** \| <0.0001 \| H-I \| |
| **Figure 6D NLRP3 mRNA-Cortex** |
| \| Number of families \| 1 \|  \|  \|  \|  \|  \| \| --- \| --- \| --- \| --- \| --- \| --- \| --- \| \| Number of comparisons per family \| 3 \|  \|  \|  \|  \|  \| \| Alpha \| 0.05 \|  \|  \|  \|  \|  \| \|  \|  \|  \|  \|  \|  \|  \| \| Tukey's multiple comparisons test \| Mean Diff. \| 95.00% CI of diff. \| Significant? \| Summary \| Adjusted P Value \|  \| \|  \|  \|  \|  \|  \|  \|  \| \| Veh vs. LPS \| -21.36 \| -39.03 to -3.683 \| Yes \| * \| 0.0140 \| A-B \| \| Veh vs. LPS Varlitinib \| -5.245 \| -23.74 to 13.25 \| No \| ns \| 0.7754 \| A-C \| \| LPS vs. LPS Varlitinib \| 16.11 \| 0.3751 to 31.85 \| Yes \| * \| 0.0436 \| B-C \| |
| **Figure 6D NLRP3-CA1** |
| \| Number of families \| 1 \|  \|  \|  \|  \|  \| \| --- \| --- \| --- \| --- \| --- \| --- \| --- \| \| Number of comparisons per family \| 3 \|  \|  \|  \|  \|  \| \| Alpha \| 0.05 \|  \|  \|  \|  \|  \| \|  \|  \|  \|  \|  \|  \|  \| \| Tukey's multiple comparisons test \| Mean Diff. \| 95.00% CI of diff. \| Significant? \| Summary \| Adjusted P Value \|  \| \|  \|  \|  \|  \|  \|  \|  \| \| Veh vs. LPS \| 5.841 \| -11.87 to 23.56 \| No \| ns \| 0.7095 \| D-E \| \| Veh vs. LPS Varlitinib \| 18.74 \| 0.3942 to 37.08 \| Yes \| * \| 0.0442 \| D-F \| \| LPS vs. LPS Varlitinib \| 12.89 \| -3.558 to 29.35 \| No \| ns \| 0.1525 \| E-F \| |
| **Figure 6D NLRP3-DG** |
| \| Number of families \| 1 \|  \|  \|  \|  \|  \| \| --- \| --- \| --- \| --- \| --- \| --- \| --- \| \| Number of comparisons per family \| 3 \|  \|  \|  \|  \|  \| \| Alpha \| 0.05 \|  \|  \|  \|  \|  \| \|  \|  \|  \|  \|  \|  \|  \| \| Tukey's multiple comparisons test \| Mean Diff. \| 95.00% CI of diff. \| Significant? \| Summary \| Adjusted P Value \|  \| \|  \|  \|  \|  \|  \|  \|  \| \| Veh vs. LPS \| 2.555 \| -11.31 to 16.42 \| No \| ns \| 0.8980 \| G-H \| \| Veh vs. LPS Varlitinib \| 11.59 \| -2.764 to 25.95 \| No \| ns \| 0.1364 \| G-I \| \| LPS vs. LPS Varlitinib \| 9.039 \| -3.841 to 21.92 \| No \| ns \| 0.2190 \| H-I \| |
| **Figure 6C IL-1β mRNA-Cortex** |
| \| Number of families \| 1 \|  \|  \|  \|  \|  \| \| --- \| --- \| --- \| --- \| --- \| --- \| --- \| \| Number of comparisons per family \| 3 \|  \|  \|  \|  \|  \| \| Alpha \| 0.05 \|  \|  \|  \|  \|  \| \|  \|  \|  \|  \|  \|  \|  \| \| Tukey's multiple comparisons test \| Mean Diff. \| 95.00% CI of diff. \| Significant? \| Summary \| Adjusted P Value \|  \| \|  \|  \|  \|  \|  \|  \|  \| \| - vs. + \| -79.87 \| -138.2 to -21.56 \| Yes \| ** \| 0.0065 \| A-B \| \| - vs. + \| -34.56 \| -92.87 to 23.75 \| No \| ns \| 0.3139 \| A-C \| \| + vs. + \| 45.31 \| -13 to 103.6 \| No \| ns \| 0.1474 \| B-C \| |
| **Figure 6C IL-1β mRNA-Hippocampus** |
| \| Number of families \| 1 \|  \|  \|  \|  \|  \| \| --- \| --- \| --- \| --- \| --- \| --- \| --- \| \| Number of comparisons per family \| 3 \|  \|  \|  \|  \|  \| \| Alpha \| 0.05 \|  \|  \|  \|  \|  \| \|  \|  \|  \|  \|  \|  \|  \| \| Tukey's multiple comparisons test \| Mean Diff. \| 95.00% CI of diff. \| Significant? \| Summary \| Adjusted P Value \|  \| \|  \|  \|  \|  \|  \|  \|  \| \| - vs. + \| -459 \| -797.9 to -120 \| Yes \| ** \| 0.0072 \| E-F \| \| - vs. + \| -77.34 \| -404.8 to 250.1 \| No \| ns \| 0.8230 \| E-G \| \| + vs. + \| 381.6 \| 42.68 to 720.6 \| Yes \| * \| 0.0257 \| F-G \| |
| **Figure 6D Nlrp3 mRNA-Cortex** |
| \| Number of families \| 1 \|  \|  \|  \|  \|  \| \| --- \| --- \| --- \| --- \| --- \| --- \| --- \| \| Number of comparisons per family \| 3 \|  \|  \|  \|  \|  \| \| Alpha \| 0.05 \|  \|  \|  \|  \|  \| \|  \|  \|  \|  \|  \|  \|  \| \| Tukey's multiple comparisons test \| Mean Diff. \| 95.00% CI of diff. \| Significant? \| Summary \| Adjusted P Value \|  \| \|  \|  \|  \|  \|  \|  \|  \| \| - vs. + \| -3.75 \| -6.376 to -1.124 \| Yes \| ** \| 0.0046 \| A-B \| \| - vs. + \| -1.115 \| -3.741 to 1.511 \| No \| ns \| 0.5426 \| A-C \| \| + vs. + \| 2.635 \| 0.00933 to 5.262 \| Yes \| * \| 0.0491 \| B-C \| |
| **Figure 6D Nlrp3 mRNA-Hippocampus** |
| \| Number of families \| 1 \|  \|  \|  \|  \|  \| \| --- \| --- \| --- \| --- \| --- \| --- \| --- \| \| Number of comparisons per family \| 3 \|  \|  \|  \|  \|  \| \| Alpha \| 0.05 \|  \|  \|  \|  \|  \| \|  \|  \|  \|  \|  \|  \|  \| \| Tukey's multiple comparisons test \| Mean Diff. \| 95.00% CI of diff. \| Significant? \| Summary \| Adjusted P Value \|  \| \|  \|  \|  \|  \|  \|  \|  \| \| - vs. + \| -5.802 \| -9.95 to -1.654 \| Yes \| ** \| 0.0056 \| E-F \| \| - vs. + \| -4.133 \| -8.14 to -0.1257 \| Yes \| * \| 0.0425 \| E-G \| \| + vs. + \| 1.669 \| -2.479 to 5.817 \| No \| ns \| 0.5744 \| F-G \| |
| **Figure 7B Iba-1 Fluorescence intensity-Cortex** |
| \| Table Analyzed \| IBa-1 \| \| --- \| --- \| \|  \|  \| \| Column B \| Varlitinib \| \| vs. \| vs. \| \| Column A \| Veh \| \|  \|  \| \| Unpaired t test \|  \| \| P value \| <0.0001 \| \| P value summary \| **** \| \| Significantly different (P < 0.05)? \| Yes \| \| One- or two-tailed P value? \| Two-tailed \| \| t, df \| t=4.62 df=87 \| |
| **Figure 7B Iba-1 Fluorescence intensity-CA1** |
| \| Table Analyzed \| IBa-1 \| \| --- \| --- \| \|  \|  \| \| Column D \| Varlitinib \| \| vs. \| vs. \| \| Column C \| Veh \| \|  \|  \| \| Unpaired t test \|  \| \| P value \| <0.0001 \| \| P value summary \| **** \| \| Significantly different (P < 0.05)? \| Yes \| \| One- or two-tailed P value? \| Two-tailed \| \| t, df \| t=4.469 df=86 \| |
| **Figure 7B Iba-1 Fluorescence intensity-DG** |
| \| Table Analyzed \| IBa-1 \| \| --- \| --- \| \|  \|  \| \| Column F \| Varlitinib \| \| vs. \| vs. \| \| Column E \| Veh \| \|  \|  \| \| Unpaired t test \|  \| \| P value \| 0.0009 \| \| P value summary \| *** \| \| Significantly different (P < 0.05)? \| Yes \| \| One- or two-tailed P value? \| Two-tailed \| \| t, df \| t=3.45 df=86 \| |
| **Figure 7B Iba-1^+^ cells-Cortex** |
| \| Table Analyzed \| IBa-1 \| \| --- \| --- \| \|  \|  \| \| Column B \| Varlitinib \| \| vs. \| vs. \| \| Column A \| Veh \| \|  \|  \| \| Unpaired t test \|  \| \| P value \| <0.0001 \| \| P value summary \| **** \| \| Significantly different (P < 0.05)? \| Yes \| \| One- or two-tailed P value? \| Two-tailed \| \| t, df \| t=4.838 df=87 \| |
| **Figure 7B Iba-1^+^ cells-CA1** |
| \| Table Analyzed \| IBa-1 \| \| --- \| --- \| \|  \|  \| \| Column D \| Varlitinib \| \| vs. \| vs. \| \| Column C \| Veh \| \|  \|  \| \| Unpaired t test \|  \| \| P value \| <0.0001 \| \| P value summary \| **** \| \| Significantly different (P < 0.05)? \| Yes \| \| One- or two-tailed P value? \| Two-tailed \| \| t, df \| t=4.254 df=86 \| |
| **Figure 7B Iba-1^+^ cells-DG** |
| \| Table Analyzed \| IBa-1 \| \| --- \| --- \| \|  \|  \| \| Column F \| Varlitinib \| \| vs. \| vs. \| \| Column E \| Veh \| \|  \|  \| \| Unpaired t test \|  \| \| P value \| 0.0018 \| \| P value summary \| ** \| \| Significantly different (P < 0.05)? \| Yes \| \| One- or two-tailed P value? \| Two-tailed \| \| t, df \| t=3.224 df=86 \| |
| **Figure 7B Iba-1^+^ area-Cortex** |
| \| Table Analyzed \| IBa-1 \| \| --- \| --- \| \|  \|  \| \| Column B \| Varlitinib \| \| vs. \| vs. \| \| Column A \| Veh \| \|  \|  \| \| Unpaired t test \|  \| \| P value \| 0.0002 \| \| P value summary \| *** \| \| Significantly different (P < 0.05)? \| Yes \| \| One- or two-tailed P value? \| Two-tailed \| \| t, df \| t=3.826 df=87 \| |
| **Figure 7B Iba-1^+^ area-CA1** |
| \| Table Analyzed \| IBa-1 \| \| --- \| --- \| \|  \|  \| \| Column D \| Varlitinib \| \| vs. \| vs. \| \| Column C \| Veh \| \|  \|  \| \| Unpaired t test \|  \| \| P value \| 0.0013 \| \| P value summary \| ** \| \| Significantly different (P < 0.05)? \| Yes \| \| One- or two-tailed P value? \| Two-tailed \| \| t, df \| t=3.329 df=86 \| |
| **Figure 7B Iba-1^+^ area-DG** |
| \| Table Analyzed \| IBa-1 \| \| --- \| --- \| \|  \|  \| \| Column F \| Varlitinib \| \| vs. \| vs. \| \| Column E \| Veh \| \|  \|  \| \| Unpaired t test \|  \| \| P value \| 0.0065 \| \| P value summary \| ** \| \| Significantly different (P < 0.05)? \| Yes \| \| One- or two-tailed P value? \| Two-tailed \| \| t, df \| t=2.791 df=86 \| |
| **Figure 7D GFAP^+^ Fluorescence intensity-Cortex** |
| \| Table Analyzed \| fluorescence intensity \| \| --- \| --- \| \|  \|  \| \| Column B \| Varlitinib \| \| vs. \| vs. \| \| Column A \| Veh \| \|  \|  \| \| Unpaired t test \|  \| \| P value \| 0.0957 \| \| P value summary \| ns \| \| Significantly different (P < 0.05)? \| No \| \| One- or two-tailed P value? \| Two-tailed \| \| t, df \| t=1.722 df=29 \| |
| **Figure 7D GFAP^+^ Fluorescence intensity-CA1** |
| \| Table Analyzed \| fluorescence intensity \| \| --- \| --- \| \|  \|  \| \| Column D \| Varlitinib \| \| vs. \| vs. \| \| Column C \| Veh \| \|  \|  \| \| Unpaired t test \|  \| \| P value \| 0.0225 \| \| P value summary \| * \| \| Significantly different (P < 0.05)? \| Yes \| \| One- or two-tailed P value? \| Two-tailed \| \| t, df \| t=2.402 df=31 \| |
| **Figure 7D GFAP^+^ Fluorescence intensity-DG** |
| \| Table Analyzed \| fluorescence intensity \| \| --- \| --- \| \|  \|  \| \| Column F \| Varlitinib \| \| vs. \| vs. \| \| Column E \| Veh \| \|  \|  \| \| Unpaired t test \|  \| \| P value \| 0.0114 \| \| P value summary \| * \| \| Significantly different (P < 0.05)? \| Yes \| \| One- or two-tailed P value? \| Two-tailed \| \| t, df \| t=2.689 df=31 \| |
| **Figure 7D GFAP^+^ Cells-Cortex** |
| \| Table Analyzed \| Cell counts \| \| --- \| --- \| \|  \|  \| \| Column B \| Varlitinib \| \| vs. \| vs. \| \| Column A \| Veh \| \|  \|  \| \| Unpaired t test \|  \| \| P value \| 0.0599 \| \| P value summary \| ns \| \| Significantly different (P < 0.05)? \| No \| \| One- or two-tailed P value? \| One-tailed \| \| t, df \| t=1.603 df=29 \| |
| **Figure 7D GFAP^+^ Cells-CA1** |
| \| Table Analyzed \| Cell counts \| \| --- \| --- \| \|  \|  \| \| Column D \| Varlitinib \| \| vs. \| vs. \| \| Column C \| Veh \| \|  \|  \| \| Unpaired t test \|  \| \| P value \| 0.2095 \| \| P value summary \| ns \| \| Significantly different (P < 0.05)? \| No \| \| One- or two-tailed P value? \| Two-tailed \| \| t, df \| t=1.281 df=31 \| |
| **Figure 7D GFAP^+^ Cells-DG** |
| \| Table Analyzed \| Cell counts \| \| --- \| --- \| \|  \|  \| \| Column F \| Varlitinib \| \| vs. \| vs. \| \| Column E \| Veh \| \|  \|  \| \| Unpaired t test \|  \| \| P value \| 0.1111 \| \| P value summary \| ns \| \| Significantly different (P < 0.05)? \| No \| \| One- or two-tailed P value? \| Two-tailed \| \| t, df \| t=1.64 df=31 \| |
| **Figure 7D GFAP^+^ % area-Cortex** |
| \| Table Analyzed \| % Area \| \| --- \| --- \| \|  \|  \| \| Column B \| Varlitinib \| \| vs. \| vs. \| \| Column A \| Veh \| \|  \|  \| \| Unpaired t test \|  \| \| P value \| 0.2149 \| \| P value summary \| ns \| \| Significantly different (P < 0.05)? \| No \| \| One- or two-tailed P value? \| Two-tailed \| \| t, df \| t=1.268 df=29 \| |
| **Figure 7D GFAP^+^ % area -CA1** |
| \| Table Analyzed \| % Area \| \| --- \| --- \| \|  \|  \| \| Column D \| Varlitinib \| \| vs. \| vs. \| \| Column C \| Veh \| \|  \|  \| \| Unpaired t test \|  \| \| P value \| 0.0169 \| \| P value summary \| * \| \| Significantly different (P < 0.05)? \| Yes \| \| One- or two-tailed P value? \| Two-tailed \| \| t, df \| t=2.524 df=31 \| |
| **Figure 7D GFAP^+^ % area -DG** |
| \| Table Analyzed \| % Area \| \| --- \| --- \| \|  \|  \| \| Column F \| Varlitinib \| \| vs. \| vs. \| \| Column E \| Veh \| \|  \|  \| \| Unpaired t test \|  \| \| P value \| 0.0061 \| \| P value summary \| ** \| \| Significantly different (P < 0.05)? \| Yes \| \| One- or two-tailed P value? \| Two-tailed \| \| t, df \| t=2.942 df=31 \| |
| **Figure 8B AT100 Fluorescence intensity-Cortex** |
| \| Table Analyzed \| AT100 \| \| --- \| --- \| \|  \|  \| \| Column B \| Varlitinib \| \| vs. \| vs. \| \| Column A \| Veh \| \|  \|  \| \| Unpaired t test \|  \| \| P value \| 0.0005 \| \| P value summary \| *** \| \| Significantly different (P < 0.05)? \| Yes \| \| One- or two-tailed P value? \| Two-tailed \| \| t, df \| t=3.662 df=72 \| |
| **Figure 8B AT100 Fluorescence intensity-CA1** |
| \| Table Analyzed \| AT100 \| \| --- \| --- \| \|  \|  \| \| Column D \| Varlitinib \| \| vs. \| vs. \| \| Column C \| Veh \| \|  \|  \| \| Unpaired t test \|  \| \| P value \| <0.0001 \| \| P value summary \| **** \| \| Significantly different (P < 0.05)? \| Yes \| \| One- or two-tailed P value? \| Two-tailed \| \| t, df \| t=5.005 df=74 \| |
| **Figure 8B AT100 Fluorescence intensity-DG** |
| \| Table Analyzed \| AT100 \| \| --- \| --- \| \|  \|  \| \| Column F \| Varlitinib \| \| vs. \| vs. \| \| Column E \| Veh \| \|  \|  \| \| Unpaired t test \|  \| \| P value \| <0.0001 \| \| P value summary \| **** \| \| Significantly different (P < 0.05)? \| Yes \| \| One- or two-tailed P value? \| Two-tailed \| \| t, df \| t=5.401 df=74 \| |
| **Figure 8D AT180 Fluorescence intensity-Cortex** |
| \| Table Analyzed \| AT180 \| \| --- \| --- \| \|  \|  \| \| Column B \| Varlitinib \| \| vs. \| vs. \| \| Column A \| Veh \| \|  \|  \| \| Unpaired t test \|  \| \| P value \| 0.3665 \| \| P value summary \| ns \| \| Significantly different (P < 0.05)? \| No \| \| One- or two-tailed P value? \| Two-tailed \| \| t, df \| t=0.9077 df=87 \| |
| **Figure 8D AT180 Fluorescence intensity-CA1** |
| \| Table Analyzed \| AT180 \| \| --- \| --- \| \|  \|  \| \| Column D \| Varlitinib \| \| vs. \| vs. \| \| Column C \| Veh \| \|  \|  \| \| Unpaired t test \|  \| \| P value \| 0.1425 \| \| P value summary \| ns \| \| Significantly different (P < 0.05)? \| No \| \| One- or two-tailed P value? \| Two-tailed \| \| t, df \| t=1.48 df=87 \| |
| **Figure 8D AT180 Fluorescence intensity-DG** |
| \| Table Analyzed \| AT180 \| \| --- \| --- \| \|  \|  \| \| Column F \| Varlitinib \| \| vs. \| vs. \| \| Column E \| Veh \| \|  \|  \| \| Unpaired t test \|  \| \| P value \| 0.0387 \| \| P value summary \| * \| \| Significantly different (P < 0.05)? \| Yes \| \| One- or two-tailed P value? \| One-tailed \| \| t, df \| t=1.787 df=87 \| |
| **Figure 8F DYRK1A Fluorescence intensity-Cortex** |
| \| Table Analyzed \| DYRK1A \| \| --- \| --- \| \|  \|  \| \| Column B \| Varlitinib \| \| vs. \| vs. \| \| Column A \| Veh \| \|  \|  \| \| Unpaired t test \|  \| \| P value \| <0.0001 \| \| P value summary \| **** \| \| Significantly different (P < 0.05)? \| Yes \| \| One- or two-tailed P value? \| Two-tailed \| \| t, df \| t=4.375 df=76 \| |
| **Figure 8F DYRK1A Fluorescence intensity-CA1** |
| \| Table Analyzed \| DYRK1A \| \| --- \| --- \| \|  \|  \| \| Column D \| Varlitinib \| \| vs. \| vs. \| \| Column C \| Veh \| \|  \|  \| \| Unpaired t test \|  \| \| P value \| <0.0001 \| \| P value summary \| **** \| \| Significantly different (P < 0.05)? \| Yes \| \| One- or two-tailed P value? \| Two-tailed \| \| t, df \| t=6.399 df=76 \| |
| **Figure 8F DYRK1A Fluorescence intensity-DG** |
| \| Table Analyzed \| DYRK1A \| \| --- \| --- \| \|  \|  \| \| Column F \| Varlitinib \| \| vs. \| vs. \| \| Column E \| Veh \| \|  \|  \| \| Unpaired t test \|  \| \| P value \| <0.0001 \| \| P value summary \| **** \| \| Significantly different (P < 0.05)? \| Yes \| \| One- or two-tailed P value? \| Two-tailed \| \| t, df \| t=6.648 df=76 \| |
| **Supplementary Figure 1B IL-β Fluorescence intensity-Cortex** |
| \| Table Analyzed \| IL-1b \| \| --- \| --- \| \|  \|  \| \| Column B \| Varlitinib \| \| vs. \| vs. \| \| Column A \| Veh \| \|  \|  \| \| Unpaired t test \|  \| \| P value \| 0.3151 \| \| P value summary \| ns \| \| Significantly different (P < 0.05)? \| No \| \| One- or two-tailed P value? \| Two-tailed \| \| t, df \| t=1.018 df=38 \| |
| **Supplementary Figure 1B IL-β Fluorescence intensity-CA1** |
| \| Table Analyzed \| IL-1b \| \| --- \| --- \| \|  \|  \| \| Column D \| Varlitinib \| \| vs. \| vs. \| \| Column C \| Veh \| \|  \|  \| \| Unpaired t test \|  \| \| P value \| 0.1617 \| \| P value summary \| ns \| \| Significantly different (P < 0.05)? \| No \| \| One- or two-tailed P value? \| Two-tailed \| \| t, df \| t=1.427 df=38 \| |
| **Supplementary Figure 1B IL-β Fluorescence intensity-DG** |
| \| Table Analyzed \| IL-1b \| \| --- \| --- \| \|  \|  \| \| Column F \| Varlitinib \| \| vs. \| vs. \| \| Column E \| Veh \| \|  \|  \| \| Unpaired t test \|  \| \| P value \| 0.1730 \| \| P value summary \| ns \| \| Significantly different (P < 0.05)? \| No \| \| One- or two-tailed P value? \| Two-tailed \| \| t, df \| t=1.389 df=38 \| |
| **Supplementary Figure 1D NLRP3 Fluorescence intensity-Cortex** |
| \| Table Analyzed \| IL-1b \| \| --- \| --- \| \|  \|  \| \| Column B \| Varlitinib \| \| vs. \| vs. \| \| Column A \| Veh \| \|  \|  \| \| Unpaired t test \|  \| \| P value \| 0.1996 \| \| P value summary \| ns \| \| Significantly different (P < 0.05)? \| No \| \| One- or two-tailed P value? \| Two-tailed \| \| t, df \| t=1.309 df=33 \| |
| **Supplementary Figure 1D NLRP3 Fluorescence intensity-CA1** |
| \| Table Analyzed \| IL-1b \| \| --- \| --- \| \|  \|  \| \| Column D \| Varlitinib \| \| vs. \| vs. \| \| Column C \| Veh \| \|  \|  \| \| Unpaired t test \|  \| \| P value \| 0.1283 \| \| P value summary \| ns \| \| Significantly different (P < 0.05)? \| No \| \| One- or two-tailed P value? \| Two-tailed \| \| t, df \| t=1.558 df=35 \| |
| **Supplementary Figure 1D NLRP3 Fluorescence intensity-DG** |
| \| Table Analyzed \| IL-1b \| \| --- \| --- \| \|  \|  \| \| Column F \| Varlitinib \| \| vs. \| vs. \| \| Column E \| Veh \| \|  \|  \| \| Unpaired t test \|  \| \| P value \| 0.2252 \| \| P value summary \| ns \| \| Significantly different (P < 0.05)? \| No \| \| One- or two-tailed P value? \| Two-tailed \| \| t, df \| t=1.235 df=35 \| |
| **Supplementary Figure 1F IL-β Fluorescence intensity-Cortex** |
| \| Table Analyzed \| IL-1b \| \| --- \| --- \| \|  \|  \| \| Column B \| Varlitinib \| \| vs. \| vs. \| \| Column A \| Veh \| \|  \|  \| \| Unpaired t test \|  \| \| P value \| 0.1996 \| \| P value summary \| ns \| \| Significantly different (P < 0.05)? \| No \| \| One- or two-tailed P value? \| Two-tailed \| \| t, df \| t=1.309 df=33 \| |
| **Supplementary Figure 1F IL-β Fluorescence intensity-CA1** |
| \| Table Analyzed \| IL-1b \| \| --- \| --- \| \|  \|  \| \| Column D \| Varlitinib \| \| vs. \| vs. \| \| Column C \| Veh \| \|  \|  \| \| Unpaired t test \|  \| \| P value \| 0.1283 \| \| P value summary \| ns \| \| Significantly different (P < 0.05)? \| No \| \| One- or two-tailed P value? \| Two-tailed \| \| t, df \| t=1.558 df=35 \| |
| **Supplementary Figure 1F IL-β Fluorescence intensity-DG** |
| \| Table Analyzed \| IL-1b \| \| --- \| --- \| \|  \|  \| \| Column F \| Varlitinib \| \| vs. \| vs. \| \| Column E \| Veh \| \|  \|  \| \| Unpaired t test \|  \| \| P value \| 0.2252 \| \| P value summary \| ns \| \| Significantly different (P < 0.05)? \| No \| \| One- or two-tailed P value? \| Two-tailed \| \| t, df \| t=1.235 df=35 \| |
| **Supplementary Figure 1H NLRP3 Fluorescence intensity-Cortex** |
| \| Table Analyzed \| NLRP3 \| \| --- \| --- \| \|  \|  \| \| Column B \| Varlitinib \| \| vs. \| vs. \| \| Column A \| Veh \| \|  \|  \| \| Unpaired t test \|  \| \| P value \| 0.4665 \| \| P value summary \| ns \| \| Significantly different (P < 0.05)? \| No \| \| One- or two-tailed P value? \| Two-tailed \| \| t, df \| t=0.735 df=41 \| |
| **Supplementary Figure 1H NLRP3 Fluorescence intensity-CA1** |
| \| Table Analyzed \| NLRP3 \| \| --- \| --- \| \|  \|  \| \| Column D \| Varlitinib \| \| vs. \| vs. \| \| Column C \| Veh \| \|  \|  \| \| Unpaired t test \|  \| \| P value \| 0.1173 \| \| P value summary \| ns \| \| Significantly different (P < 0.05)? \| No \| \| One- or two-tailed P value? \| Two-tailed \| \| t, df \| t=1.601 df=40 \| |
| **Supplementary Figure 1H NLRP3 Fluorescence intensity-DG** |
| \| Table Analyzed \| NLRP3 \| \| --- \| --- \| \|  \|  \| \| Column F \| Varlitinib \| \| vs. \| vs. \| \| Column E \| Veh \| \|  \|  \| \| Unpaired t test \|  \| \| P value \| 0.0862 \| \| P value summary \| ns \| \| Significantly different (P < 0.05)? \| No \| \| One- or two-tailed P value? \| Two-tailed \| \| t, df \| t=1.76 df=39 \| |
| **Supplementary Figure 2B p-CDK5 Fluorescence intensity-Cortex** |
| \| Table Analyzed \| pCDK5 \| \| --- \| --- \| \|  \|  \| \| Column B \| Varlitinib \| \| vs. \| vs. \| \| Column A \| Veh \| \|  \|  \| \| Unpaired t test \|  \| \| P value \| 0.0724 \| \| P value summary \| ns \| \| Significantly different (P < 0.05)? \| No \| \| One- or two-tailed P value? \| Two-tailed \| \| t, df \| t=1.837 df=48 \| |
| **Supplementary Figure 2B p-CDK5 Fluorescence intensity-CA1** |
| \| Table Analyzed \| pCDK5 \| \| --- \| --- \| \|  \|  \| \| Column D \| Varlitinib \| \| vs. \| vs. \| \| Column C \| Veh \| \|  \|  \| \| Unpaired t test \|  \| \| P value \| 0.4279 \| \| P value summary \| ns \| \| Significantly different (P < 0.05)? \| No \| \| One- or two-tailed P value? \| Two-tailed \| \| t, df \| t=0.7994 df=50 \| |
| **Supplementary Figure 2B p-CDK5 Fluorescence intensity-DG** |
| \| Table Analyzed \| pCDK5 \| \| --- \| --- \| \|  \|  \| \| Column F \| Varlitinib \| \| vs. \| vs. \| \| Column E \| Veh \| \|  \|  \| \| Unpaired t test \|  \| \| P value \| 0.7535 \| \| P value summary \| ns \| \| Significantly different (P < 0.05)? \| No \| \| One- or two-tailed P value? \| Two-tailed \| \| t, df \| t=0.3157 df=50 \| |
| **Supplementary Figure B p-CDK5 Fluorescence intensity-Cortex** |
| \| Table Analyzed \| pGSK3b \| \| --- \| --- \| \|  \|  \| \| Column B \| Varlitinib \| \| vs. \| vs. \| \| Column A \| Veh \| \|  \|  \| \| Unpaired t test \|  \| \| P value \| 0.8233 \| \| P value summary \| ns \| \| Significantly different (P < 0.05)? \| No \| \| One- or two-tailed P value? \| Two-tailed \| \| t, df \| t=0.2245 df=49 \| |
| **Supplementary Figure B p-CDK5 Fluorescence intensity-CA1** |
| \| Table Analyzed \| pGSK3b \| \| --- \| --- \| \|  \|  \| \| Column D \| Varlitinib \| \| vs. \| vs. \| \| Column C \| Veh \| \|  \|  \| \| Unpaired t test \|  \| \| P value \| 0.5355 \| \| P value summary \| ns \| \| Significantly different (P < 0.05)? \| No \| \| One- or two-tailed P value? \| Two-tailed \| \| t, df \| t=0.6245 df=45 \| |
| **Supplementary Figure B p-CDK5 Fluorescence intensity-DG** |
| \| Table Analyzed \| pGSK3b \| \| --- \| --- \| \|  \|  \| \| Column F \| Varlitinib \| \| vs. \| vs. \| \| Column E \| Veh \| \|  \|  \| \| Unpaired t test \|  \| \| P value \| 0.3335 \| \| P value summary \| ns \| \| Significantly different (P < 0.05)? \| No \| \| One- or two-tailed P value? \| Two-tailed \| \| t, df \| t=0.9776 df=45 \| |
